# *N*-Sulfinylpyrrolidine-containing ureas and thioureas as bifunctional organocatalysts

**DOI:** 10.3762/bjoc.17.176

**Published:** 2021-10-25

**Authors:** Viera Poláčková, Dominika Krištofíková, Boglárka Némethová, Renata Górová, Mária Mečiarová, Radovan Šebesta

**Affiliations:** 1 Department of Organic Chemistry,Faculty of Natural Sciences, Comenius University in Bratislava, Mlynská dolina, Ilkovičova 6, 842 15 Bratislava, Slovakia; 2 Department of Analytical Chemistry, Faculty of Natural Sciences, Comenius University in Bratislava, Mlynská dolina, Ilkovičova 6, 842 15 Bratislava, Slovakia

**Keywords:** asymmetric organocatalysis, hydrogen bond, Michael addition, pyrrolidine, thiourea, urea

## Abstract

The synthesis of bifunctional *N*-sulfinylureas and thioureas with an appended pyrrolidine unit is presented. These organocatalysts were evaluated in Michael additions of aldehydes to nitroalkenes both under solvent-free conditions and in solution. The *N*-sulfinylurea catalyst was more efficient than the corresponding thiourea. For some substrates, enantioselectivities reached 98% ee. The stereogenic center on the sulfur did not have a considerable influence on the catalytic reactions. Under ball-milling conditions, the Michael adducts were obtained in good yields but with slightly lower enantiomeric purities than in solution. DFT calculations elucidated its mode of action and confirmed a dual activation mode, which combines enamine activation of aldehydes and hydrogen-bond activation of nitroalkenes.

## Introduction

Asymmetric organocatalysis became one of the strategic ways for the efficient synthesis of chiral compounds [1]. Bifunctional catalysis has proven to be a successful concept in asymmetric organocatalysis [2–8]. An amine unit with a hydrogen-bond donating skeleton is highly efficient from among various possible combinations of catalytic moieties within an organocatalyst. This idea has been inspired by proline catalysis itself, in which the carboxylic function acts as an ancillary hydrogen-bond donor for the direction of one of the reagents [9]. Amines serve as basic units and nucleophilic components capable of carbonyl compounds activation via enamine or iminium ion formation [10–11]. In particular, pyrrolidine became a privileged structural motif central to many catalyst designs [12]. This fact stems from the success of diarylprolinol silyl ethers as chiral organocatalysts, which were independently introduced by Hayashi [13] and Jørgensen [14]. These compounds were used in a large number of stereoselective syntheses, including total syntheses of natural compounds [15]. The pyrrolidine moiety has been successfully combined with thiourea [16–18] and the squaramide unit [19–20]. Thioureas and squaramides often feature the electron-withdrawing group attached to one of the nitrogen atoms to increase the acidity of the hydrogen-bond donating unit. This notion has often been realized with substituted aryls such as 3,5-bis(trifluoromethyl)phenyl. Ellman introduced a different approach and developed *N*-sulfinylureas. An additional potentially useful feature is the stereogenic center on sulfur. *tert*-Butanesulfinamide is highly useful in stereoselective synthesis as a stereoinducing group [21]. Thus, *N*-sulfinylureas and thioureas are a new class of organocatalysts, with the sulfinyl group acting both as an acidifying and a chiral controlling moiety. A variety of *N*-sulfinylureas catalyzed aza-Henry reaction, including enantioselective H-bonding-catalyzed additions to aliphatic *N*-Boc-imines with high stereoselectivity [22]. A broad range of β-aminonitroolefins were reduced to chiral β-aminonitroalkanes in high yields and excellent enantioselectivities using trichlorosilane as a reducing agent and an *N*-sulfinylurea as bifunctional organocatalyst [23]. The enantio- and diastereoselective addition of Meldrum’s acids to nitroalkenes via *N*-sulfinylurea catalysis gave products that were readily converted to pharmaceutically relevant compounds [24–25]. A sulfinylurea organocatalyst catalyzed a highly selective Michael addition of thioacetic acid to aromatic and aliphatic nitroalkenes to produce chiral β-aminothiols, compounds of pharmaceutical interest [26]. Similarly, the enantioselective addition of thioacids to trisubstituted nitroalkenes was catalyzed by several *N*-sulfinylureas providing the 1,2-nitrothioacetates in good yields and enantioselectivities [27]. A sulfinylurea catalyst was also applied to catalyze the addition of 3-substituted pyrazol-5-ones to trisubstituted nitroalkenes. The adducts were obtained with good yields and enantioselectivities up to 91:9 er [28].

**Figure 1:**
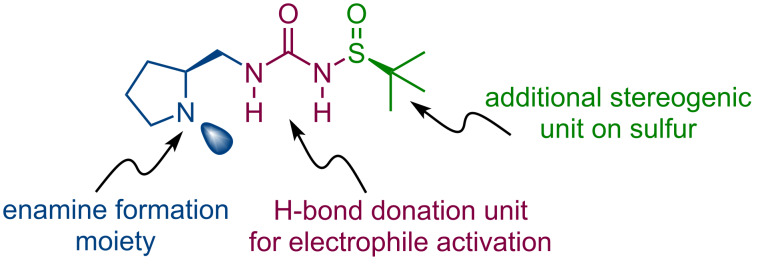
Catalyst design principles.

Inspired by the previous successful applications of sulfinylureas and thioureas as organocatalysts, we have designed four new *N*-sulfinyl-*N*’-(pyrrolidinylmethyl)urea and *N*-sulfinyl-*N*’-(pyrrolidinylmethyl)thiourea bifunctional organocatalysts. The main design principles are outlined in Figure 1. The catalysts feature a pyrrolidine unit, which should engage in enamine activation of enolizable carbonyl compounds. The urea or thiourea moiety shall provide hydrogen-bond donating ability. Furthermore, these compounds possess a sulfinyl group with an additional stereogenic center on the sulfur. To verify the influence of a matched/mismatched combination of chirality, we employed both enantiomers of *tert*-butyl sulfinamide with the (*S*)-enantiomer of the pyrrolidine building block.

The introduction of green chemistry principles into chemical transformations is an important goal toward sustainable production and manufacturing. Asymmetric organocatalysis can benefit and accommodate many sustainability techniques [29]. Mechanochemistry can increase the sustainability profile of a chemical process by reducing potentially harmful organic solvents and bring other benefits such as substantially shortened reaction times. A handful of asymmetric organocatalytic transformations were successfully performed under solvent-free ball-milling conditions [30–31]. In this context, we describe the synthesis of new pyrrolidine appended sulfinylurea and thiourea organocatalysts and their assessment in Michael additions of aldehydes to nitroalkenes. Furthermore, we have evaluated the suitability of these catalysts under solvent-free conditions. With the help of DFT calculations, we elucidated the mode of action of these catalysts.

## Results and Discussion

### Synthesis of catalysts

**Scheme 1:**
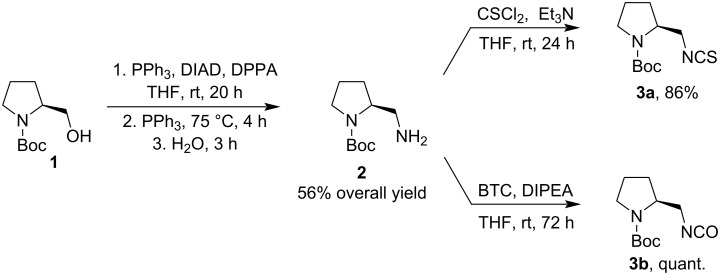
Synthesis of isothiocyanate **3a** and isocyanate **3b**.

We have started the synthesis of the catalysts from Boc-protected (*S*)-prolinol (**1**), from which the key intermediate, pyrrolidine derivative **2**, can be obtained in three steps according to the literature procedure [32]. Using this method, we obtained the product **2** in a yield comparable (56% overall yield) to that described in the literature. However, the difficult chromatographic separation after each step prompted us to apply a Mitsunobu and Staudinger reaction for the preparation of amine **2** (Scheme 1) [33]. This one-pot reaction gave the desired amine **2** in 56% yield. Then, the corresponding isothiocyanate **3a** was prepared by reaction of amine **2** with CS_2_ and DCC according to the reported procedure. However, this method gave product **3a** in only 44% yield. Therefore, we decided to prepare isothiocyanate **3a** using thiophosgene in dry THF with Et_3_N. This procedure afforded the corresponding isothiocyanate **3a** in 86% yield (Scheme 1). Isocyanate **3b** was also synthesized from amine **2**. The reaction with bis(trichloromethyl)carbonate (BTC) afforded the crude product **3b**, which was sufficiently pure for use in the next reaction step without further purification (Scheme 1).

**Scheme 2:**
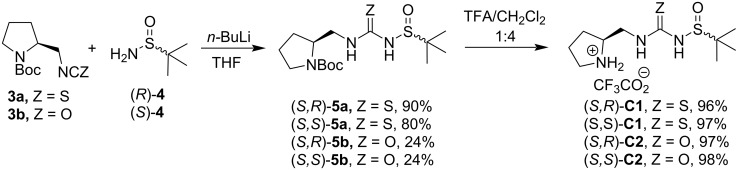
Synthesis of sulfinylthioureas **C1** and ureas **C2**.

The next steps of the catalyst synthesis were the attachment of *tert*-butanesulfinamide **4** to iso(thio)cyanates **3a** and **3b** with concomitant formation of the urea or thiourea moiety, respectively. The corresponding *N*-Boc-protected precursors of the desired catalysts, **5a** and **5b**, were obtained in low to good yields. The removal of the Boc-protecting group with trifluoroacetic acid afforded the desired *N*-sulfinylthioureas (*S*,*R*)- and (*S*,*S*)-**C1** as well as *N*-sulfinylureas (*S*,*R*)- and (*S*,*S*)-**C2** in excellent yields (Scheme 2).

### Application of thioureas **C1** and ureas **C2** in the Michael addition of aldehydes to nitroalkenes

#### Michael addition in solution

**Scheme 3:**
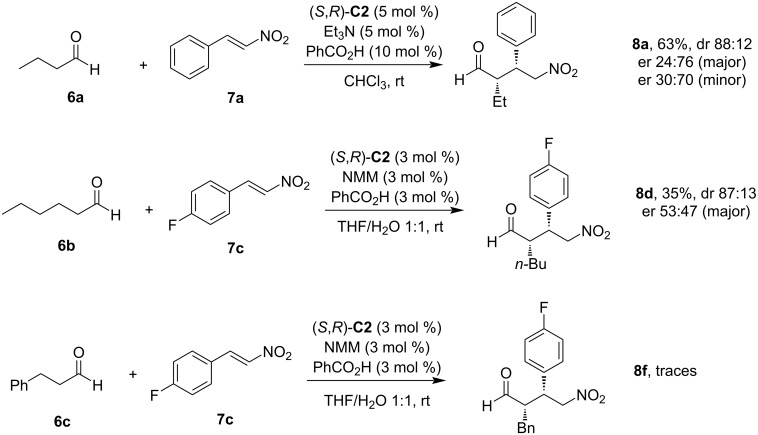
Synthesis of adducts **8a**,**d**,**f** in solution.

As the first benchmark transformation, we opted for the Michael addition of butanal (**6a**) to β-nitrostyrene (**7a**) catalyzed by (*S*,*R*)-**C2** (Scheme 3). The reaction in CH_2_Cl_2_ at 5 °C with Et_3_N as a base gave 45% of adduct **8a** with 86:14 dr and 24:76 er for both diastereomers. Slightly better yields (63%) were achieved in CHCl_3_ at room temperature with Et_3_N or NMP as a base, but both diastereoselectivity as well as enantioselectivity remained unchanged. We have used thiourea (*S,R*)-**C1** for this Michael addition, too, but the catalyst was not successful for this reaction (not shown).

Only traces of the Michael adduct were obtained in the solution reaction of butanal (**6a**) with 1-methoxy-4-(2-nitrovinyl)benzene (**7b**). Hexanal (**6b**) reacted successfully with 4-fluoro-β-nitrostyrene (**7c**) and gave product **8d** under all conditions tested (in solution, solvent-free, and ball-milling conditions, vide infra). Again, small amounts of catalyst (*S,R*)-**C2** gave the best chemical yield. Catalyst (*S,R*)-**C2** (3 mol %) in solution (NMM as the base, THF/H_2_O 1:1) provided the product in only 35% yield, but with high diastereomeric purity of 87:13 dr. However, this result could not be obtained with thiourea (*S,R*)-**C1**, which provided only traces of product **8d**. The Michael addition was not successful when 3-phenylpropanal (**6c**) was reacted with 4-fluoro-β-nitrostyrene (**7c**). In the presence of catalyst (*S,R*)-**C2** only traces of product **8f** (THF/H_2_O, NMM as the base, and additive PhCO_2_H) were obtained (Scheme 3).

Michael acceptors containing heterocyclic groups have been studied only sparingly, but the corresponding chiral compounds with heterocyclic substituents are of high biological and medicinal relevance [34–35]. Therefore, we have decided to evaluate sulfinylurea and thiourea catalysts **C1** and **C2** also with (*E*)-2-(2-nitrovinyl)furan (**9**) and (*E*)-3-(2-nitrovinyl)pyridine (**11**) as Michael acceptors. As Michael donor, we chose 3-phenylpropanal (**6c**).

The Michael addition of 3-phenylpropanal (**6c**) with (*E*)-2-(2-nitrovinyl)furan (**9**) under initial reaction conditions with (*S,R*)-**C1** (10 mol %) in THF/H_2_O with NMM as the base and with PhCO_2_H as acid additive gave product **10a** in 31% yield after 72 hours with a diastereomeric ratio of 86:14 and high enantiomeric purity of 95:5 er for the major diastereomer (Table 1, entry 1). Using chloroform/isopropyl alcohol 9:1 as the solvent mixture afforded after 120 hours, aldehyde **10a** in 45% yield with 83:17 dr and 97:3 er (Table 1, entry 2). The Michael addition in methanol catalyzed by only 3 mol % (*S,R*)-**C1** after 72 hours provided only 18% yield, but with high enantiomeric purity (99:1, Table 1, entry 5). The reaction without a base did not provide the desired product **10a** (Table 1, entry 6). Moreover, a reaction performed with other acidic additives (phenylboronic acid, *p*-toluenesulfonic acid) provided after 72 hours only 18% and 23% yield of the product with compromised diastereomeric and enantiomeric purity (Table 1, entries 3 and 4). When, we applied 3 mol % of catalyst (*S,R*)-**C1** during 48 hours, we obtained 73% yield with diastereomeric purity 83:17 and high enantiomeric purity, similar to the reaction performed in methanol (99:1 er, Table 1, entry 7). Using the same conditions as with catalyst (*S,R*)-**C1**, we also used 3 mol % (*S,S*)-**C1** (Table 1, entry 8). The yield and diastereomeric and enantiomeric purity were very similar as with catalyst (*S,R*)-**C1**. However, a further reduction of the catalyst loading to 1 mol % of (*S,S*)-**C1**, required a longer reaction time, up to 216 hours and this Michael addition gave only 27% yield of the product (Table 1, entry 11). Additionally, attempting the Michael addition of 3-phenylpropanal (**6c**) to nitroalkene **9** catalyzed by (*S,S*)-**C1** without any acid additive resulted in a very low yield after 48 hours (29%, Table 1, entry 9) with a diastereomeric purity of 80:20 dr. We also tested the Boc-protected derivative (*S,S*)-**5b** as the catalyst (Table 1, entry 10). The Michael addition catalyzed by (*S,S*)-**5b** provided racemic product **10a** in 23% yield. This result confirms the essential role of the pyrrolidine unit in the enamine formation during the reaction. Michael addition reactions catalyzed with sulfinylureas (*S,R*)-**C2** and (*S,S*)-**C2** provided the products within 24 hours in good yields (63% and 88%, respectively) but with lowered diastereomeric and enantiomeric purities (Table 1, entries 12 and 13).

**Table 1:** Michael additions of aldehydes **6b**–**d** with nitroalkene **9**.

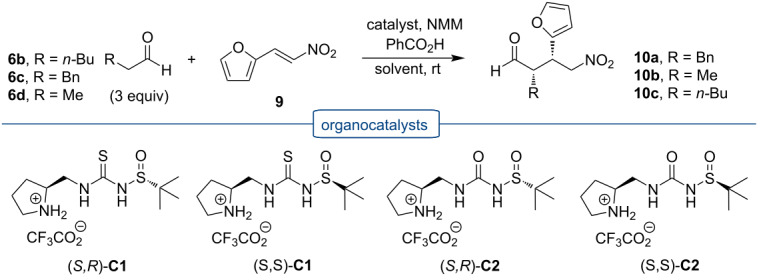						

entry	catalyst (mol %)^a^	solvent	time (h)	yield of **10** (%)	dr	er major/minor

1	(*S,R*)-**C1** (10)	THF/H_2_O 1:1	72	31 (**10a**)	86:14	95:5/98:2
2	(*S,R*)-**C1** (10)	CHCl_3_/iPrOH 9:1	120	45 (**10a**)	83:17	97:3/97:3
3	(*S,R*)-**C1** (3)^b^	THF/H_2_O 4:1	72	23 (**10a**)	67:33	n.d.
4	(*S,R*)-**C1** (3)^c^	THF/H_2_O 4:1	72	18 (**10a**)	50:50	n.d.
5	(*S,R*)-**C1** (3)	MeOH	72	18 (**10a**)	88:12	99:1/99:1
6	(*S,R*)-**C1** (3)^d^	MeOH	72	– (**10a**)	–	–
7	(*S,R*)-**C1** (3)	THF/H_2_O 1:1	48	73 (**10a**)	83:17	99:1/99:1
8	(*S,S*)-**C1** (3)	THF/H_2_O 1:1	48	72 (**10a**)	89:11	99:1/99:1
9	(*S,S*)-**C1** (3)^e^	THF/H_2_O 1:1	48	29 (**10a**)	80:20	n.d.
10	(*S,S*)-**5b** (3)	THF/H_2_O 1:1	48	23 (**10a**)	55:45	50:50
11	(*S,S*)-**C1** (1)	THF/H_2_O 1:1	216	27 (**10a**)	86:14	n.d.
12	(*S,R*)-**C2** (3)	THF/H_2_O 1:1	24	63 (**10a**)	86:14	68:32/85:15
13	(*S,S*)-**C2** (3)	THF/H_2_O 1:1	24	88 (**10a**)	88:12	70:30/87:13
14	(*S,R*)-**C2** (3)	THF/H_2_O 1:1	24	70 (**10b**)	85:15	75:25/73:27
15	(*S,R*)-**C2** (3)	THF/H_2_O, no acid	24	73 (**10b**)	87:13	73:27/75:25
16	(*S,R*)-**C2** (3)	THF/H_2_O, no base	24	25 (**10b**)	87:13	74:26/71:29
17	(*S,R*)-**C2** (3)	THF/H_2_O, no acid, no base	24	44 (**10b**)	86:14	73:27/71:29
18	(*S,R*)-**C2** (3)	THF/H_2_O 1:1	24	40 (**10c**)	77:23	86:14

^a^Catalyst, *N*-methylmorpholine (NMM) and acid loading was the same; ^b^PhB(OH)_2_ was used instead of PhCO_2_H; ^c^*p*TSA was used instead of PhCO_2_H; ^d^the reaction was performed without any basic additive; ^e^the reaction was performed without any acid additive.

In terms of the stereochemical outcome, both sulfinylthioureas **C1** and urea **C2** afforded the same enantiomer as the main product. Furthermore, both diastereomers of both catalysts also directed the Michael addition toward the same enantiomer. These results suggest that the main stereogenic element in the catalyst structure is the pyrrolidine unit. The stereogenic center on the sulfur plays only a minor role, probably because it is far away from the reaction center.

Catalyst (*S,R*)-**C2** catalyzed the Michael addition of propanal (**6d**) and hexanal (**6b**) to nitroalkene **9**. The reaction in the presence of 3 mol % (*S,R*)-**C2** provided the product **10b** in 70% yield and 85:15 dr and 75:25 er (Table 1, entry 14). Here, we have also tested the influence of only basic additive on the reaction and the product was obtained with 73% yield (Table 1, entry 15). The reaction without a base went much less efficiently (Table 1, entry 16), similarly to the reaction performed without acid additive and base (Table 1, entry 17). The product **10c** by Michael addition of hexanal **6b** to nitroalkene **9** was obtained with only 40% yield with comparable diastereoselectivity (Table 1, entry 18). The aliphatic aldehydes propanal (**6d**) and hexanal (**6b**) provided medium yields and diastereoselectivity and enantioselectivity.

The Michael addition of 3-phenylpropanal (**6c**) to (*E*)-3-(2-nitrovinyl)pyridine (**11**) required long reaction times (120 h) in solution, similar to those for the reaction with (*E*)-2-(2-nitrovinyl)furan (**9**) and they provided racemic adduct **12** in 14 or 64% yield with poor or no diastereoselectivity (Table 2, entries 1 and 2). The change of solvent made it possible to obtain the products in a shorter time. Reactions catalyzed with 3 mol % (*S,R*)-**C1** and (*S,S*)-**C1** in MeCN gave product **12** in 38 or 39% yield with dr 80:20 and 88:12 and er 38:62 and 39:61 (Table 2, entries 3 and 4). Slightly higher yields and similar diastereolectivities were achieved with urea-derived catalysts (*S,R*)-**C2** and (*S,S*)-**C2**, but nitroaldehyde **12** was obtained in racemic form (Table 2, entries 5 and 6).

**Table 2:** Michael addition of 3-phenylpropanal (**6c**) to nitroalkene **11**.

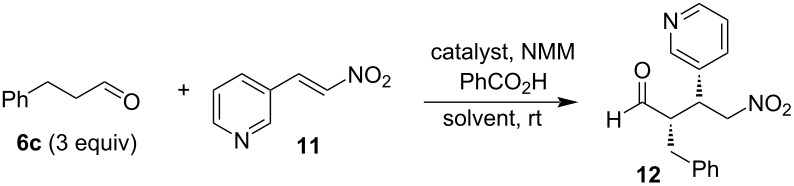						

entry	catalyst (mol %)^a^	solvent	time (h)	yield of **12** (%)^b^	dr	er major/minor

1	(*S,R*)-**C1** (15)	CHCl_3_/iPrOH 9:1	120	14	56:44	50:50/50:50
2	(*S,R*)-**C1** (10)	THF/H_2_O 4:1	120	64	67:33	50:50/50:50
3	(*S,R*)-**C1** (3)	MeCN	22	38	80:20	38:62/57:43
4	(*S,S*)-**C1** (3)	MeCN	48	39	88:12	39:61/60:40
5	(*S,R*)-**C2** (3)	MeCN	22	56	81:19	49:51/51:49
6	(*S,S*)-**C2** (3)	MeCN	22	65	80:20	48:52/50:50

^a^Catalyst, *N*-methylmorpholine (NMM) and acid loading was the same.

#### Michael additions under solvent-free reaction conditions

To evaluate the applicability of the new catalysts **C1** and **C2**, we decided to test them in the Michael addition under solvent-free conditions. Ball-milling experiments were conducted in a mixer mill, in which the milling vessels perform radial oscillations with vibrational frequencies from 3 to 30 Hz. These reactions were realized in stainless steel milling jars with an internal volume of 5 mL and with stainless steel balls (Ø 5 mm). We have started with an evaluation of the solvent-free conditions for the reaction of butanal (**6a**) and nitrostyrene (**7a**) using sulfinylurea catalyst (*S,R*)-**C2**.

A relatively high yield (81%) of Michael adduct **8a** was formed in 3 hours of milling, with triethylamine as the base (Table 3, entry 1). The diastereoselectivity and enantioselectivity reached comparable values as in the solvent conditions. The chemical yield of adduct **8a** dropped to 51%, when the excess of butanal (**6a**) was reduced from 3 to 1.5 equivalents. The diastereoselectivity increased to 93:7 and the enantioselectivity for the major enantiomer was 19:81 and 16:84 for the minor enantiomer, respectively (Table 3, entry 2). A base exchange had no significant influence, neither on yields nor on selectivities. Reactions under ball milling with *N*-methylpyrrole (NMP), iPr_2_EtN, DABCO, K_3_PO_4_·3H_2_O, *N*-methylmorpholine (NMM) (Table 3, entries 3–8) proceeded with yields of 53–82%. The highest value of diastereoselectivity was achieved only with triethylamine as the base (dr 93:7) but unfortunately with a comparable enantioselectivity (Table 3, entry 2). When the excess of butanal (**6a**) was reduced from 3 to 1.5 equivalents, the yield again decreased (Table 3, cf. entries 2 and 7). The Michael addition of aldehyde **6a** to nitroalkene **7a** with K_2_CO_3_ and pyrrole (10 mol %) as the base, respectively, afforded adduct **8a** in 71 and 75% yield, with diastereoselectivity of 60:40 and 62:38 and in a racemic form (Table 3, entries 9 and 10). Only traces of adduct **8a** were detected in the reaction mixture when the reaction in the ball mill was carried out without any base and any acid additive (Table 3, entry 11).

**Table 3:** Optimization of reaction conditions for solvent-free Michael additions.^a^

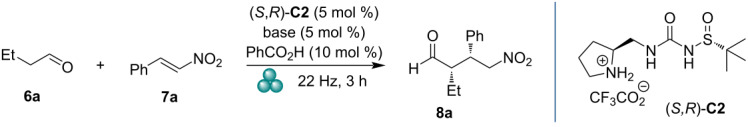				

entry	base	yield of **8a** (%)	dr	er (major/minor)

1	Et_3_N	81	84:16	26:74/28:72
2^b^	Et_3_N	51	93:7	19:81/16:84
3	NMP	59	83:17	24:76/20:80
4	iPr_2_EtN	77	75:25	24:76/29:71
5	DABCO	66	80:20	23:77/22:78
6	K_3_PO_4_·3H_2_O	82	86:14	25:75/27:73
7^b^	K_3_PO_4_·3H_2_O	53	86:14	22:78/19:81
8	NMM	70	71:29	33:67/28:72
9	K_2_CO_3_	71	60:40	45:55/51:49
10	pyrrole	75	62:38	52:48/54:46
11	–	traces^c^	–	–

^a^Reaction conditions: the catalyst (0.016 mmol), base (0.016 mmol), nitroalkene (0.33 mmol), butyraldehyde (1 mmol), benzoic acid (0.03 mmol) and NaCl (1.2 g) were added to ball mill reactor in one portion, milling frequency 22 Hz, milling time 3 h; ^b^1.5 equiv of aldehyde **6a**; ^c^reaction proceeded without any base and acid.

Furthermore, we have continued with the evaluation of catalyst (*S,R*)-**C2** in the Michael addition of aldehydes **6a**–**c** to functionalized nitrostyrenes **7b** and **7c**. These reactions were conducted using a ball-milling set-up as well as solvent-free stirring at 30 °C. The experimental results of the addition reactions of aldehydes **6a**–**c** with nitrostyrenes **7b**,**c** catalyzed with (*S,R*)-**C2** are summarized in Table 4.

**Table 4:** Michael addition of aldehyde **6a**–**c** to nitroalkenes **7a** and **7b**.^a^

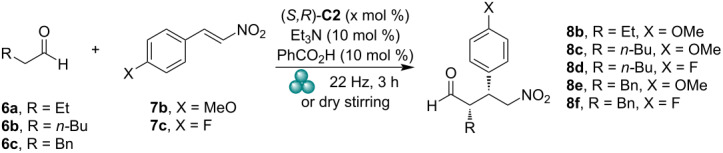					

entry	catalyst loading (mol %)	method	yield (%)	dr	er (major/minor)

1	5	ball-milling	32 (**8b**)	64:36	52:48/54:46
2	2.5	dry stirring (4 d)	75 (**8c**)	71:29	33:67/70:30
3	5	dry stirring (48 h)	67 (**8c**)	55:45	50:50/50:50
4	10	dry stirring (48 h)	32 (**8c**)	64:36	35:65/62:38
5	2.5	dry stirring (48 h)	70 (**8d**)	64:36	35:65/65:35
6	5	dry stirring (72 h)	67 (**8d**)	72:28	34:66/68:32
7	2.5	ball-milling	66 (**8d**)	71:29	35:65/65:35
8	5	dry stirring (72 h)	76 (**8e**)	57:43	33:67/65:35
9	5	dry stirring (72 h)	79 (**8f**)	63:27	36:64/64:36

^a^The catalyst (2.5–10 mol %), base (10 mol %) and a half volume of aldehyde (total 5 equiv used), were added to a 10 mL vial vessel. After 5 min, the remaining volume of aldehyde was added to the mixture. Benzoic acid (10 mol %) was added after 5 min stirring and 10 min later, nitroalkene (1 equiv) was added.

The aliphatic aldehyde **6a** in the Michael addition with 4-methoxy-β-nitrostyrene (**7b**) catalyzed by catalyst (*S,R*)-**C2** gave the corresponding Michael adduct exclusively by using the ball-mill method. The Michael addition was carried out in the presence of Et_3_N as the base and provided only 32% yield of the product with low diastereoselectivity and enantioselectivity (Table 4, entry 1). The aliphatic aldehyde **6b** with 4-methoxy-β-nitrostyrene (**7b**) gave the Michael addition product **8c** by the solvent-free method by stirring at 30 °C. Ten mol % of catalyst (*S,R*)-**C2** gave 32% yield after 48 hours. The best result in terms of yield and diastereoselectivity was obtained by a small amount of catalyst (*S,R*)-**C2**. Already 2.5 mol % of (*S,R*)-**C2** provided the product in 75% yield and 71:29 dr and 33:67 er, respectively. A higher catalyst loading of 5 mol % under solvent-free stirring gave 67% yield and 55:45 dr and 50:50 er (Table 4, entries 2–4). Hexanal (**6b**) also reacted successfully with 4-fluoro-β-nitrostyrene (**7c**) and gave the product **8d** under solvent-free and ball-mill conditions. Again a small amount of catalyst (*S,R*)-**C2** (2.5 mol %) gave the best chemical yield, 70% using solvent-free, neat stirring at 30 °C. In comparison, the ball-mill reaction afforded 66% of the product (Table 4, entries 5–7). The Michael addition of aldehyde **6c** gave under dry stirring products **8e** and **8f** in 76 and 79% yield with comparable diastereoselectivity and enantioselectivity (Table 4, entries 8 and 9).

### DFT calculations of catalyst structure and reaction stereo-course

**Figure 2:**
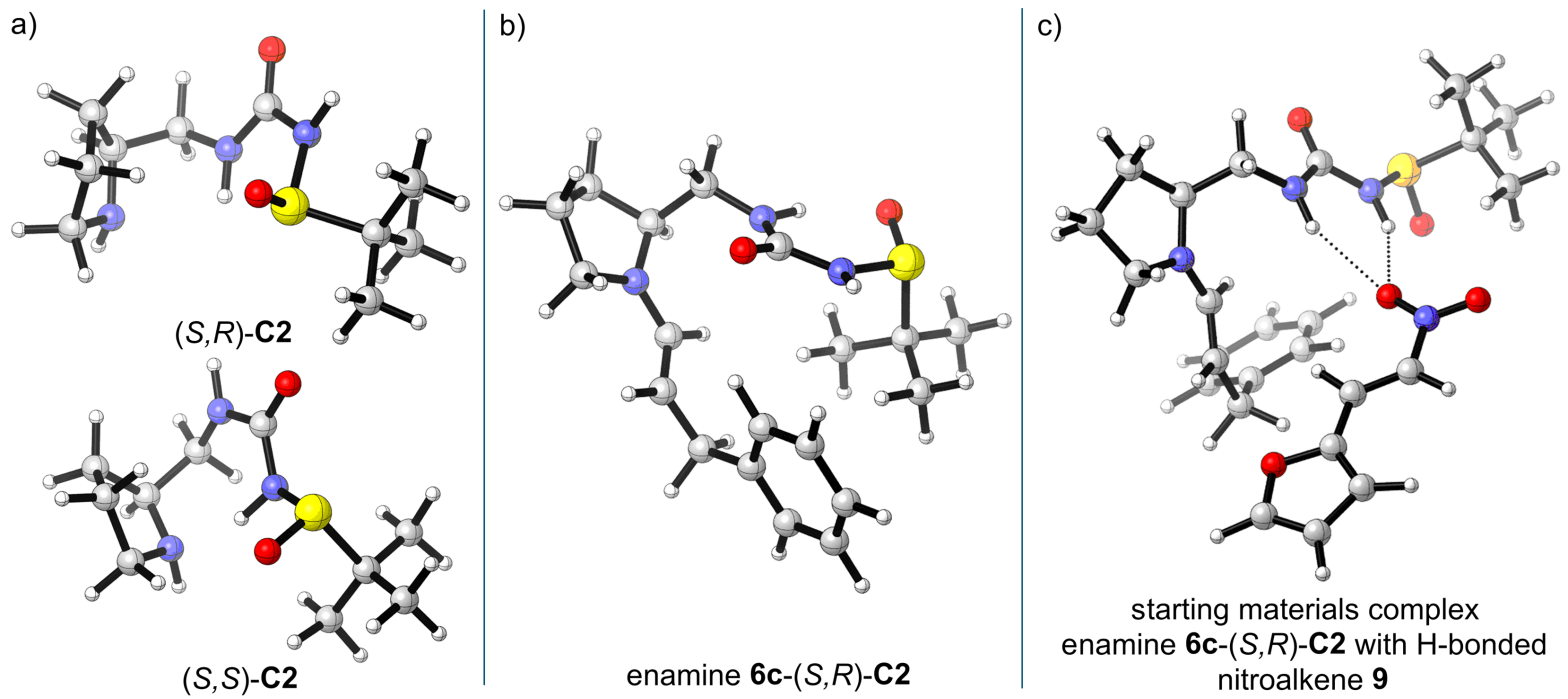
DFT-calculated (PBEh-3c/def2-SV(P)//M06-2X/def2-TZVP) structures of catalyst (*S,R*) and (*S,S*)-**C2**, enamine between aldehyde **6c** and (*S,R*)-**C2**; enamine **6c**-(*S,R*)-**C2** and hydrogen-bonded nitroalkene **9**.

To understand the catalyst operation, we have conducted DFT calculations of its structure and reaction course. All calculations were realized using Turbomole program package [36–37]. Geometric optimizations were performed using PBEh-3c functional [38]. This functional is a composite scheme based on the well-known PBE0 functional [39–40]. PBEh-3c corrects for the basis set superposition error and accounts for the long-range London dispersion interactions. Geometrical optimizations were performed with the Karlsruhe split-valence def2-SV(P) basis set [41]. Energies were refined using the Minnesota M06-2X functional [42] and valence triple-zeta def2-TZVP basis set [43]. The lowest energy conformers of both catalyst (*S,R*)- and (*S,S*)-**C2** (Figure 2a) have *anti*-*syn* arrangement of the urea unit. Figure 2b shows the enamine intermediate likely formed between aldehyde **6c** and catalyst (*S,R*)-**C2**. The urea unit adopts an *anti*-*anti* arrangement upon coordination of a nitroalkene via hydrogen bonds (Figure 2c).

**Figure 3:**
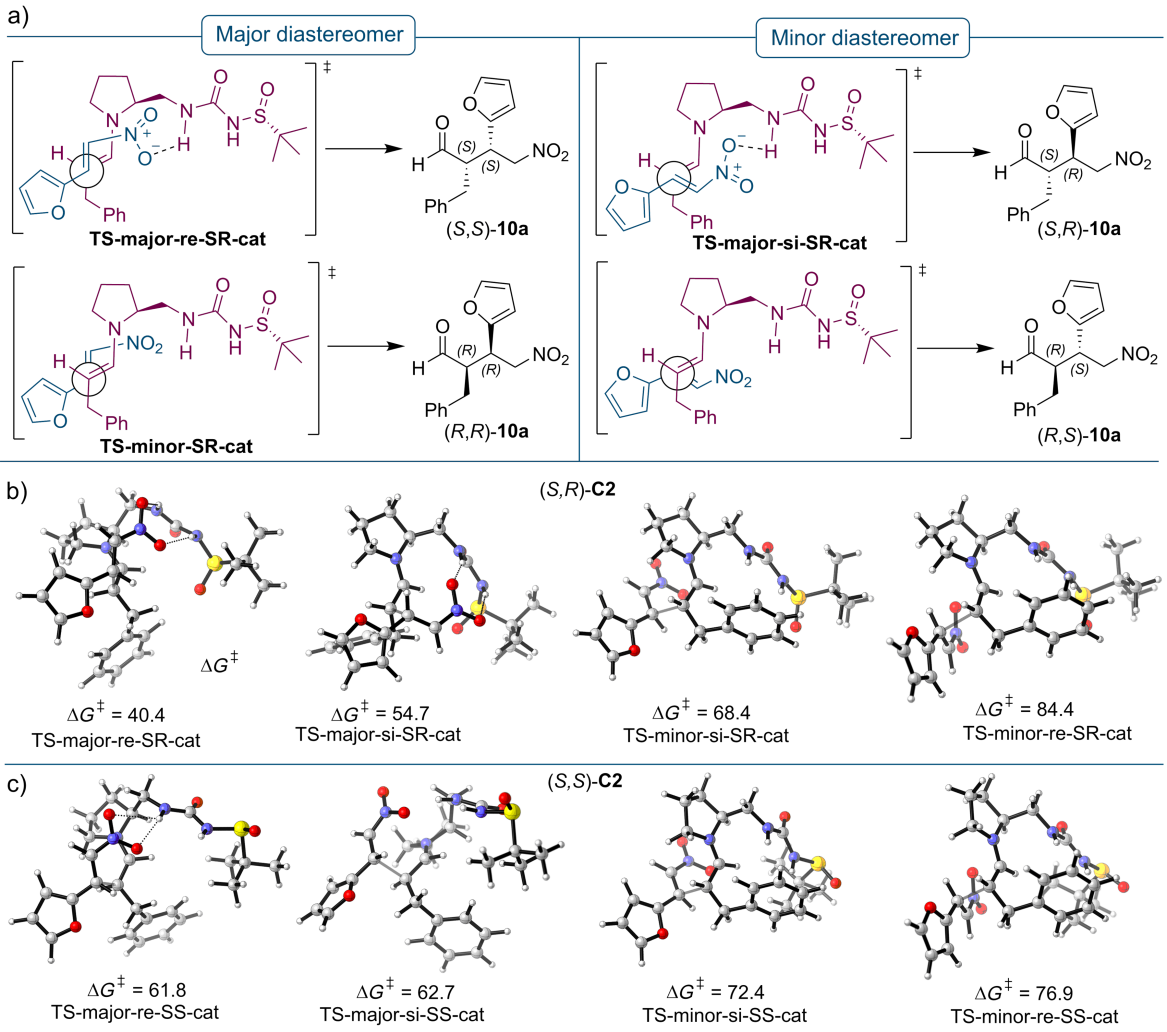
a) Arrangements of reactants in the transition states; b) DFT-calculated (PBEh-3c/def2-SV(P)//M06-2X/def2-TZVP) transition states with catalyst (*S*,*R*)-**C2**; c) calculated transition states with catalyst (*S*,*S*)-**C2**; Gibbs free energies of activation in kJ/mol.

The reaction likely proceeds via initial enamine formation from the aldehyde and catalyst. The coordination of the nitroalkene via hydrogen bonding with the (thio)urea moiety will bring it in the vicinity of the enamine from the *re*-face. The major enantiomer of the Michael adduct (*S*,*S*)-**10** is formed via *re*-attack on the nitroalkene. The nitroalkene is in *synclinal* orientation with respect to the enamine double bond. The alternative *si*-attack on the nitroalkene provides the minor diastereomer (*S*,*R*)-**10**. The enantiomeric products (*R*,*R*)- and (*R*,*S*)-**10** could be formed via the Michael addition from the *si*-face of the enamine. In this case, the nitroalkene could not be activated by hydrogen bonding via the (thio)urea moiety, however, it is also probably less sterically hindered (Figure 3a). The DFT calculated transition states support this analysis. The transition state **TS-major-re-SR-cat** leading to the major stereoisomer of the Michael adducts has the lowest Gibbs free energy of activation of 40.4 kJ·mol^−1^. The Gibbs free energies of activations for the (*S*,*S*)-**C2** catalyst are only slightly higher than those for the (*S*,*R*)-**C2** catalyst. These calculations support the experimental observation that the configuration of the sulfur stereogenic center does not play an important role in the Michael addition (Figure 3b). The stereochemical outcome of the Michael addition is dictated mainly by the configuration of the proline unit. The calculated transition states for the Michael addition with both diastereomeric catalysts (*S,R*)- and (*S,S*)-**C2** are displayed in Figure 3c.

**Figure 4:**
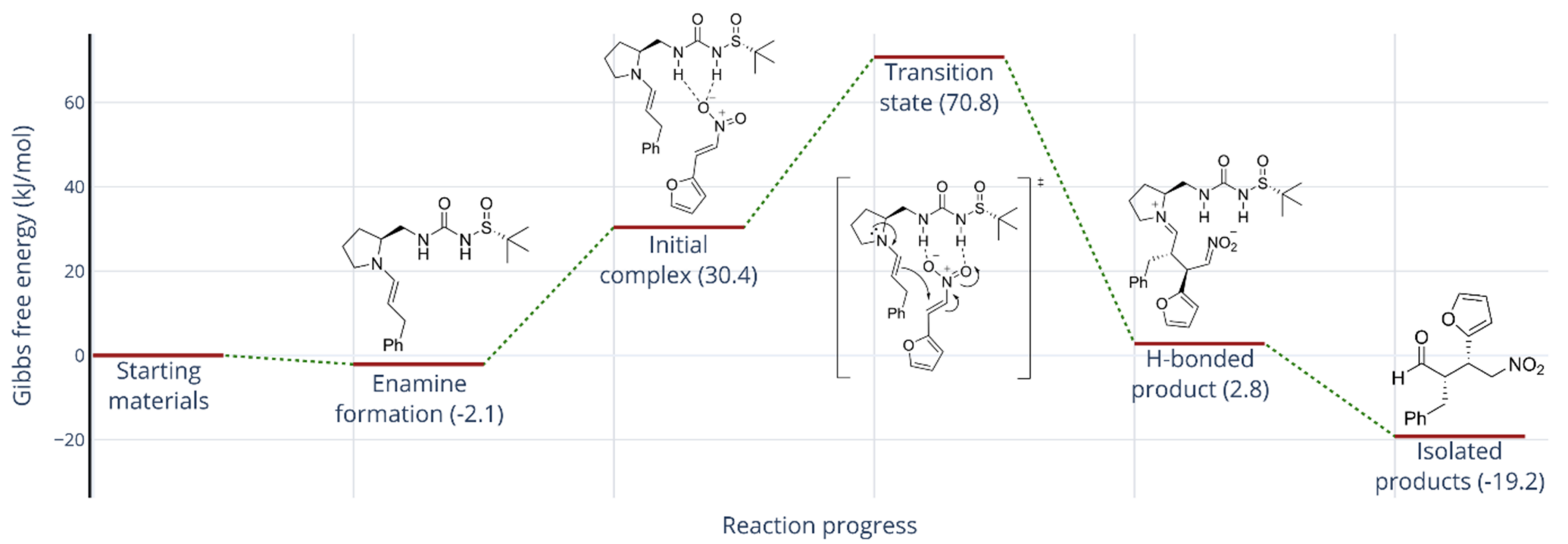
DFT-calculated (PBEh-3c/def2-SV(P)//M06-2X/def2-TZVP) reaction profile for the Michael addition of 3-phenylpropanal (**6c**) and nitroalkene **9** using catalyst (*S,R*)-**C2**.

After the Michael addition, the initial products formed are iminium salts with the catalysts, which are hydrolyzed to the isolated Michael adducts **10**. A representative reaction profile is depicted in Figure 4.

## Conclusion

We have designed and synthesized bifunctional pyrrolidine-containing sulfinylureas and thioureas. These catalysts operate via enamine activation of aldehydes and hydrogen-bond activation of the electrophilic component, in this study – nitrostyrenes. These catalysts were effective in the Michael addition of aldehydes to nitroalkenes, affording the corresponding adducts in medium to high diastereomeric and enantiomeric purities. The reactions under solvent-free conditions performed considerably faster than those under classical conditions in solution, with comparable or better yields, without any significant effect on selectivity. Sulfinylurea catalysts were more active than the corresponding thioureas. The additional stereogenic center on the sulfur plays only a minor role on the stereoselectivity of the reaction, which is governed mainly by the configuration of the proline moiety. DFT calculations elucidated the stereochemical action of the catalysts in organocatalytic Michael addition and suggested the possibilities of further improvement in catalyst design.

## Experimental

### Synthesis of catalysts

#### *(S*)-*tert*-Butyl 2-(aminomethyl)pyrrolidine-1-carboxylate (**2**)

The solution of PPh_3_ (1.64 g, 6.3 mmol) and *N*-Boc-(*S*)-prolinol (**1**, 1.0 g, 5.0 mmol) in dry THF (10 mL) was cooled in an ice-water bath, and subsequently, diisopropyl azodicarboxylate (DIAD, 1.21 g, 6.0 mmol) and diphenylphosphoryl azide (DPPA, 1.65 g, 6.0 mmol) were added dropwise under argon atmosphere. The mixture was allowed to reach room temperature and stirred for 20 h. The reaction mixture was then warmed to 75 °C and refluxed for 2 h, subsequently, PPh_3_ (1.64 g, 6.3 mmol) in THF (10 mL) was added, and the reaction mixture was refluxed for further 2 h. After that, the reaction mixture was cooled to room temperature, water (1 mL) was added, and the mixture was stirred for 3 h. Then, the solvent was removed under vacuum and the pH of the residue was adjusted to around 2 with 1 M HCl. The aqueous phase was washed with Et_2_O (3 × 25 mL). The pH of the aqueous phase was adjusted to 13 with 2 M NaOH, and extracted with DCM (6 × 20 mL). The organic phase was dried with Na_2_SO_4_ and concentrated under reduced pressure to afford the product in 56% yield. *R*_f_ 0.11 (hexane/ethyl acetate 3:1); ^1^H NMR (300 MHz, CD_3_OD) δ 3.82–3,64 (m, 1H), 3.48–3.24 (m, 4H), 2.81 (dd, *J* = 12.7, 4.5 Hz, 1H), 2.57 (dd, *J* = 12.7, 7.8 Hz, 1H), 2.01–1.78 (m, 4H), 1.46 (s, 9H) ppm. Spectral data agree with those in the literature [32].

#### (*S*)-*tert*-Butyl 2-(isothiocyanatomethyl)pyrrolidine-1-carboxylate (**3a**)

The solution of Et_3_N (0.13 g, 1.3 mmol) and *(S*)-*tert*-butyl 2-(aminomethyl)pyrrolidine-1-carboxylate (**2**, 0.08 g, 0.4 mmol) in dry THF (4 mL) was cooled in an ice–water bath and next it was added dropwise into cooled CSCl_2_ (0.12 g, 1.1 mmol) under argon atmosphere. The reaction mixture was stirred for 30 min in an ice–water bath and 24 h at room temperature. Cold water (60 mL) was then added, and the aqueous phase was extracted with Et_2_O (3 × 40 mL). The combined organic phase was washed with aq saturated solution of NaHCO_3_ (3 × 40 mL), dried over Na_2_SO_4_, and concentrated under reduced pressure. The residue was purified by column chromatography on silica gel (eluent, hexane/ethyl actate 7:1→5:1), affording the product as dark orange oil in 86% yield. *R*_f_ 0.5 (hexane/EtOAc 3:1); IR (ATR): 2971, 2089, 1390, 1700, 1162 cm^−1^. ^1^H NMR (300 MHz, CDCl_3_) δ 3.98–3.84 (m, 2H); 3.68–3.57 (m, 1H), 3.53–3.35 (m, 2H), 2.13–2.03 (m, 1H), 1.99–1.81 (m, 3H), 1,47 (s, 9H) ppm.

#### *(S)-tert*-Butyl 2-(isocyanatomethyl)pyrrolidine-1-carboxylate (**3b**)

BTC (0.33 g, 1.11 mmol) was dissolved in dry THF (10 mL) and the solution was cooled to 0 °C. Then, *N,N*-diisopropylethylamine (DIPEA, 1.14 g, 8.84 mmol) was added dropwise, followed by a solution of *(S*)-*tert*-butyl 2-(aminomethyl)pyrrolidine-1-carboxylate (**2**, 0.44 g, 2.21 mmol) in dry THF (10 mL) during 30 min. The reaction mixture was stirred for 72 h at room temperature. The solvent was removed in vacuo and the residue was dissolved in DCM (60 mL) and washed with 0.1 M HCl (2 × 30 mL). The organic phase was dried over Na_2_SO_4_ and the crude reaction mixture was used in the next reaction step without further purification.

#### General procedure for preparation of *N*-sulfinylthiourea pre-catalysts (*S*,*R*)-**5a** and (*S*,*S*)-**5a**

A stirred solution of *(R)-tert-*butanesulfinamide or *(S)-tert-*butanesulfinamide (0.09 g, 0.75 mmol) in THF (5 mL) was cooled to 0 °C under argon atmosphere. Butyllithium in hexane (0.35 g, 0.82 mmol) was added dropwise, and the solution was stirred for 15 min. The cooling bath was removed and the solution of (*S*)-*tert*-butyl 2-(isothiocyanatomethyl)pyrrolidine-1-carboxylate (**3a**, 0.20 g, 0.82 mmol) in dry THF (5 mL) was added dropwise over 15 min and stirring continued at rt for four days. The reaction was quenched with water (0.3 mL) and the mixture was stirred for 30 min. The resulting mixture was concentrated in vacuo and the desired product was isolated by column chromatography on silica gel (EtOAc/MeOH/NH_4_OH 60:1:0.6→50:1:0.5).

#### *(S*)-*tert*-Butyl 2-((3-((*R*)-*tert*-butylsulfinyl)thioureido)methyl)pyrrolidine-1-carboxylate ((*S*,*R*)-**5a**)


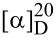
 −87.8 (*c* 1.0, MeOH); ^1^H NMR (300 MHz, CDCl_3_) δ 9.17 (s, 1H), 6.85 (s, 1H), 4.17–4.09 (m, 1H), 3.83–3.72 (m, 1H), 3.44–3.32 (m, 3H), 2.10–2.00 (m, 1H), 1.97–1.82 (m, 2H), 1.77–1.64 (m, 1H), 1.49 (s, 9H), 1.31 (s, 9H); ^13^C NMR (75 MHz, CDCl_3_) δ 181.9, 157.2, 80.8, 57.6, 55.8, 53.3,47.3, 29.5, 28.5, 23.9, 22.1 ppm; IR (ATR): 3270, 2973, 1685, 1161, 1107, 1038 cm^−1^; HRMS (*m*/*z*): [M + H]^+^ calcd for C_15_H_29_N_3_O_3_S_2_, 364.1723; found, 364.1725; [M + Na]^+^ calcd, 386.1543; found, 386.1544.

#### *(S*)-*tert*-Butyl 2-((3-((*S*)-*tert*-butylsulfinyl)thioureido)methyl)pyrrolidine-1-carboxylate ((*S*,*S*)-**5a**)


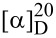
 +30.5 (*c* 0.5, MeOH); ^1^H NMR (600 MHz, CDCl_3_) δ 9.22 (s, 1H), 6.96 (s, 1H), 4.17–4.09 (m, 1H), 3.76–3.67 (m, 1H), 3.49–3.38 (m, 2H), 3.36–3.31 (m, 1H), 2.13–2.04 (m, 1H), 1.97–1.81 (m, 2H), 1.78–1.69 (m, 1H), 1.46 (s, 9H), 1.30 (s, 9H) ppm; ^13^C NMR (151 MHz, CDCl_3_) δ 182.3, 157.3, 80.6, 57.4, 55.9, 53.7, 47.5, 29.9, 28.5, 24.0, 22.2 ppm; IR (ATR): 3307, 2973, 1653, 1159, 1237, 1058 cm^−1^; HRMS (*m*/*z*): [M + Na]^+^ calcd for C_15_H_29_N_3_O_3_S_2_, 386.1543; found, 386.1543; [M + H]^+^ calcd, 364.1729; found, 364.1722.

#### General procedure for the preparation of *N*-sulfinylurea pre-catalysts (*S*,*R*)-**5b** and ((*S*,*S*)-**5b**)

A stirred solution of *(R)-tert-*butanesulfinamide or *(S)-tert-*butanesulfinamide (0.07 g, 0.6 mmol) in THF (5 mL) was cooled to −30 °C under argon atmosphere. Butyllithium in hexane (0.28 g, 0.66 mmol) was added dropwise and the solution was stirred for 15 min. The solution of (*S*)-*tert*-butyl 2-(isocyanatomethyl)pyrrolidine-1-carboxylate (**3b**, 0.15 g, 0.66 mmol) in dry THF (5 mL) was added dropwise during 15 min, the cooling bath was removed, and stirring was continued at rt for 22 h. The reaction was then quenched with water (0.3 mL) and the mixture was stirred for 30 min. The resulting mixture was concentrated and the desired product was isolated by column chromatography on silica gel (EtOAc/MeOH/NH_4_OH 60:1:0.6→50:1:0.5).

#### (*S*)-*tert*-Butyl 2-((3-((*R*)-*tert*-butylsulfinyl)ureido)methyl)pyrrolidine-1-carboxylate ((*S*,*R*)-**5b**)


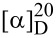
 −87.3 (*c* 0.5, MeOH); ^1^H NMR (600 MHz, CDCl_3_) δ 9.22 (s, 1H), 6.96 (s, 1H), 4.17–4.09 (m, 1H), 3.76–3.67 (m, 1H), 3.49–3.38 (m, 2H), 3.36–3.31 (m, 1H), 2.13–2.04 (m, 1H), 1.97–1.81 (m, 2H), 1.78–1.69 (m, 1H), 1.46 (s, 9H), 1.30 (s, 9H) ppm; ^13^C NMR (151 MHz, CDCl_3_) δ 182.3, 157.3, 80.6, 57.4, 55.9, 53.7, 47.5, 29.9, 28.5, 24.0, 22.2 ppm; IR (ATR): 3307, 2973, 1653, 1159, 1237, 1058 cm^−1^; HRMS (*m*/*z*): [M + H]^+^ calcd for C_15_H_29_N_3_O_4_S, 348.1957; found, 348.1952; [M + Na]^+^ calcd, 370.1776; found, 370.1771.

#### (*S*)-*tert*-Butyl 2-((3-((*S*)-*tert*-butylsulfinyl)ureido)methyl)pyrrolidine-1-carboxylate ((*S,S*)-**5b**)


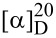
 +33.9 (*c* 0.5, MeOH); ^1^H NMR (600 MHz, CDCl_3_) δ 7.05 (s, 1H), 6.32 (s, 1H), 4.02–3.72 (m, 1H), 3.52–3.15 (m, 4H), 2.01–1.65 (m, 5H), 1.46 (s, 9H), 1.26 (s, 9H) ppm; ^13^C NMR (151 MHz, CDCl_3_) δ 156.7, 154.4, 79.6, 57.2, 56.1, 47.0, 45.1, 29.1, 28.5, 23.8, 22.3 ppm; IR (ATR): 3349, 2966, 1665, 1516, 1166, 1060 cm^−1^; HRMS (*m*/*z*): C_15_H_29_N_3_O_4_S, [M + H]^+^ calcd for C_15_H_29_N_3_O_4_S, 348.1957; found, 348.1950; [M + Na]^+^ calcd, 370.1776; found, 370.1769.

#### General procedure for the preparation of the catalysts **C1a**, **C1b**, **C2a**, **C2b**

The Boc-protected pre-catalyst **5a** or **5b** (0.1 mmol) was dissolved in cold dry CH_2_Cl_2_ (1 mL) and TFA (0.37 g, 3.3 mmol) was added. The reaction mixture was stirred at room temperature for 3 h. The solvent was removed in vacuo and the catalysts were obtained as their trifluoroacetate salts.

#### (*S*)-2-((3-((*R*)-*tert*-Butylsulfinyl)thioureido)methyl)pyrrolidin-1-ium 2,2,2-trifluoroacetate ((*S*,*R*)-**C1**)


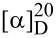
 −17.8 (*c* 1.0, MeOH); ^1^H NMR (300 MHz, CDCl_3_) δ 9.53 (s, 1H), 9.40 (s, 1H), 9.16 (s, 1H), 9.01 (s, 1H), 4.33–4.17 (m, 2H), 3.63–3.31 (m, 3H), 2.30–2.00 (m, 3H), 1.85–1.67 (m,1H), 1.33, 1.31 (s, 9H); ^13^C NMR (75 MHz, D_2_O) δ 184.6, 162.9 (q, *J* = 5.3 Hz), 116.3 (q, *J* = 291.7 Hz), 59.8, 56.9, 45.5, 38.7, 27.1, 22.6, 21.7 ppm; IR (ATR): 3231, 2981, 1672, 1578, 1362, 1199, 1128, 1016 cm^−1^; HRMS (*m*/*z*): [M − CF_3_COOH + H]^+^ calcd for C_12_H_22_F_3_N_3_O_3_S_2_, 264.1199; found, 264.1200; [M − CF_3_COOH + Na]^+^ calcd, 286.1018; found, 286.1019.

#### (*S*)-2-((3-((*S*)-*tert*-Butylsulfinyl)thioureido)methyl)pyrrolidin-1-ium 2,2,2-trifluoroacetate ((*S*,*S*)-**C1**)


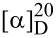
 +34.2 (*c* 1.0, MeOH); ^1^H NMR (600 MHz, D_2_O) δ 3.93–3.87 (m, 1H), 3.81–3.74 (m, 2H), 3.61–3.57 (m, 1H), 3.28–3.13 (m, 3H), 2.15–2.03 (m, 1H), 1.98–1.83 (m, 3H), 1.75–1.70 (m, 1H), 1.68–1.60 (m, 1H), 1.17 (s, 9H) ppm; ^13^C NMR (151 MHz, D_2_O) δ 184.5, 162.7 (q, *J* = 35.7 Hz), 117.1 (q, *J* = 286.9 Hz), 67.8, 59.9, 56.9, 45.5, 27.1, 22.5, 21.6 ppm; IR (ATR): 2969, 2721, 1660, 1551, 1316, 1153, 1044 cm^−1^; HRMS (*m*/*z*): [M − CF_3_COOH + H]^+^ calcd for C_12_H_22_F_3_N_3_O_3_S_2_, 264.1199; found, 264.1198; [M − CF_3_COOH + Na]^+^ calcd, 286.1018; found, 286.1016.

#### (*S*)-2-((3-((*R*)-*tert*-Butylsulfinyl)ureido)methyl)pyrrolidin-1-ium 2,2,2-trifluoroacetate ((*S*,*R*)-**C2**)


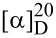
 −38.8 (*c* 1.0, MeOH); ^1^H NMR (300 MHz, D_2_O) δ 7.22 (bs, 1H), 7.05 (bs, 1H), 6.87 (bs, 1H), 3.72–3.08 (m, 5H), 2.12–1.87 (m, 3H), 1.63 (ddd, *J* = 17.2 Hz; 12.8 Hz; 8.6 Hz; 1H), 1.17 (s, 9H) ppm; ^13^C NMR (151 MHz, D_2_O) δ 162.9 (q, *J* = 17.3 Hz), 116.3 (q, *J* = 291.7 Hz), 60.4, 56,4, 45.5, 40.7, 26.8, 22.7, 21.4 ppm; IR (ATR): 3259, 2977, 1670, 1577, 1424, 1173, 1019 cm^−1^; HRMS (*m*/*z*): [M − CF_3_COOH + H]^+^ calcd for C_12_H_22_F_3_N_3_O_4_S, 248.1427; found, 248.1428.

#### (*S*)-2-((3-((*S*)-*tert*-Butylsulfinyl)ureido)methyl)pyrrolidin-1-ium 2,2,2-trifluoroacetate ((*S*,*S*)-**C2**)


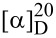
 +15.4 (*c* 0.25, MeOH); ^1^H NMR (600 MHz, D_2_O) δ 3.63–3.56 (m, 1H), 3.36 (dd, *J* = 15.2; 4.2 Hz, 1H), 3.26 (dd, *J* = 15.2, 7.6 Hz, 1H), 3.22–3.14 (m, 2H), 2.02 (dt, *J* = 12.6, 7.7 Hz, 1H), 1.98–1.84 (m, 2H), 1.61 (dq, *J* = 17.0, 8.6 Hz, 1H), 1.17, 1.16 (s, 9H) ppm; ^13^C NMR (151 MHz, D_2_O) δ 162.9 (q, *J* = 35.7 Hz), 161.0, 116.1 (q, *J* = 290.2 Hz), 60.7, 45.3, 40.6, 26.7, 22.9, 21.4, 18.1 ppm; IR (ATR): 3353, 2971, 1660, 1576, 1428, 1124, 1057 cm^−1^; HRMS (*m*/*z*): [M − CF_3_COOH + H]^+^ calcd for C_12_H_22_F_3_N_3_O_4_S, 248.1427; found, 248.1424.

#### Representative procedure for enantioselective Michael additions under solution conditions

The catalyst (0.015 mmol) and base (NMM, 2 mg, 0.015 mmol) were dissolved in the solvent (0.7 mL) and, after 10 min, the nitroalkene (0.5 mmol) in the solvent (0.7 mL) was added. After 10 min of stirring, the aldehyde (1.5 mmol) was added dropwise, and an acidic additive (0.015 mmol) was added. The resulting reaction mixture was stirred at room temperature for the appropriate reaction time. The reaction course was monitored by TLC. After completion of the reaction, the resulting mixture was concentrated in vacuo. The residue was diluted with water (10 mL). The layers were separated, and the aqueous layer was extracted with DCM (4 × 5 mL). The organic layer was then dried over Na_2_SO_4_ and concentrated. The desired products were isolated by flash column chromatography using silica gel as stationary phase.

#### Mechanochemical procedure for enantioselective Michael additions

The catalyst (0.016 mmol), base (0.016 mmol), nitroalkene (0.33 mmol), appropriate aldehyde (1 mmol), benzoic acid (0.03 mmol), and NaCl (1.2 g) were added to the ball mill reactor in one portion. The resulting mixture was mechanically activated for 3 h. The crude reaction mixture was dissolved in CH_2_Cl_2_ and NaCl was separated by simple filtration. The solvent was then evaporated under vacuum and the crude reaction mixtures were purified by column chromatography on silica gel.

#### Representative procedure for solvent-free enantioselective Michael additions

The catalyst (2.5–10 mol %), base (2.5–10 mol %), and half of volume of the aldehyde (total 5 equiv used) were added to a 10 mL vial vessel. After 5 min, the remaining volume of the aldehyde was added to the mixture. After further 5 min stirring, benzoic acid (10 mol %) was added and 10 min later, the nitroalkene (1 equiv) was added. The resulting reaction mixture was stirred at room temperature for the appropriate reaction time. The crude reaction mixture was purified by column chromatography using silica gel.

## Supporting Information

Supporting information contains characterization data for Michael adducts, pictures of NMR spectra, pictures of HPLC chromatograms, and DFT computational details.

File 1 Characterization data, copies of spectra, and DFT computational details
