# Supplementary material for: N-Sulfinylpyrrolidine-containing ureas and thioureas as bifunctional organocatalysts
Source: Beilstein J Org Chem. 2021 Oct 25;17:2629–41. doi: 10.3762/bjoc.17.176 (PMC8561142; doi:10.3762/bjoc.17.176)
Supplement: File 1 — Characterization data, copies of spectra, and DFT computational details [file Beilstein_J_Org_Chem-17-2629-s001.pdf]

BEILSTEIN JOURNAL OF ORGANIC CHEMISTRY

Supporting Information

for

N-Sulfinylpyrrolidine-containing ureas and thioureas as bifunctional organocatalysts

Viera Poláčková, Dominika Krištofíková, Boglárka Némethová, Renata Górová, Mária Mečiarová and Radovan Šebesta

Beilstein J. Org. Chem. **2021,** *17,* 2629–2641. doi:10.3762/bjoc.17.176

Characterization data, copies of spectra, and DFT computational details

License and Terms: This is a supporting information file under the terms of the Creative Commons Attribution License (https://creativecommons.org/licenses/by/4.0). Please note that the reuse, redistribution and reproduction in particular requires that the author(s) and source are credited and that individual graphics may be subject to special legal provisions.
The license is subject to the *Beilstein Journal of Organic Chemistry* terms and conditions: (https://www.beilstein-journals.org/bjoc/terms)

Table of contents

1. General considerations S2

2. Characterization data S3

3. Copies of ^{1}H, ^{13}C NMR and HRMS spectra S7

4. Copies of HPLC records S28

5. Computational details S39

6. References S77

1. General considerations

All commercially available chemicals were purchased from Merck, Alfa Aesar or TCI and were used without further purification. Solvents were distilled and dried according to the standard procedures. Thin-layer chromatography was performed on silica gel 60, F-254 nm plates. Compounds were visualized by irradiation with UV light and/or by treatment with $KMnO_4$ or *p*-anisaldehyde solution. NMR spectra were recorded on Varian NMR System 300 and Varian NMR System 600 (300 or 600 MHz for ^{1}H, 75 or 151 MHz for ^{13}C). Chemical shifts (δ ppm) are given in ppm relative to tetramethylsilane. Products of Michael additions were purified by flash chromatography using Isolera Biotage FSKO-1107-0010. Enantiomeric purity was determined by chiral HPLC column (Chiralcel AS-H, Chiralpak IC Chiralcel ODH). Specific optical rotations were measured on Jasco P-2000 polarimeter. High-resolution mass spectra were recorded with Thermo Velos Pro Orbitrap spectrometer. Melting points were measured on Büchi Melting Point M-565. Infrared spectroscopy spectra were recorded with Agilent Technologies Cary 630 FTIR. Frequencies are given in wavenumbers (cm^{-1}) and only selected peaks were reported. Mechanochemical reactions were carried out in a ball mill Retsch MM 400.

2. Characterization data

2-Ethyl-4-nitro-3-phenylbutanal (**8a**)[1]

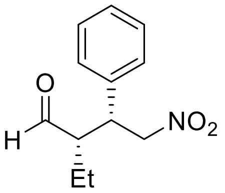

Colourless oil, RF = 0.44 (Hexanes/EtOAc, 5:1), **^{1}H NMR** (600 MHz, $CDCl_3$) δ 9.72 (d, *J* = 2.6 Hz, 0.84H); 9.49 (d, *J* = 2.9 Hz, 0.16H); 7.36–7.28 (m, 3H); 7.19–7.17 (m, 2H); 4.83–4.75 (m, 0.32H); 4.72 (dd, *J* = 12.7; 4.9 Hz, 0.84H); 4.63 (dd, *J* = 12.7; 9.7 Hz, 0.84H); 3.79 (td, *J*= 9.8; 4.9 Hz, 1H); 2.7–2.66 (m, 1H); 1.54–1.49 (m, 2H); 0.99 (t, *J* = 7.5 Hz, 0.5H); 0.84 (t, *J* = 7.5 Hz, 2.5H) ppm. **HPLC:** Chiralcel ODH, Hexane/iPrOH 80:20, 0.8 ml/min, λ = 211 nm, t_R = 18.4 min and 32.3 min (minor), t_R = 17.1 min and 21.0 min (major).

2-Ethyl-3-(4-methoxyphenyl)-4-nitrobutanal (**8b**)[2]

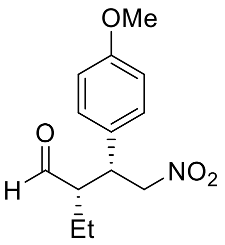

Colourless oil, RF = 0.35 (Hexanes/EtOAc, 4:1), **^{1}H NMR** (600 MHz, $CDCl_3$) δ 9.71 (d, *J* = 2.6 Hz, 0.84 H); 9.47 (d, *J* = 3 Hz, 0.16 H); 7.09 (d, *J* = 8.6 Hz, 2H); 6.87 (d, *J* = 8.7 Hz, 2H); 4.8–4.71 (m, 0.32H); 4.69 (dd, *J* = 12.6; 4.9 Hz, 0.84H); 4.58 (dd, *J* = 12.5; 9.9 Hz, 0.84H); 3.79 (s, 2.5H); 3.78 (s, 0.5H); 3.74 (td, *J* = 9.9; 4.9 Hz, 1H); 2.65–2.61 (m, 1H); 1.55–1.47 (m, 2H); 0.99 (t, *J* = 7.5 Hz, 0.5H); 0.83 (t, *J* = 7.5 Hz, 2.5H) ppm. **HPLC:** Chiralpak IC, Hexane/iPrOH 80:20, 1 ml/min, λ = 210 nm, t_R = 18.2 min and 23.8 min (minor), t_R = 28.7 min and 30.8 min (major).

2-[1-(4-Methoxyphenyl)-2-nitroethyl]hexanal (**8c**)[3]

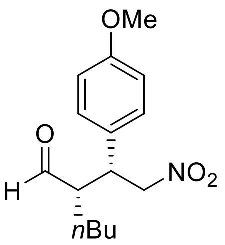

Colourless oil, RF = 0.36 (Hexanes/EtOAc, 4:1), **^{1}H NMR** (300 MHz, $CDCl_3$) δ 9.70 (d, *J* = 2.8 Hz, 0.63H); 9.46 (d, *J* = 3.1 Hz, 0.37H); 7.08 (d, *J* = 8.7 Hz, 2H); 6.87 (d, *J* = 8.7 Hz, 1H); 6.85 (d, *J* = 8.7 Hz, 1H); 4.78 (dd, *J* = 12.8; 6.0 Hz, 0.37H); 4.71 (dd, *J* = 12.8; 9.4 Hz, 0.37H); 4.67 (dd, *J* = 12.6; 5.0 Hz, 0.63H); 4.59 (dd, *J* = 12.6; 9.8 Hz, 0.63H); 3.79 (s, 1.1H); 3.78 (s, 1.9H); 3.74–3.70 (m, 1H); 2.67–2.62 (m, 0.63H); 2.60–2.56 (m, 0.37H); 1.52–1.37 (m, 2.4H); 1.26–1.12 (m, 3.6H); 0.89 (m,1.1H); 0.79 (t, *J* = 6.9 Hz, 1.9H) ppm. **^{13}C NMR** (151 MHz, $CDCl_3$) δ 203.5; 203.4; 159.3; 159.2; 129.3; 129.0; 128.5; 127.9; 114.5; 114.4; 78.7; 78.2; 55.22; 55.21; 54.0; 53.5; 43.8; 42.5; 29.1; 28.5; 27.3; 27.0; 22.6; 22.5; 13.8; 13.7 ppm. **HRMS (ESI):** $C_{14}H_{21}NO_4$ calculated $[M+Na]^+$ = 302.1363, found 302.1362. **IR (ATR):** 2930.2; 2859.8;1719.0; 1610.9; 1549.3; 1512.7; 1464.0; 1377.8; 1249.5; 1179.6; 1032.2; 830.5;

755.6 cm^{-1}. **HPLC:** Chiralpak IC, Hexane/iPrOH 80:20, 1 ml/min, λ = 210 nm, t_R = 15.0 min and 20.8 min (minor), t_R = 22.2 min and 24.9 min (major).

2-[1-(4-Fluorophenyl)-2-nitroethyl]hexanal (**8d**)[3]

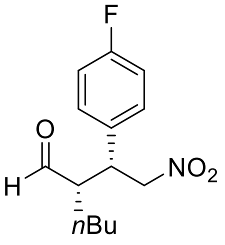

Colourless oil, RF = 0.36 (Hexanes/EtOAc, 4:1), **^{1}H NMR** (600 MHz, $CDCl_3$) δ 9.71 (d, *J* = 2.6 Hz, 0.8H); 9.48 (d, *J* = 2.8 Hz, 0.2H); 7.17−7.15 (m, 2H); 7.06–7.02 (m, 2H); 4.80 (dd, *J* = 12.9; 5.8 Hz, 0.2H); 4.75–4.71 (m, 0.2H); 4.70 (dd, *J* = 12.8; 4.8 Hz, 0.8H); 4,61 (dd, *J* = 12.8; 9.9 Hz, 0.8H); 3.77(td, *J* = 9.8; 4.8 Hz, 1H); 2.70−2.65 (m, 0.8H); 2.64−2.60 (m, 0.2H); 1.49−1.45 (m, 1H); 1.43–1.38 (m, 1H); 1.26–1.22 (m, 2H); 1.18–1.13 (m, 2H); 0.90–0.89 (m, 0.6H), 0.79 (t, *J* = 6.9 Hz, 2.4H) ppm. **^{13}C NMR** (151 MHz, $CDCl_3$) δ 203.0; 202.9; 162.3 (d, *J* = 247.4 Hz); 132.5 (d, *J* = 3.4 Hz); 129.9 (d, *J* = 8.1 Hz); 129.6 (d, *J* = 8.2 Hz); 116.2 (d, *J* = 21.5 Hz); 116.1 (d, *J* = 21.5 Hz); 78.4; 77.9; 53.8; 53.5; 43.6; 42.4; 29.1; 28.4; 27.2; 27.0; 22.6; 22.5; 13.7; 13.6 ppm. **HRMS (ESI):** $C_{14}H_{18}FNO_3$ calculated $[M+H]^+$ = 268.1343, found 268.1352. **IR (ATR):** 2956.5; 2927.8; 2860.3; 1685.7 1636.9; 1458.2; 1413.5; 1378.4 1274.1; 1213.2; 933.4; 720.0; 659.7 cm^{-1}. **HPLC:** Chiralpak IC, Hexane/iPrOH 95:5, 1 ml/min, λ= 210 nm, t_R = 18.7 min and 28.6 min (minor), t_R = 30.1 min: and 35.4 min (major).

2-Benzyl-3-(4-methoxyphenyl)-4-nitrobutanal (**8e**)

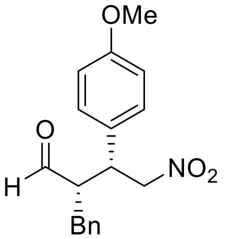

Colourless oil, RF = 0.35 (Hexanes/EtOAc, 3:1), **^{1}H NMR** (600 MHz, $CDCl_3$) δ 9.71 (d, *J* = 2.3 Hz, 0.8H); 9.55 (d, *J* = 1.9 Hz, 0.2H); 7.32–7.24 (m, 2H); 7.21–7.19 (m, 1H); 7.13 (d, *J* = 8.6 Hz, 2H); 7.09 (d, *J* = 8.7 Hz, 0.4H); 7.03 (d, *J* = 7.2 Hz, 1.6H); 6.9 (d, *J* = 8.7 Hz, 1.6H); 6.87 (d, *J* = 8.7 Hz, 0.4H); 4.84 (dd, *J* = 13.0, 6.3 Hz, 0.2 H), 4.75 (dd, *J* = 12.9, 9.1 Hz, 0,2 H), 4.70 (dd, *J* = 12.7, 5.4 Hz, 0,8 H), 4.65 (dd, *J* = 12.6, 9.3 Hz, 0,8 H); 3.81 (s, 2.4H); 3.80 (s, 0.6H); 3.79–3.75 (m, 1H); 3.09–2.99 (m, 1H); 2.76 (d, *J* = 7.3 Hz, 2H) ppm. **^{13}C NMR** (151 MHz, $CDCl_3$) δ 202.2; 202.1; 158.4; 136.3; 136.2; 128.1; 127.9; 127.85; 127.8; 127.7; 127.35; 127.3; 126.0; 125.9; 113.6; 113.5; 77.3; 76.9; 54.5; 54.2; 53.4; 42.8; 41.8; 33.2; 32.7 ppm. **HRMS (ESI):** $C_{18}H_{19}NO_4$ calculated $[M-H]^-$ = 312.1241, found 312.1245. **IR (ATR):** 3026.6; 2925.8; 2836.5; 1719.6; 1610.0; 1548.6; 1512.4; 1540.0; 1438.1; 1377.7; 1249.5; 1179.3; 1029.8; 830.1; 749.4 cm^{-1}. **HPLC:** Chiralpak IC, Hexane/iPrOH 80:20, 1 ml/min, λ = 210 nm, t_R = 15.6 min and 20.8 min (minor), t_R =19.9 min and 26.1 min (major).

2-Benzyl-3-(4-fluorophenyl)-4-nitrobutanal (**8f**)[3]

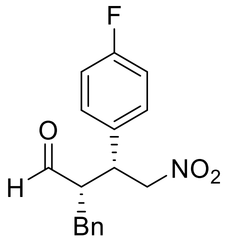

Colourless oil, RF = 0.34 (Hexanes/EtOAc, 3:1), **^{1}H NMR** (600 MHz, $CDCl_3$) δ 9.72 (d, *J* = 2.1 Hz, 0.62H); 9.55 (d, *J* = 2.0 Hz, 0.38H); 7.23–7.18 (m, 5H); 7.08–7.04 (m, 2H); 7.02–7.01 (m, 2H); 4.85 (dd, *J* = 13.1; 6.2 Hz, 0.38H); 4.78 (dd, *J* = 13.1; 9.2 Hz, 0.38H); 4.72 (dd, *J* = 12.8; 5.2 Hz, 0.62H); 4.68 (dd, *J*= 12.8; 9.4 Hz, 0.62H); 3.82 (td, *J* = 9.2; 5.2 Hz, 1H); 3.12–3.07 (m,1H); 2.8–2.73 (m, 2H) ppm. **^{13}C NMR** (151 MHz, $CDCl_3$) δ 202.8; 202.7; 162.42 (d, *J* = 247.8 Hz); 162.4 (d, *J* = 247.9 Hz); 137.0; 136.9; 132.5 (d, *J* = 3.4 Hz); 131.6 (d, *J* = 3.4 Hz); 130.2 (d, *J* = 8.1 Hz); 129.7 (d, *J* = 8.2 Hz); 128.9; 128.8; 128.7; 127.1; 127.0; 116.3 (d, *J* = 21.5 Hz); 116.1 (d, *J* = 21.5 Hz) 78.0; 77.7; 55.2; 54.3; 43.7; 42.7; 34.2 33.7 ppm. **HRMS (ESI):** $C_{17}H_{16}FNO_3$ calculated $[M+H]^+$ = 302.1187, found 302.1192. **IR (ATR):** 3026.2; 2921.7; 1720.8; 1602.6; 1553.7; 1509.8; 1453.8; 1377.5; 1225.3; 1161.0; 835.4; 746.5; 699.6 cm^{-1}. **HPLC:** Chiralpak IC, Hexane/iPrOH 80:20, 1 ml/min, λ = 210 nm, t_R = 10.2 min and 14.7 min (minor), t_R =13.3 and 16.5 (major).

2-Benzyl-3-(furan-2-yl)-4-nitrobutanal (**10a**)[4]

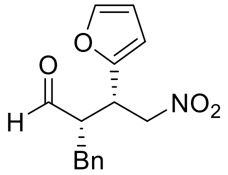

Pale yellow oil, R_F = 0.48 (Hexanes/EtOAc, 3:1), **^{1}H NMR** (600 MHz, $CDCl_3$) δ 9.71 (d, *J* = 1.1 Hz, 0.75H); 9.69 (d, *J* = 1.9 Hz, 0.25H); 7.40 (s, 1H); 7.34–7.27 (m, 2H); 7.25–7.22 (m, 1H); 7.15 (d, *J* = 7.4 Hz, 0.5H); 7.12 (d, *J* = 7.4 Hz, 1.5H); 6.36–6.33 (m, 1H); 6.24–6.21 (m, 1H), 4.76–4.60 (m, 2H); 4.10–4.05 (m, 0.75H); 3.98–3.93 (m, 0.25H); 3.20–3.14 (m, 0.75H); 3.05 (dd, *J* = 13.9; 7.9 Hz, 0.25H); 3.00–2.95 (m, 0.25H); 2.88 (dd, *J* = 14.4; 8.9 Hz, 0.75H); 2.81 (dd, *J* = 14.0; 7.1 Hz, 0.25H); 2.76 (dd, *J* = 14.4; 5.6 Hz, 0.75H) ppm. **HPLC:** Chiralcel AS-H, heptane/iPrOH 90:10, 0.7 mL/min, λ = 220 nm, t_R = 29.2 min, t_R = 39.5 min.

3-(Furan-2-yl)-2-methyl-4-nitrobutanal (**10b**)[5]

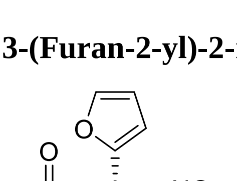

Brown oil, RF = 0.32 (Hexanes/EtOAc, 3:1), **^{1}H NMR** (600 MHz, $CDCl_3$) δ 9.71 (d, *J* = 0.8 Hz, 0.59H); 9.64 (d, *J* = 1.3 Hz, 0.41H); 7.37–7.36 (m, 1H); 6.32–6.3 (m, 1H); 6.2 (dd, *J* = 12.6; 3.3 Hz, 1H); 4.77–4.68 (m, 2H); 4.09 (dt, *J* = 8.5; 6.7 Hz, 0.59H); 4.01 (dt, *J* = 8.8; 5.6 Hz, 0.41H); 2.87–2.78 (m, 1H); 1.22 (d, *J* = 7.3 Hz, 1.75H); 1.08 (d, *J* = 7.3 Hz, 1.25H) ppm. **HPLC:** Chiralcel AS-H, Hexane/iPrOH 90:10, 0.8 ml/min, λ = 210 nm, t_R = 23.1 min and 27.8 min (minor), t_R = 20.0 min and 21.4 min (major).

2-[1-(Furan-2-yl)-2-nitroethyl]hexanal (**10c**)[3]

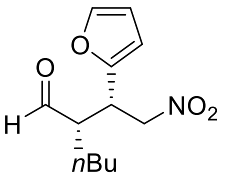

Brown oil, RF = 0.47 (CH_2Cl_2/ Hexanes, 3:1), **^{1}H NMR** (300 MHz, $CDCl_3$) δ 9.71 (d, *J* = 1.9 Hz, 0.5H); 9.6 (d, *J* = 2.7 Hz, 0.5H); 7.37–7.36 (m, 1H); 6.32–6.30 (m, 1H); 6.21–6.19 (m, 1H); 4.78–4.62 (m, 2H); 4.04–3.93 (m, 1H); 2.78 (tdd, *J* = 8.1; 4.6; 1.9 Hz, 0.5H); 2.60 (dtd, *J* = 8.3; 5.4; 2.7 Hz, 0.5H); 1.74–1.64 (m, 1H); 1.55–1.44 (m, 2H); 1.35–1.28 (m, 3H); 0.89–0.85 (m, 3H) ppm. **^{13}C NMR** (151 MHz, $CDCl_3$) δ 202.3; 202.8; 150.1; 149.6; 142.7; 142.6; 110.5; 110.4; 108.9; 108.7; 76.1; 75.9; 52.2; 52.1; 38.0; 36.9; 29.2; 28.7; 26.9; 26.5; 22.6; 22.5; 13.7; 13.6 ppm. **HRMS (ESI):** $C_{12}H_{17}NO_4$ calculated $[M+H]^+$ = 240.1230, found 240.1229. **IR (ATR):** 2930.5; 2861.5; 2729.0; 1719.4; 1551.0; 1505.7; 1430.9; 1376.0; 1190.8; 1148.0; 1072.9; 1013.6; 913.7; 735.2 cm^{-1}. **HPLC:** Chiralpak IC, Hexane/iPrOH 95:5, 0.6 ml/min, λ = 217 nm, t_R = 50.1 min and 60.2 min (minor), t_R = 44.8 min and 57.3 (major).

2-Benzyl-4-nitro-3-(pyridin-3-yl)butanal (**12**)[6]

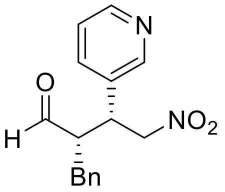

Pale yellov oil, R_F = 0.21 (Hexanes/EtOAc, 1:1), $[\boldsymbol{\alpha}]_{\boldsymbol{D}}^{\boldsymbol{20}}$ = 3.58 (*c* = 0.5, MeCN), **^{1}H NMR** (600 MHz, $CDCl_3$) δ 9.71 (d, *J* = 1.6 Hz, 0.6H); 9.57 (d, *J* = 1.1 Hz, 0.4H), 8.58–8.53 (m, 1H); 8.48 (dd, *J* = 9.8 Hz; 1.9 Hz, 1H); 7.59–7.54 (m, 1H); 7.35–7.18 (m, 4H); 7.12 (d, *J* = 7.3 Hz, 1H); 7.01 (d, *J* = 7.2 Hz, 1H); 4.91–4.83 (m, 1H); 4.76 (d, *J* = 6.8 Hz, 1H); 3.88–3.82 (m, 1H); 3.21–3.13 (m, 1H); 3.0 (dd, *J* = 19.5; 11.3 Hz, 0.6H); 2.85–2.74 (m, 2H) ppm. **^{13}C NMR** (151 MHz, $CDCl_3$) δ 202.3, 202.2, 150.0, 149.6, 149.51, 149.5, 136.5, 136.4, 136.1, 135.7, 132.8, 132,1, 129.1, 129.0, 128.73, 128.5, 128.3, 127.3, 127.2, 124.0, 123.9, 54.7, 54.1, 53.4, 41.6, 40.9, 34.2, 33.5 ppm. **IR (ATR):** 1721.0; 1550.5; 1377.0; 1495.9 cm^{-1}. **HRMS (ESI):** $C_{16}H_{15}N_2O_3$ calculated $[M-H]^-$ = 283.1077, found 283.1088. **HPLC:** Chiralcel AS-H, hexane/iPrOH, 70:30, 0.75 mL/min, λ = 218 nm, t_R = 27.0 min and 29.2 (major), t_R =34.4 and 43.4 min (minor).

3. Copies of ^{1}H, ^{13}C NMR and HRMS spectra

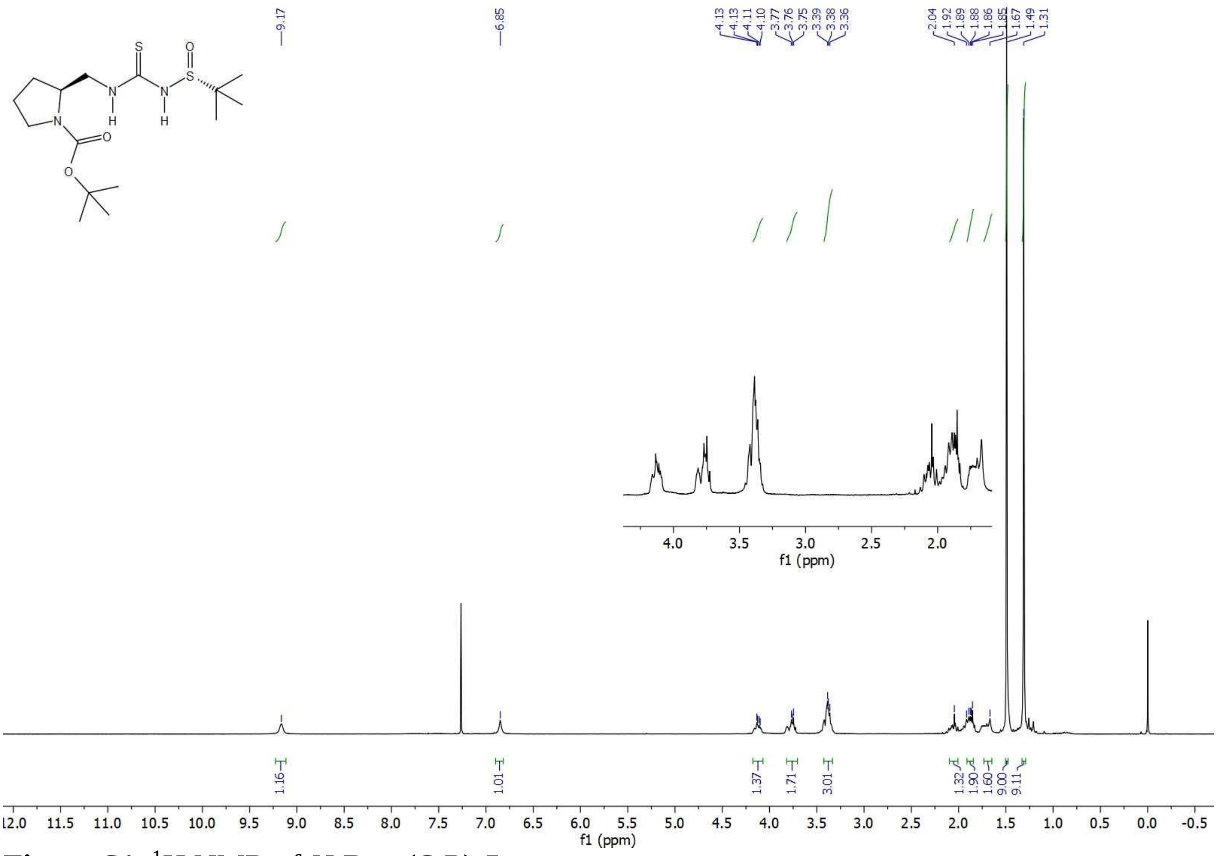

Figure S1. ^{1}H NMR of ***N*-Boc-(*S*,*R*)-5a**

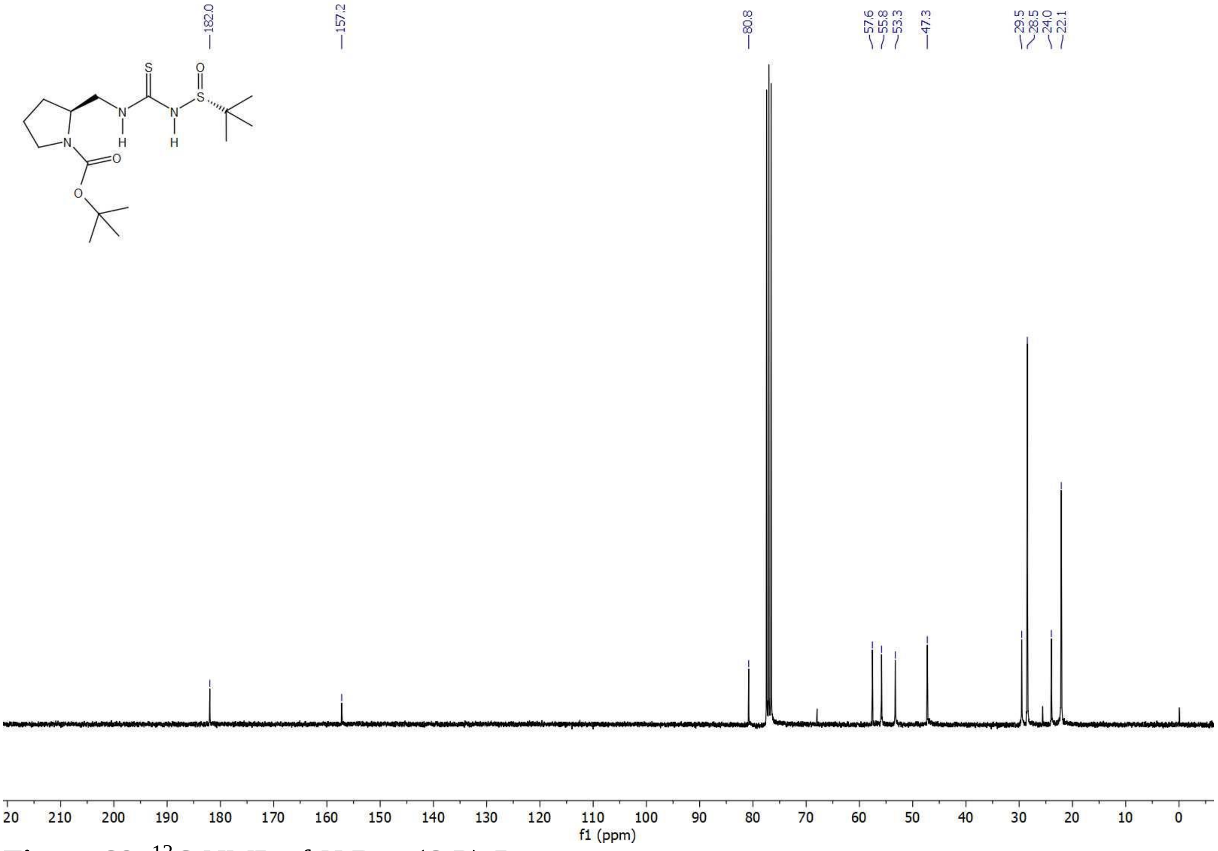

Figure S2. ^{13}C NMR of ***N*-Boc-(*S*,*R*)-5a**

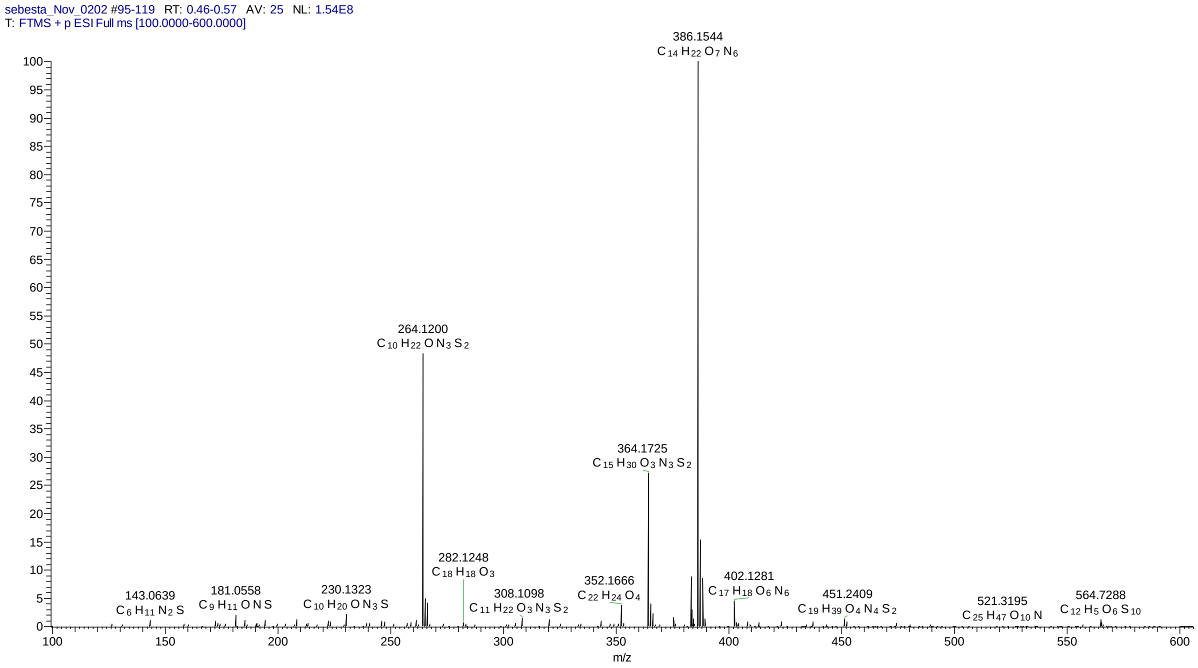

Figure S3. HRMS of ***N*-Boc-(*S*,*R*)-5a**

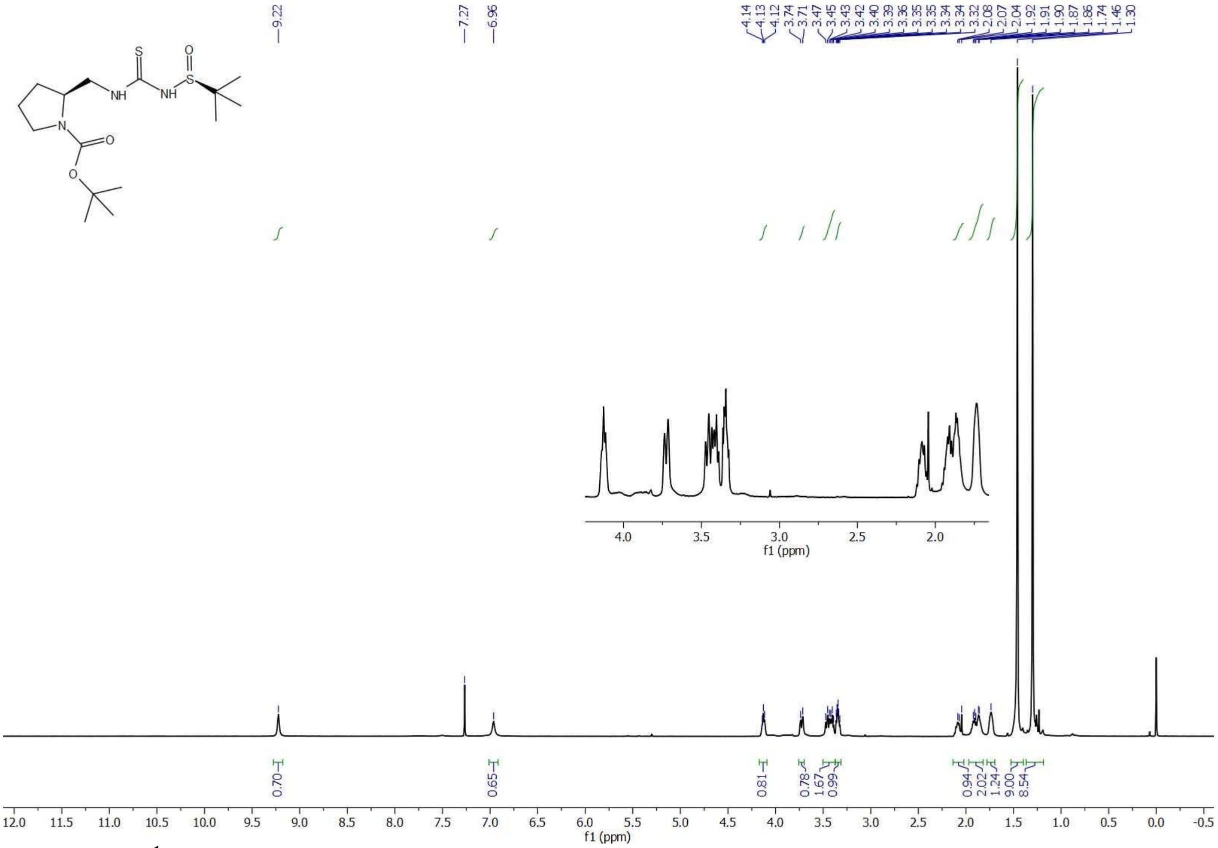

Figure S4. ^{1}H NMR of ***N*-Boc-(*S*,*S*)-5a**

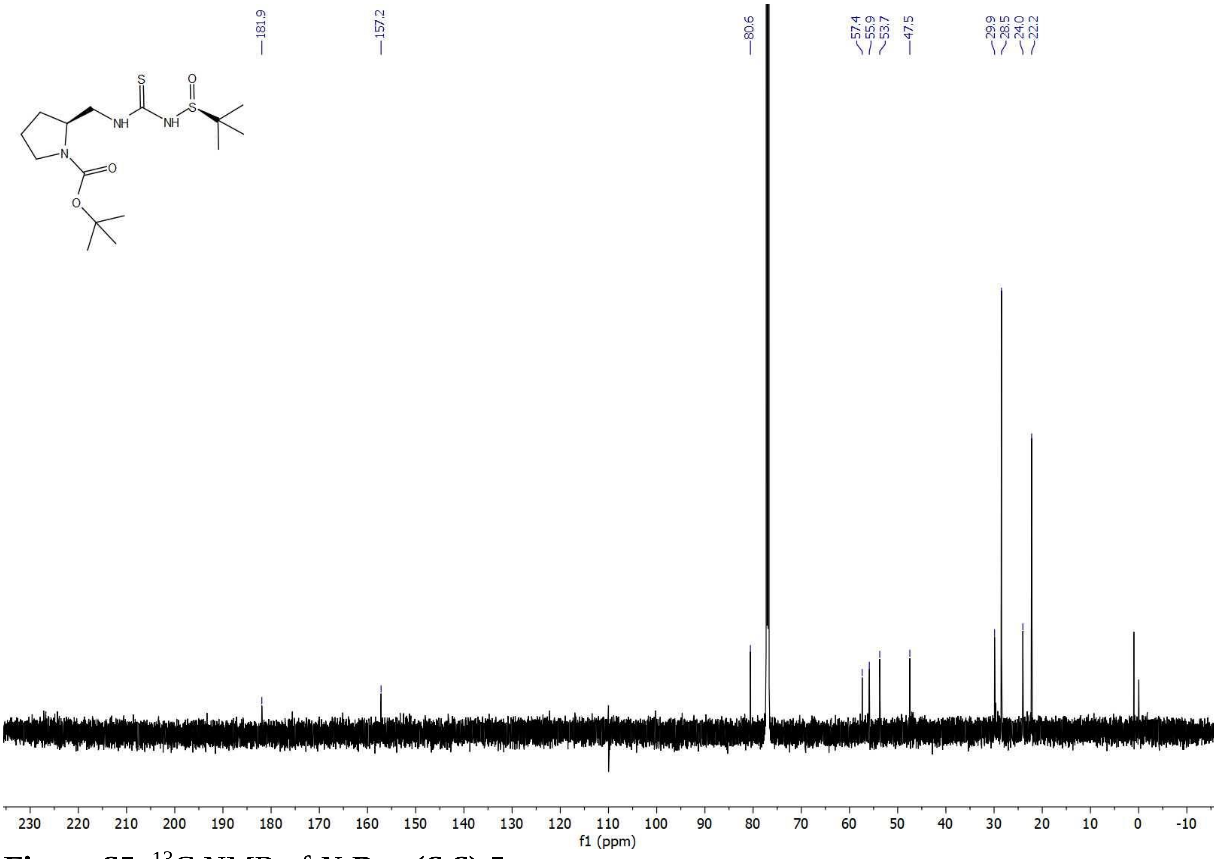

Figure S5. ^{13}C NMR of ***N*-Boc-(*S*,*S*)-5a**

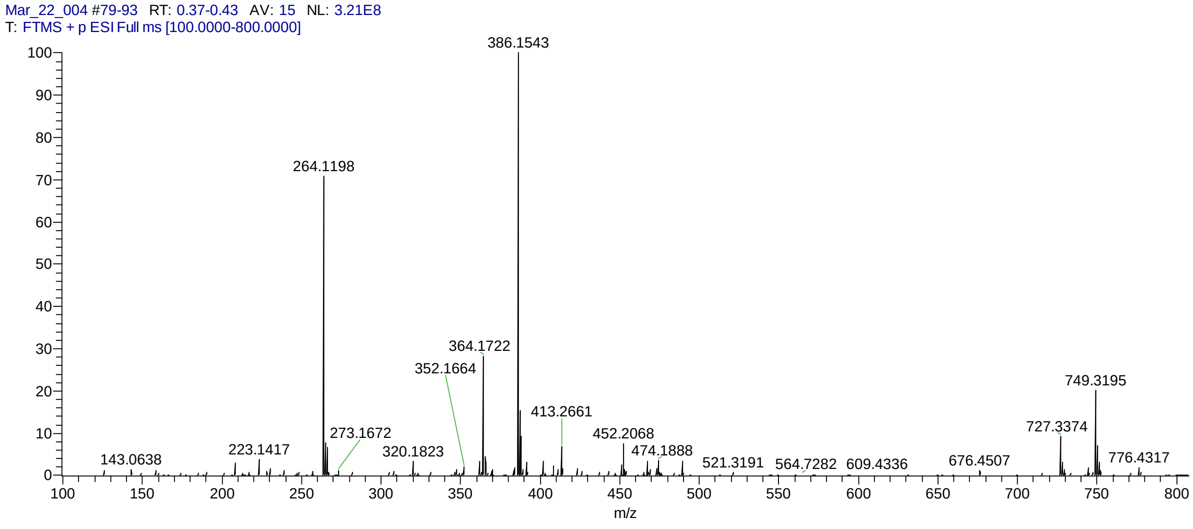

Figure S6. HRMS of ***N*-Boc-(*S*,*S*)-5a**

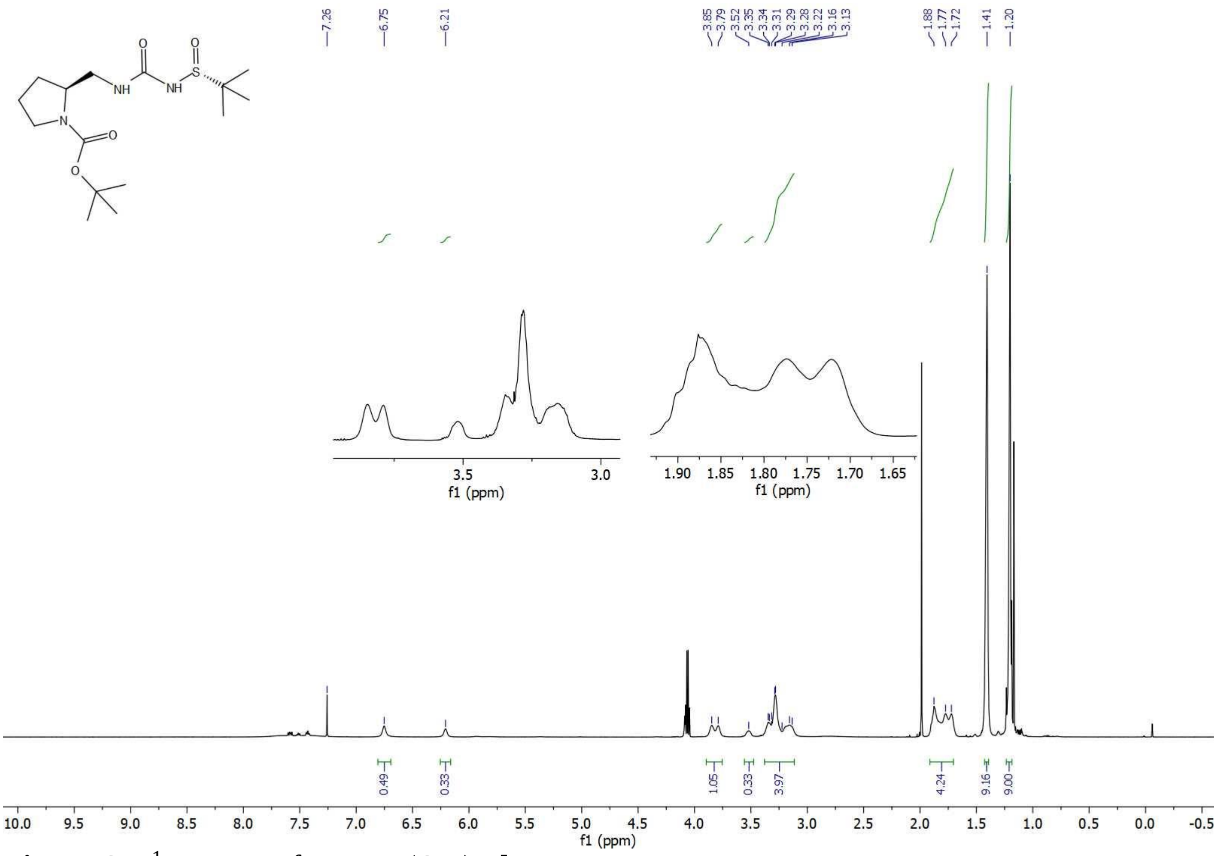

Figure S7. ^{1}H NMR of ***N*-Boc-(*S*,*R*)-5b**

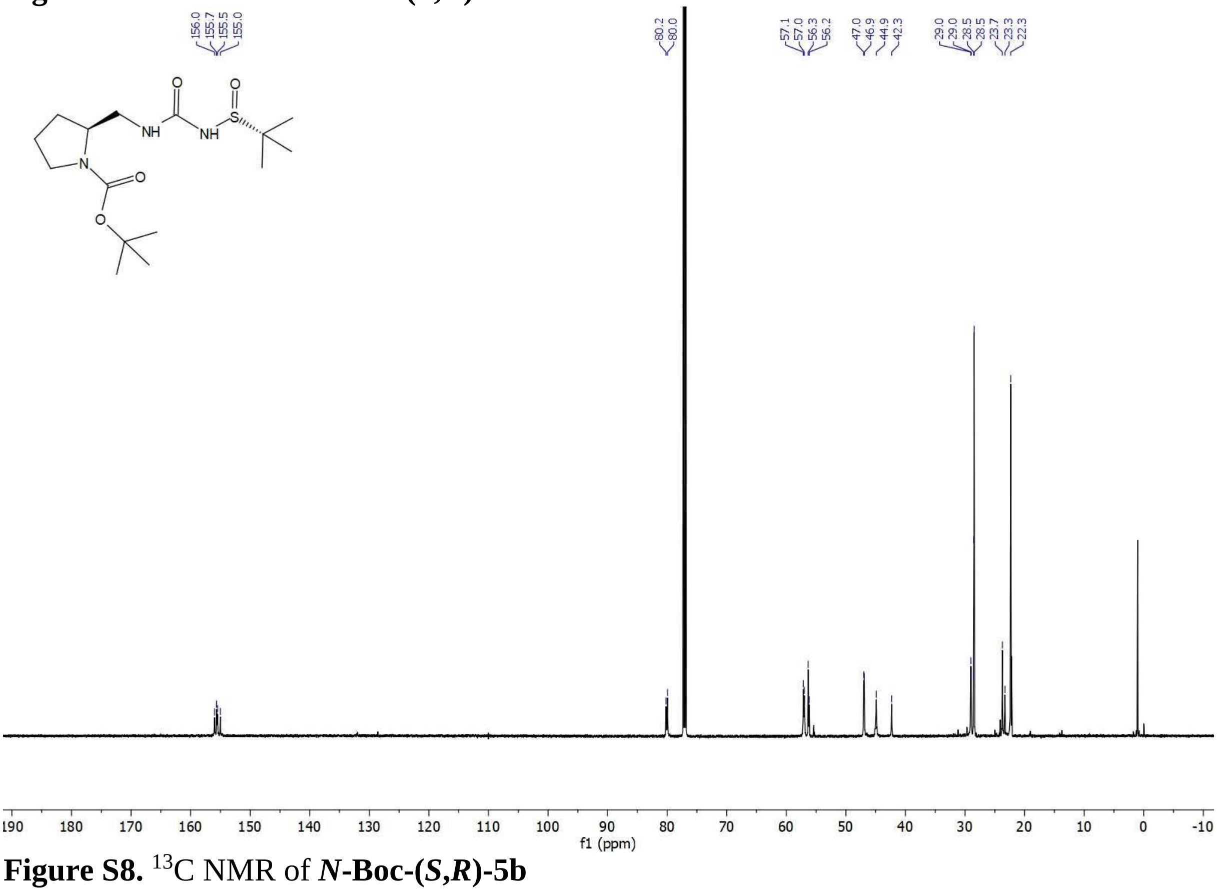

Figure S8. ^{13}C NMR of ***N*-Boc-(*S*,*R*)-5b**

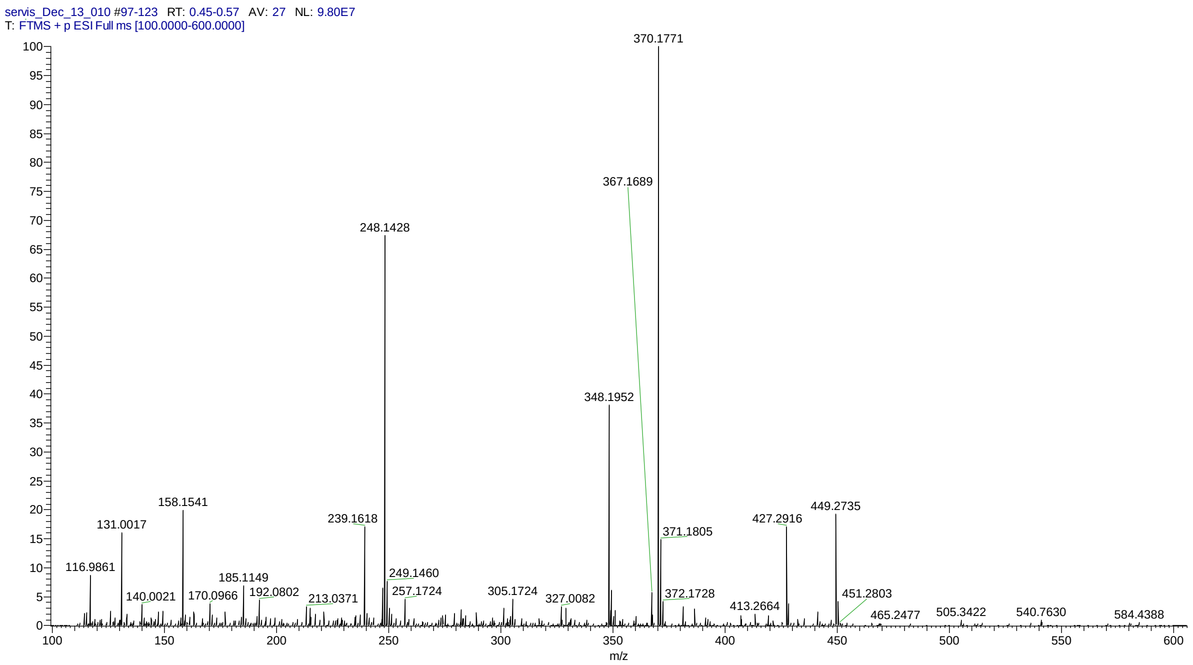

Figure S9. HRMS of ***N*-Boc-(*S*,*R*)-5b**

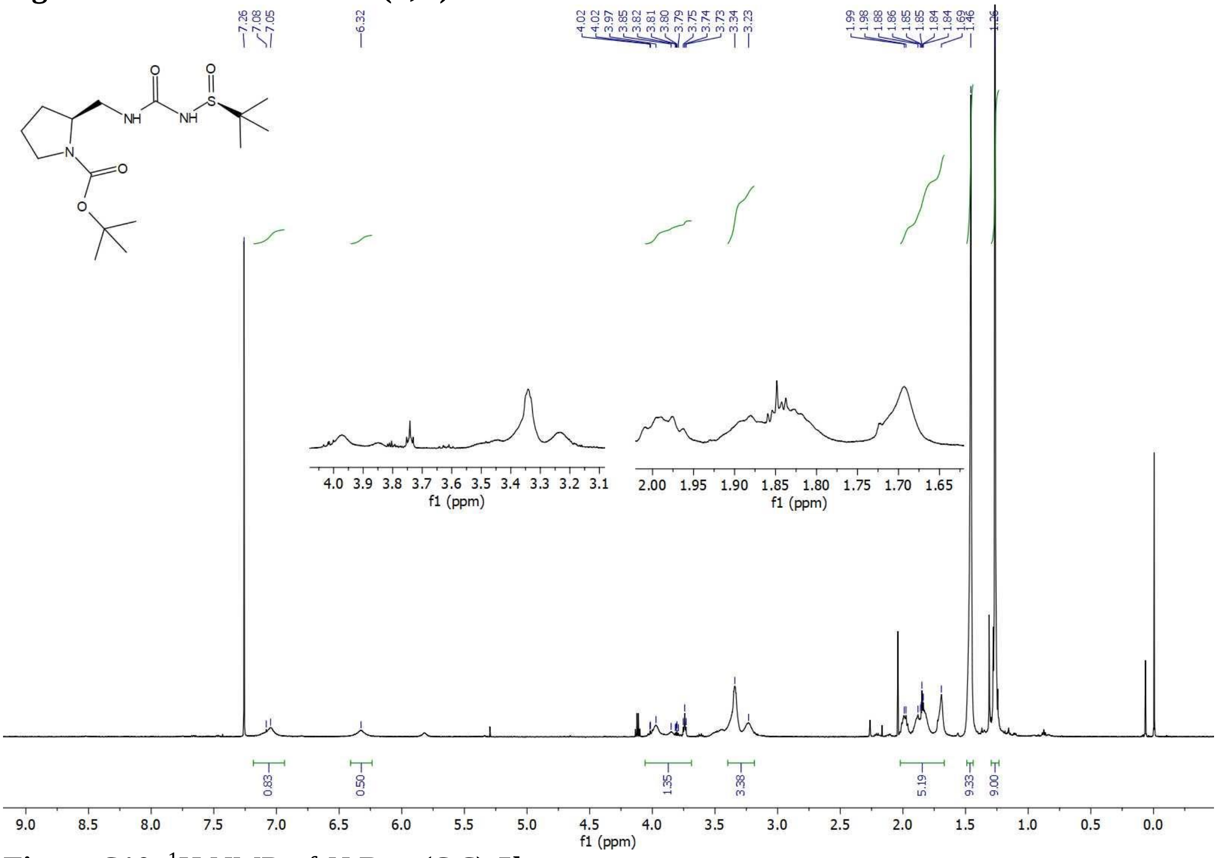

Figure S10. ^{1}H NMR of ***N*-Boc-(*S*,*S*)-5b**

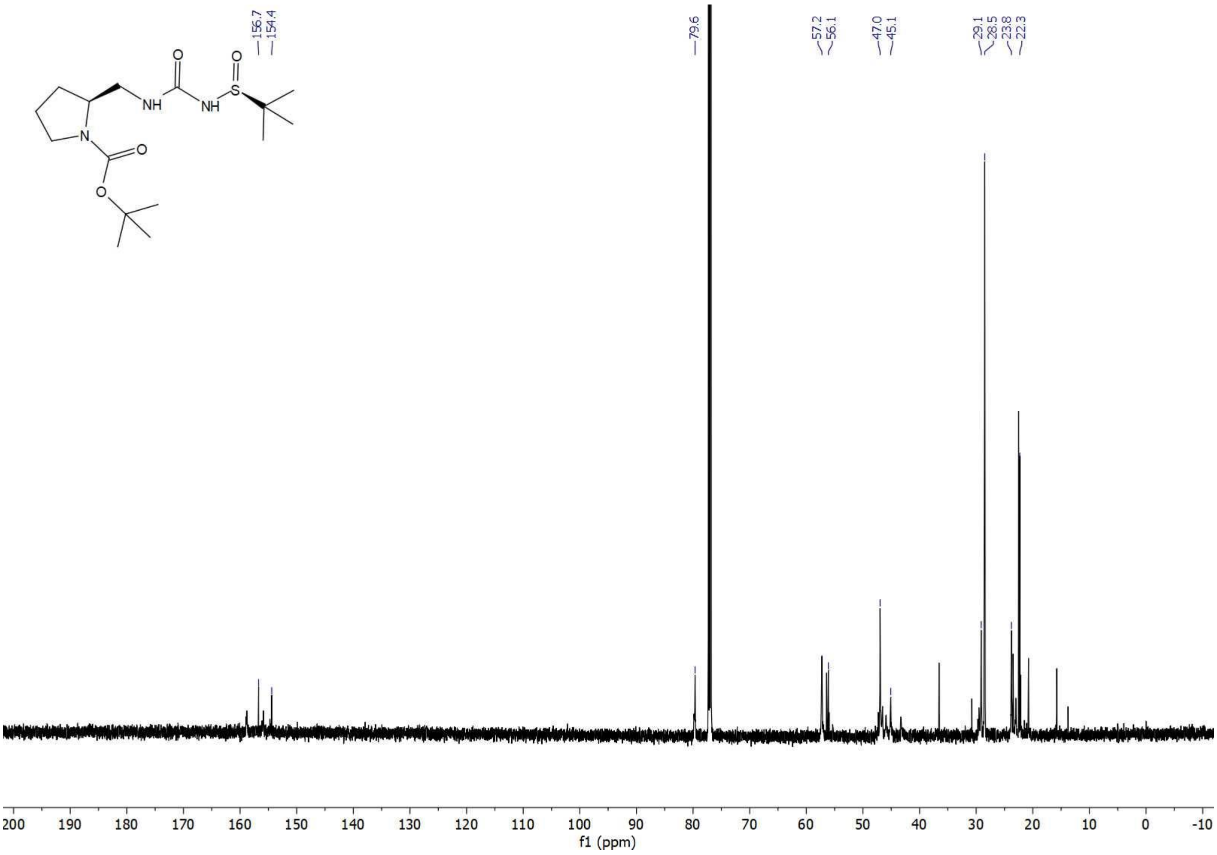

Figure S11. ^{13}C NMR of ***N*-Boc-(*S*,*S*)-5b**

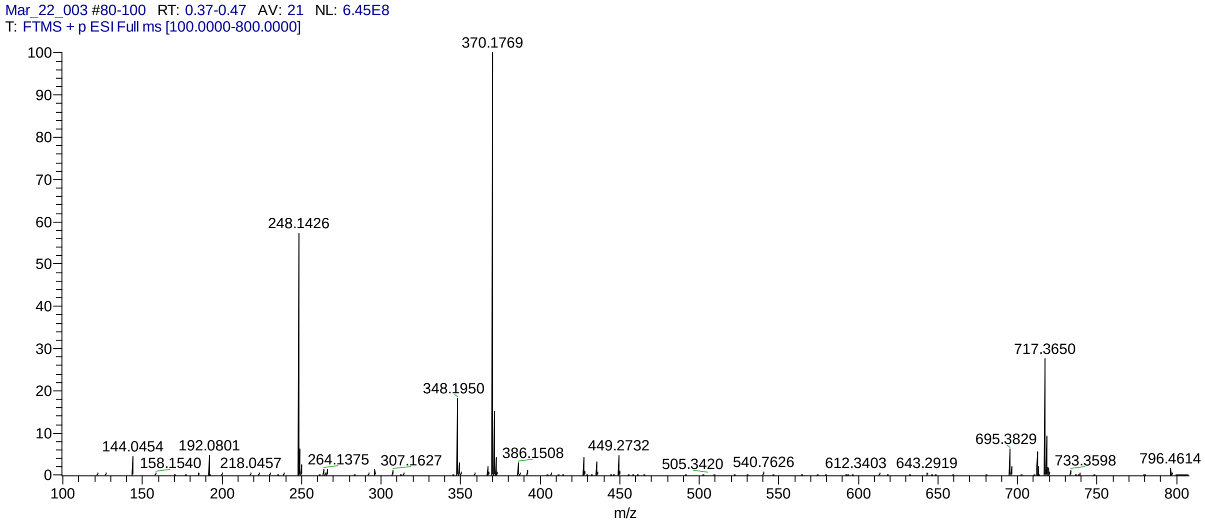

Figure S12. HRMS of ***N*-Boc-(*S*,*S*)-5b**

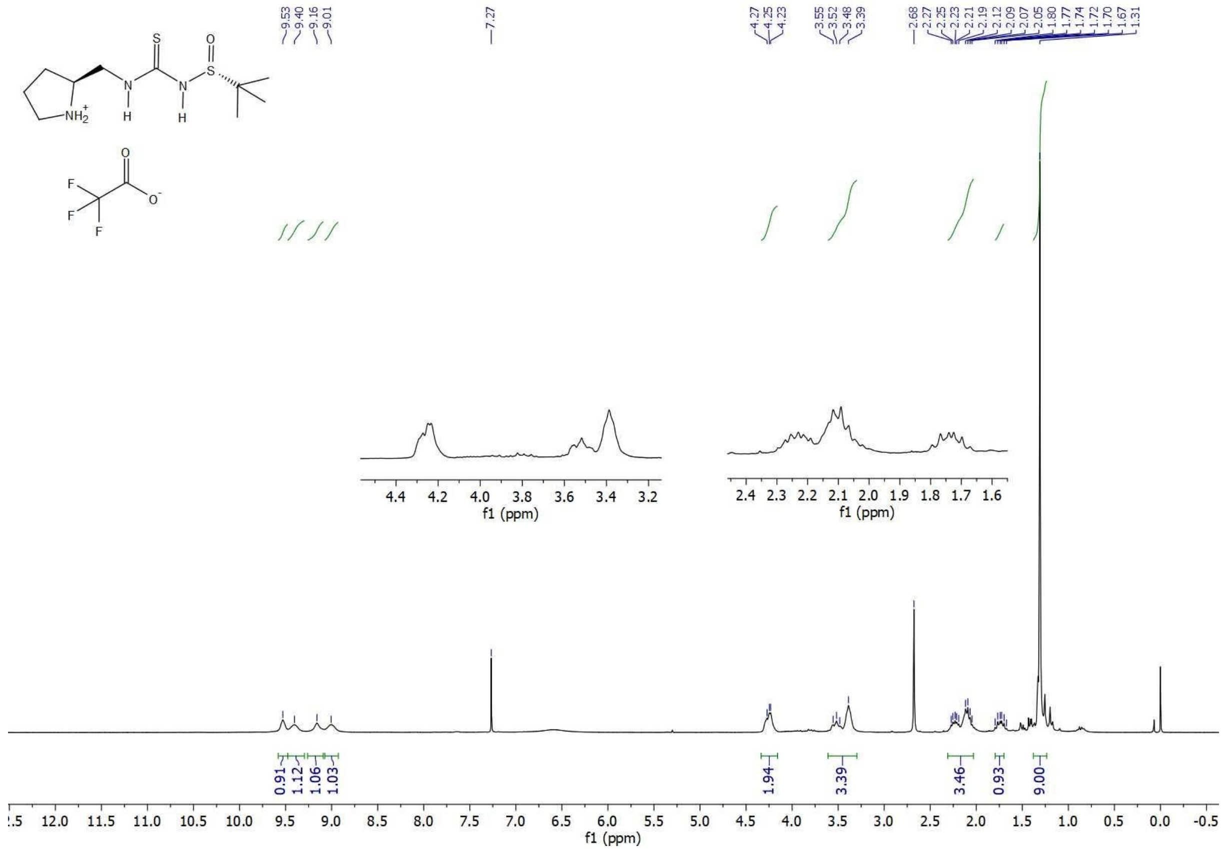

Figure S13. ^{1}H NMR of ***(S,R)*-C1**

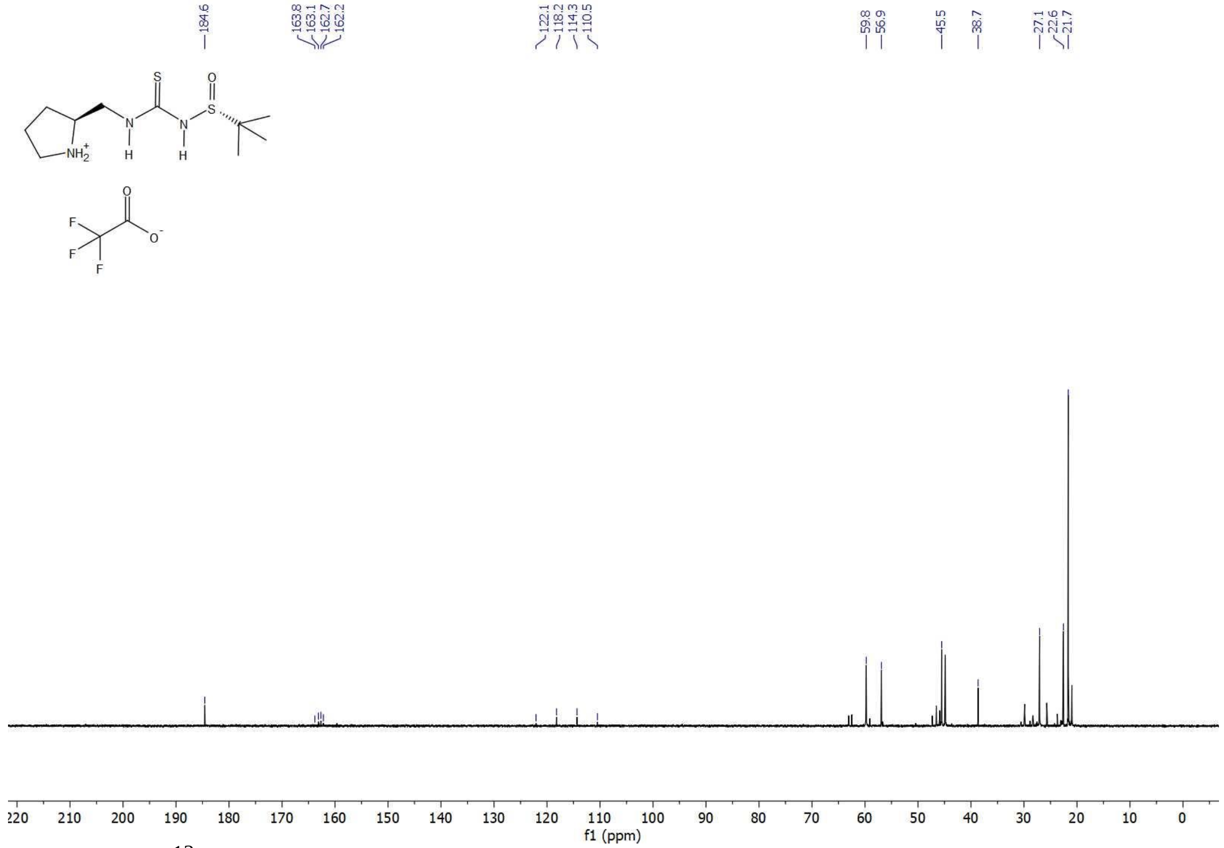

Figure S14. ^{13}C NMR of ***(S,R)*-C1**

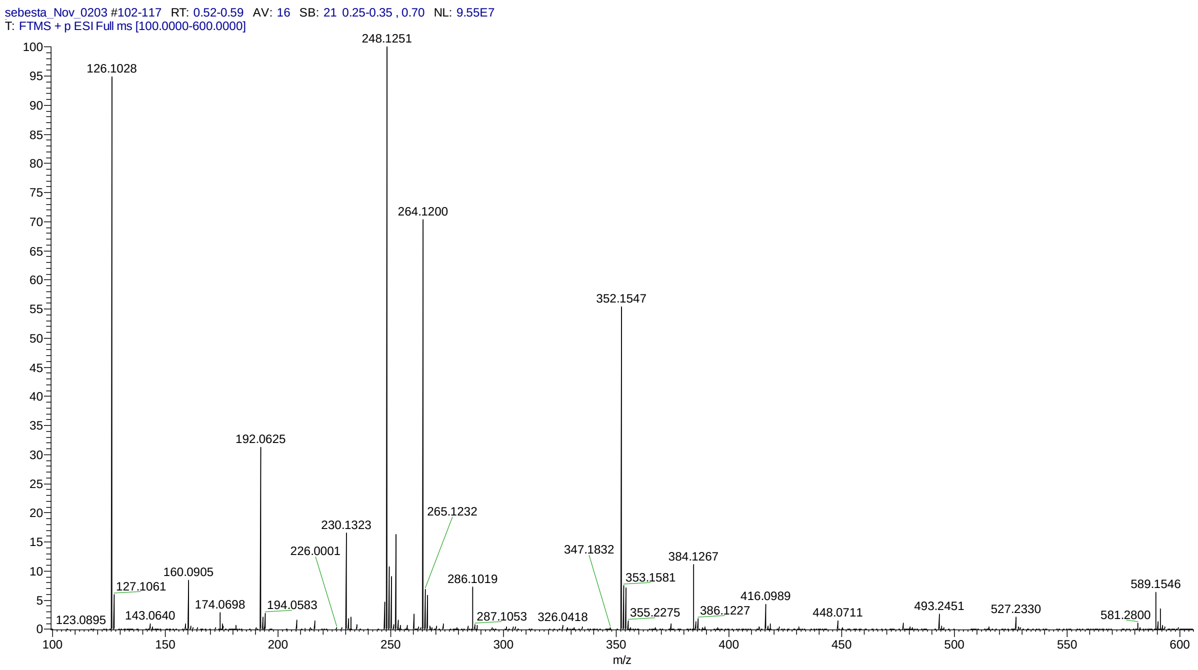

Figure S15. HRMS of **(*S*,*R*)-C1**

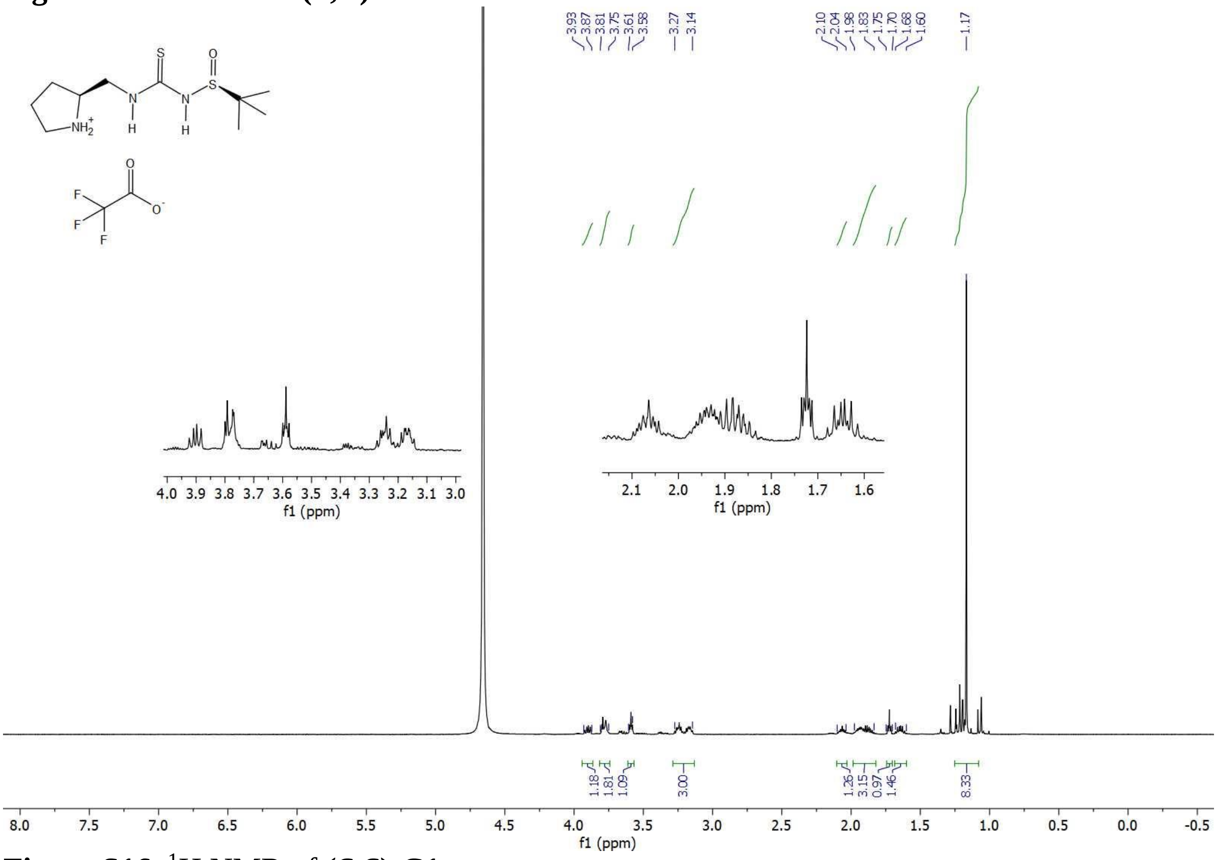

Figure S16. ^{1}H NMR of **(*S*,*S*)-C1**

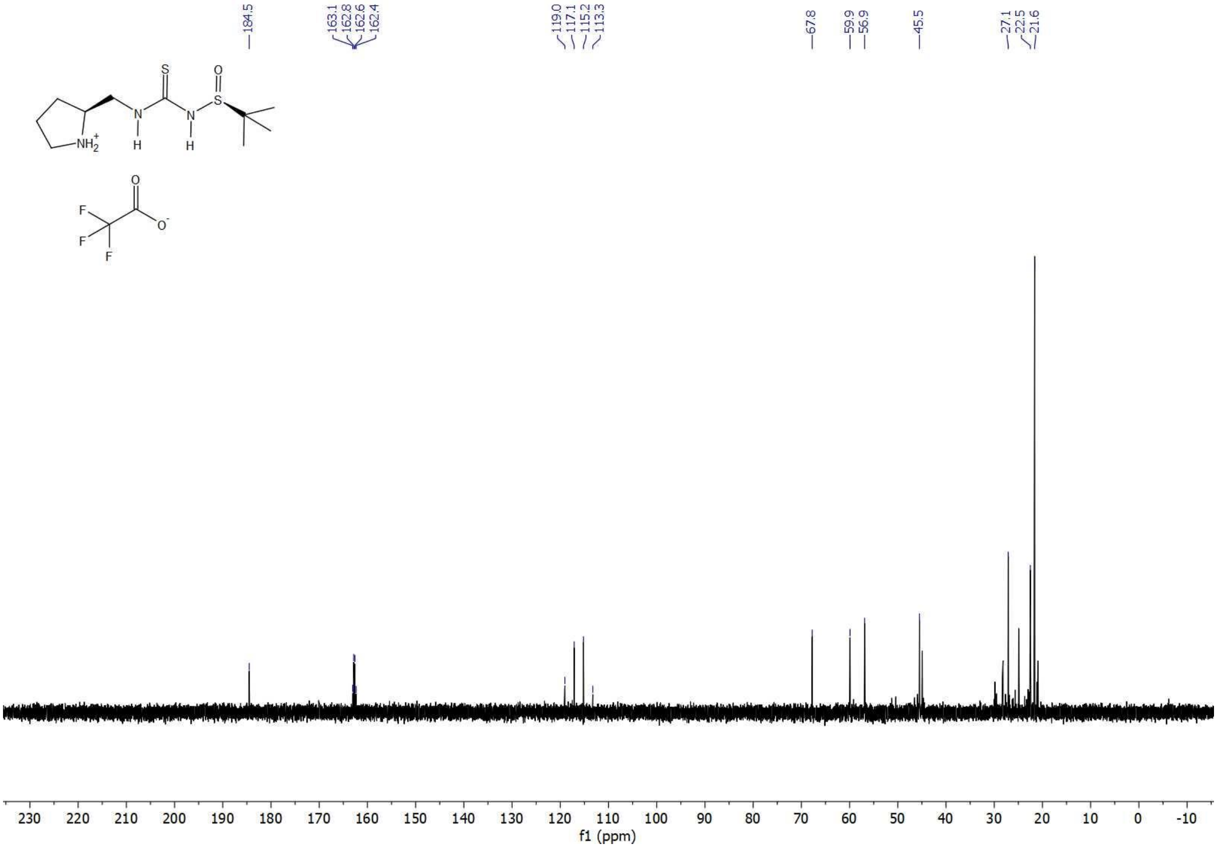

Figure S17. ^{13}C NMR of ***(S,S)*-C1**

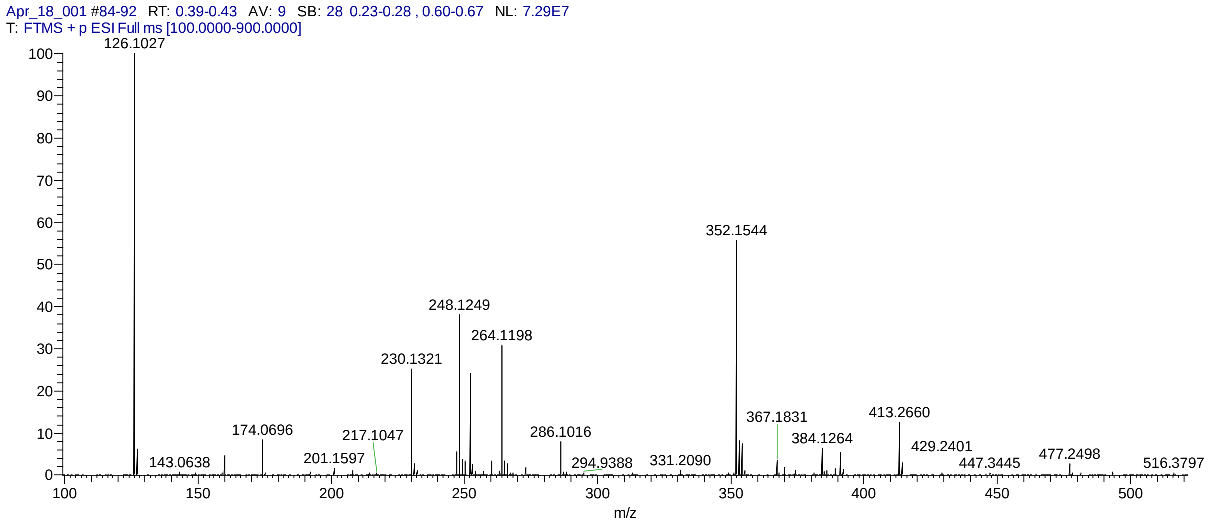

Figure S18. HRMS1 of ***(S,S)*-C1**

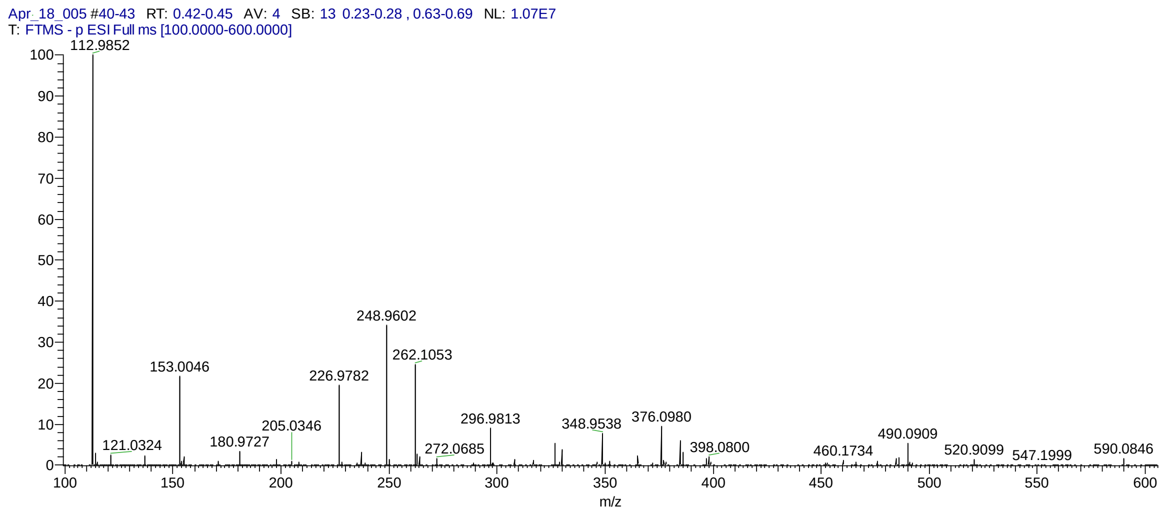

Figure S19. HRMS2 of ***(S,S)*-C1**

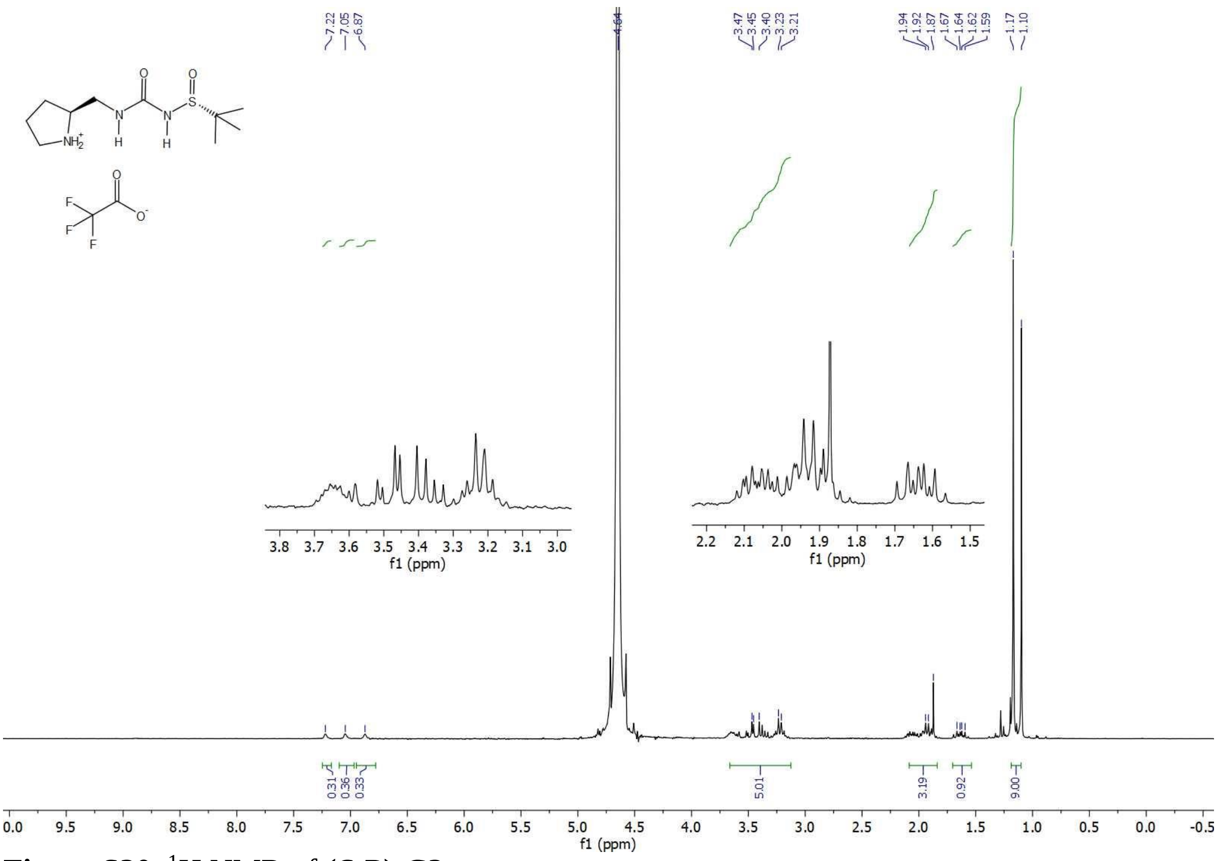

Figure S20. ^{1}H NMR of ***(S,R)*-C2**

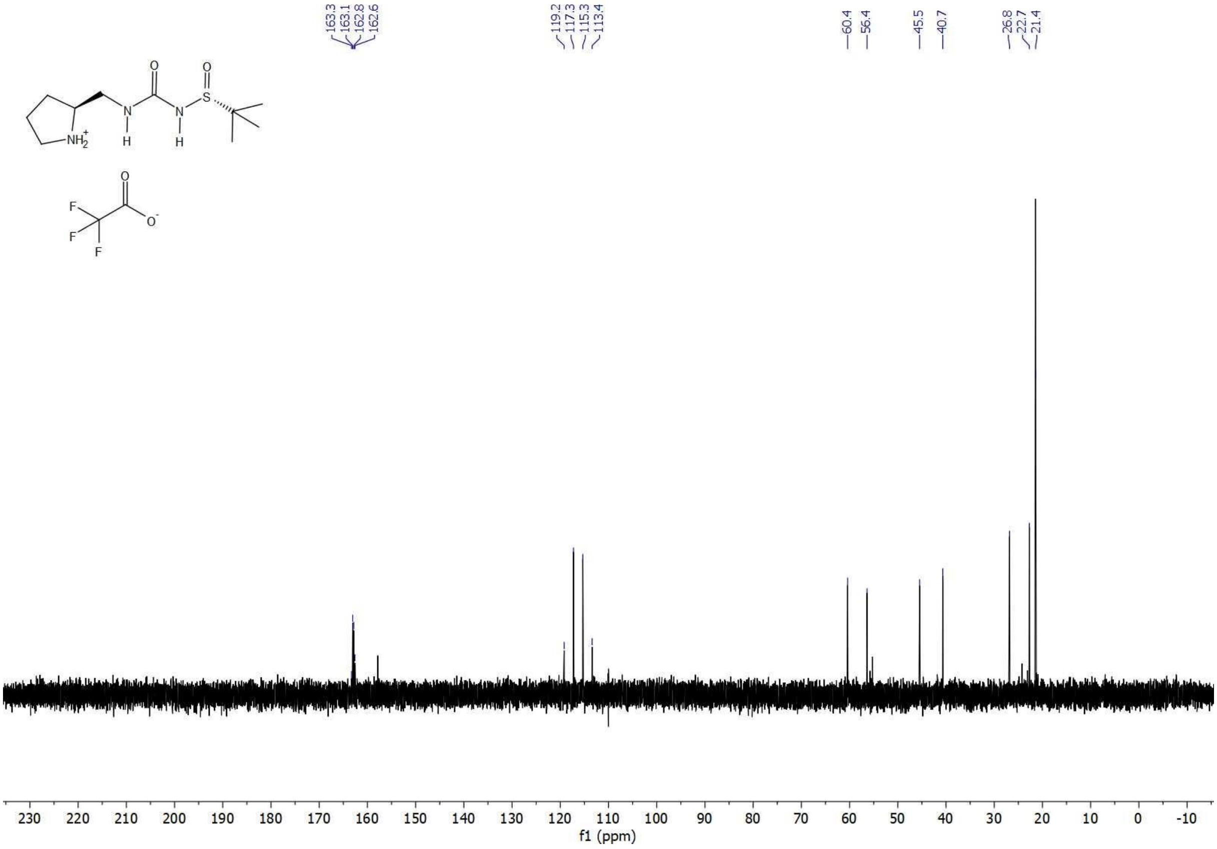

Figure S21. ^{13}C NMR of **(*S*,*R*)-C2**

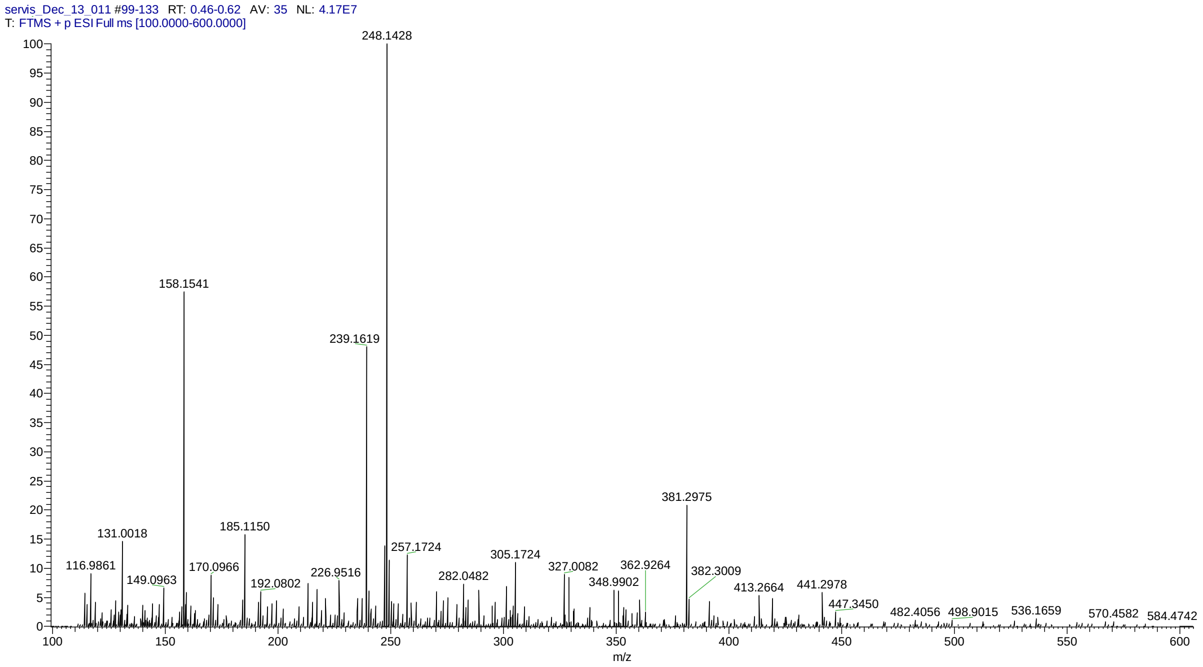

Figure S22. HRMS of **(*S*,*R*)-C2**

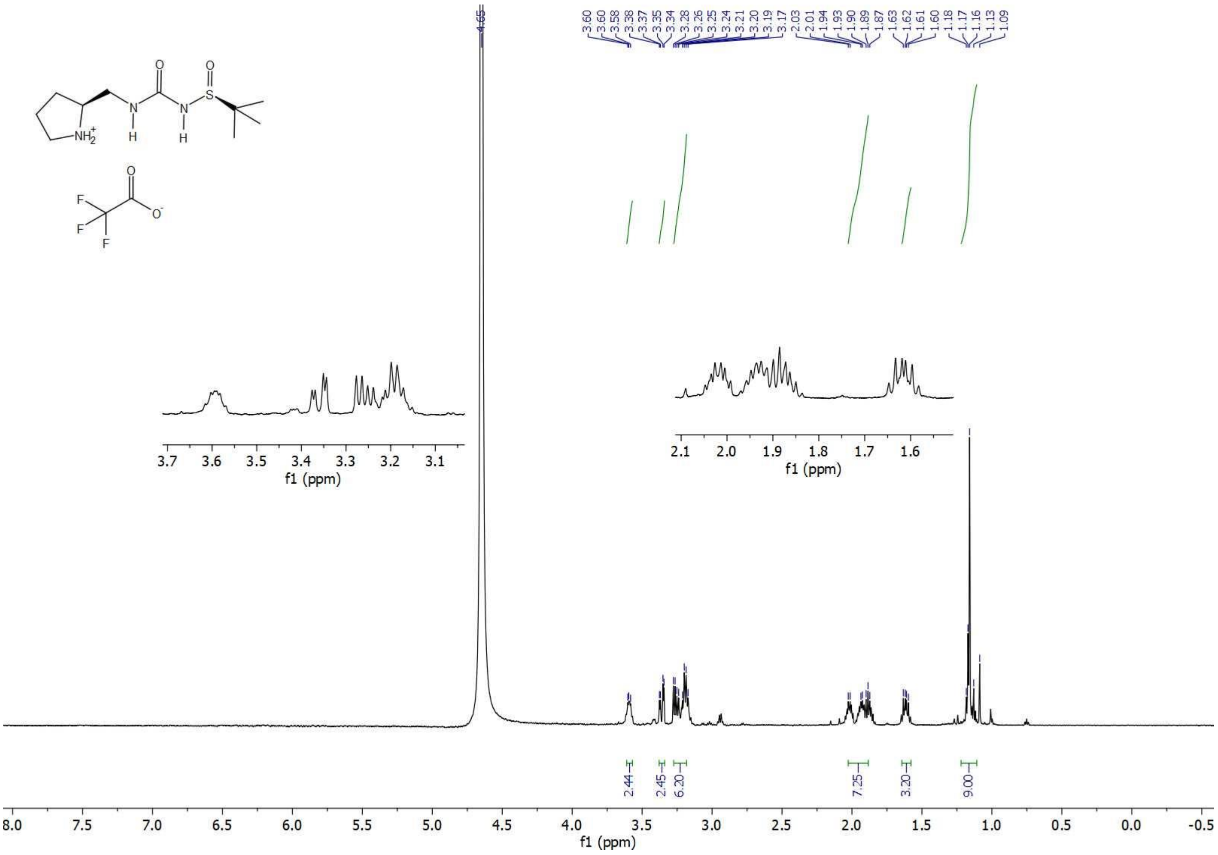

Figure S23. ^{1}H NMR of ***(S,S)*-C2**

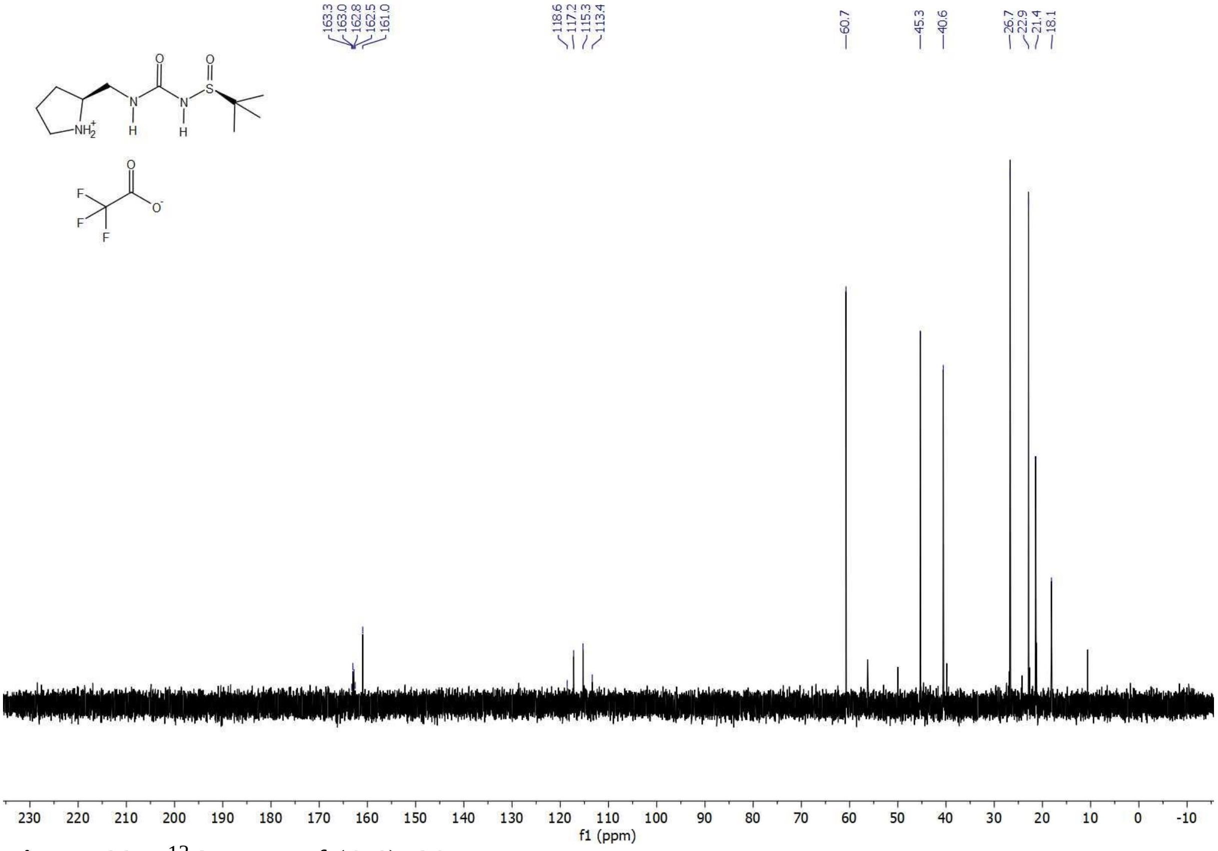

Figure S24. ^{13}C NMR of ***(S,S)*-C2**

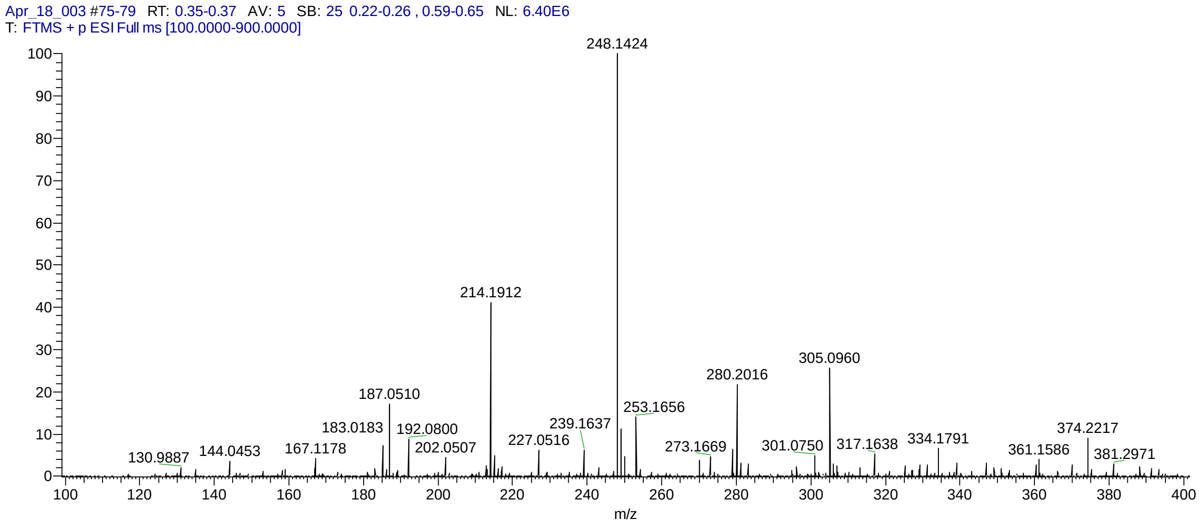

Figure S25. HRMS1 of ***(S,S)*-C2**

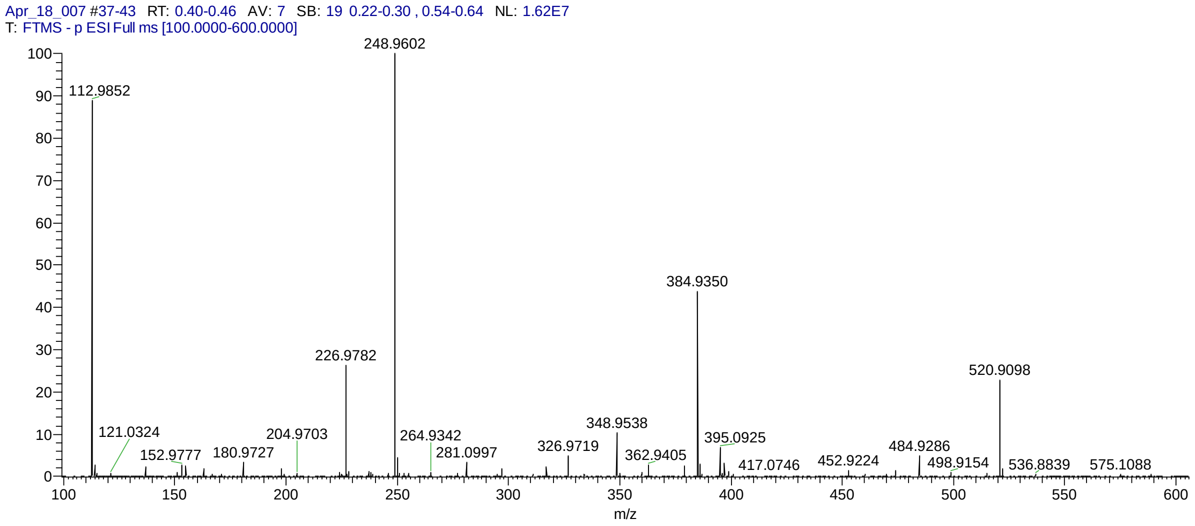

Figure S26. HRMS2 of ***(S,S)*-C2**

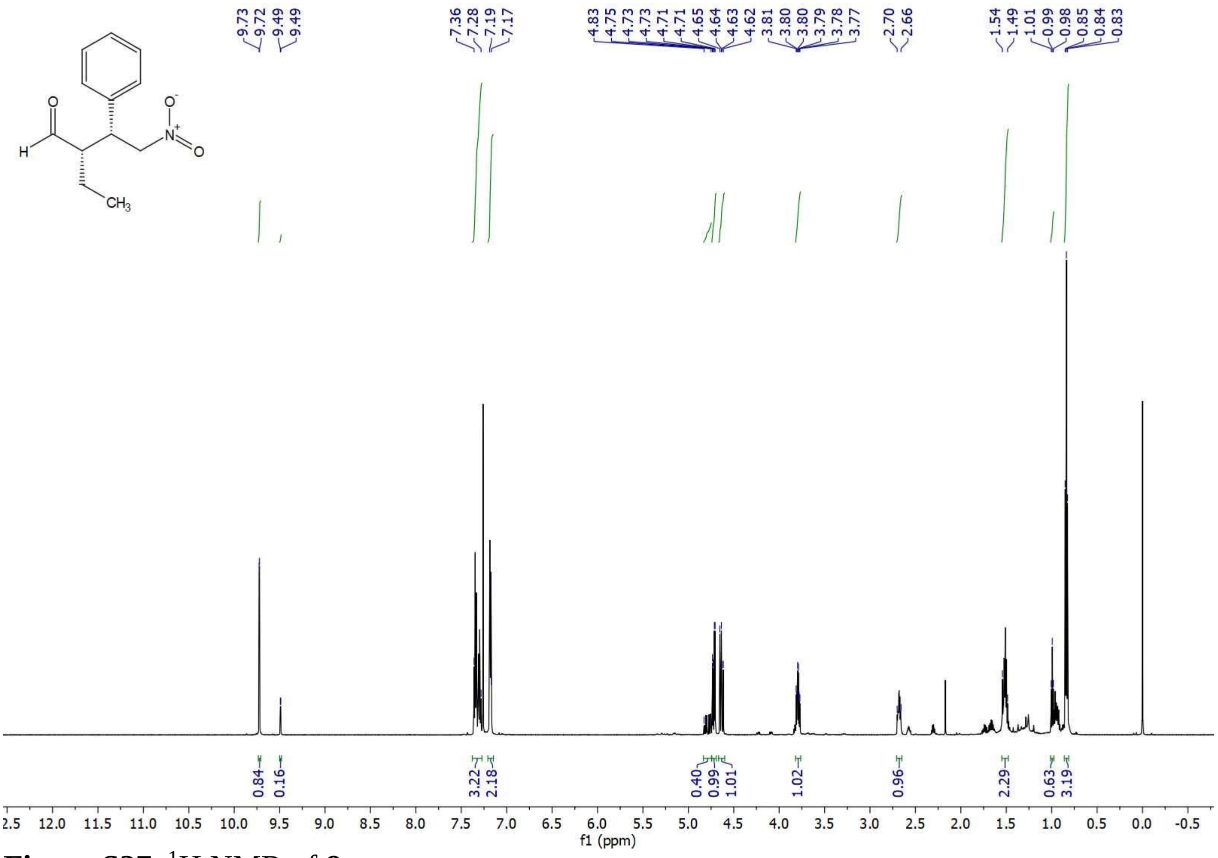

Figure S27. ^{1}H NMR of **8a**

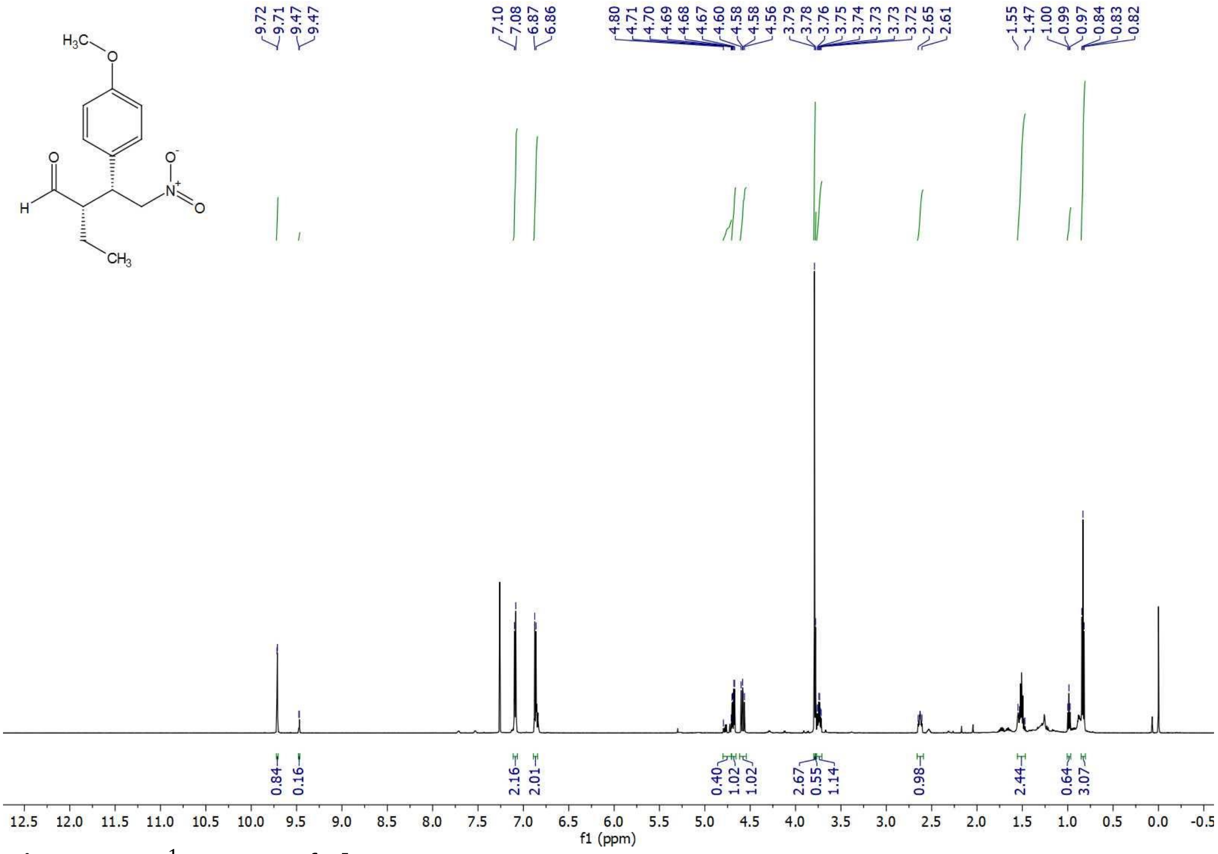

Figure S28. ^{1}H NMR of **8b**

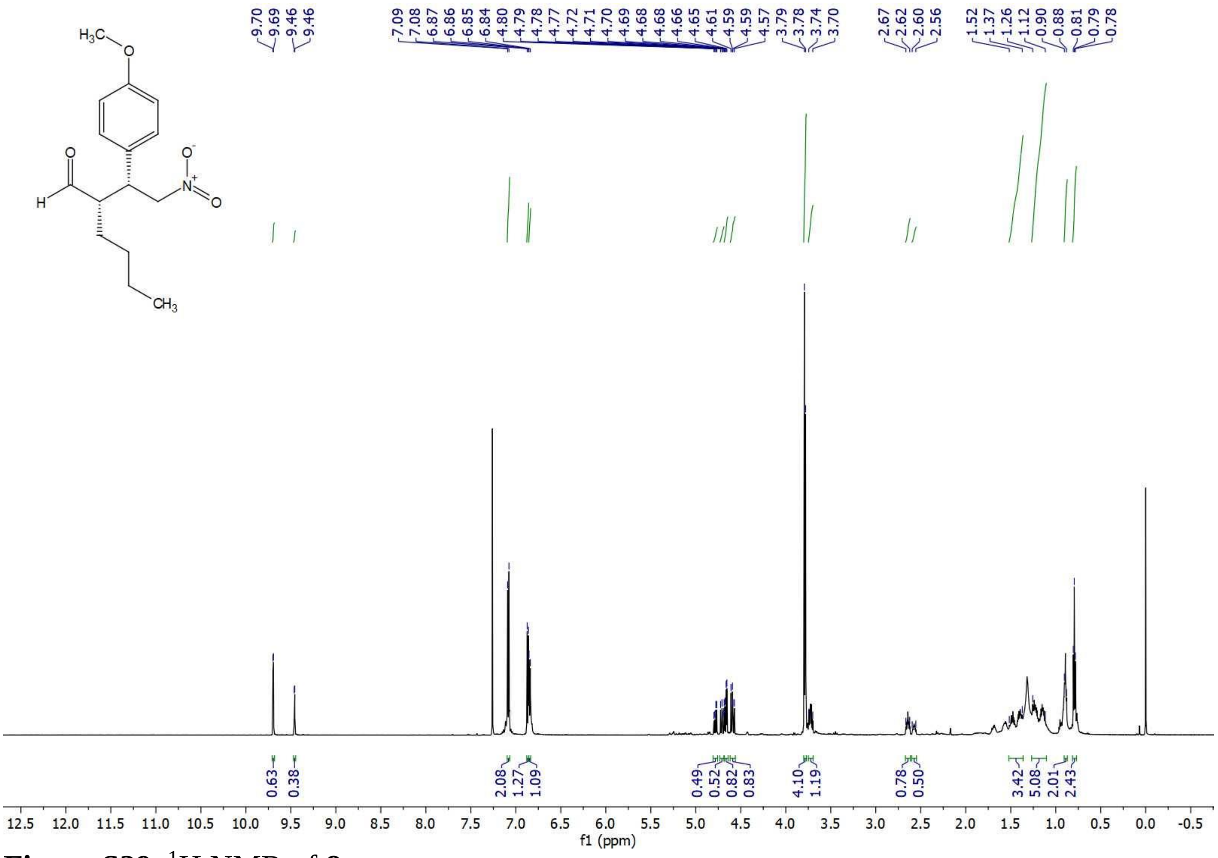

Figure S29. ^{1}H NMR of **8c**

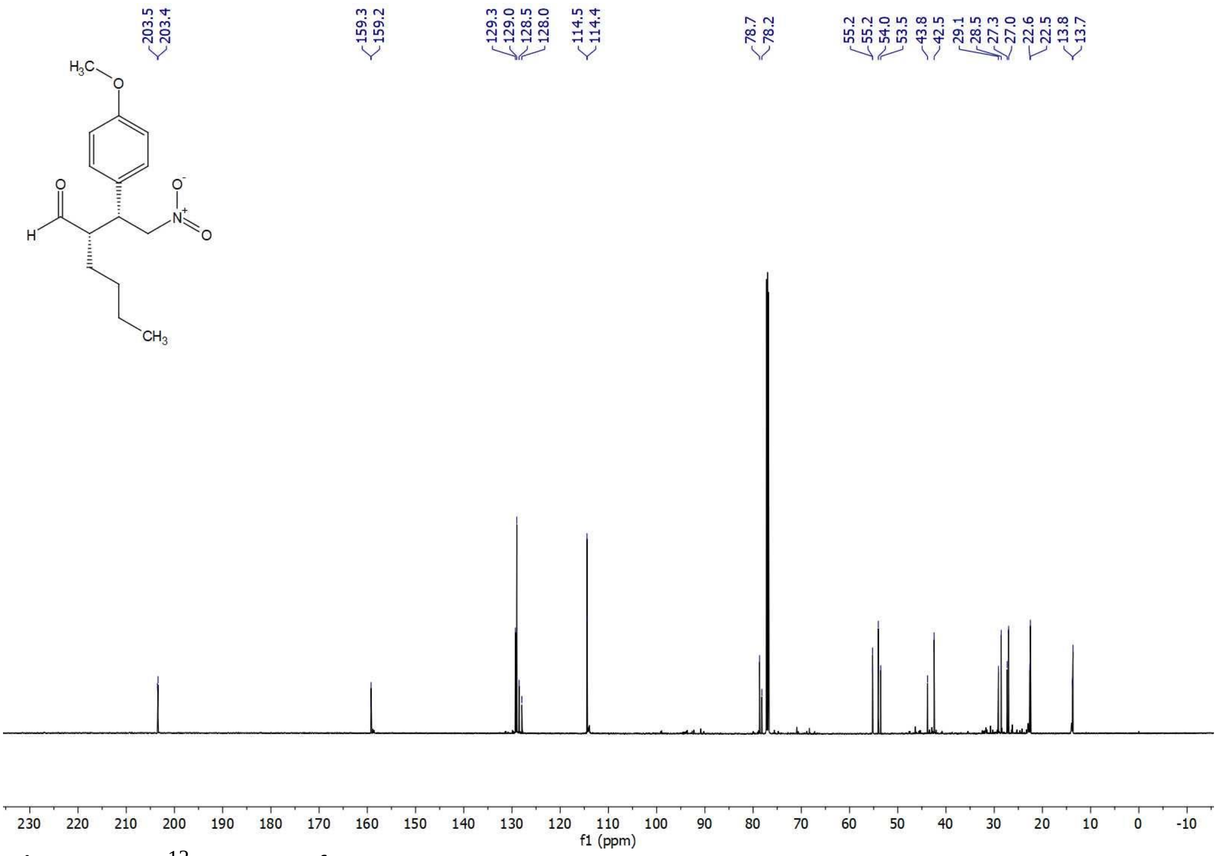

Figure S30. ^{13}C NMR of **8c**

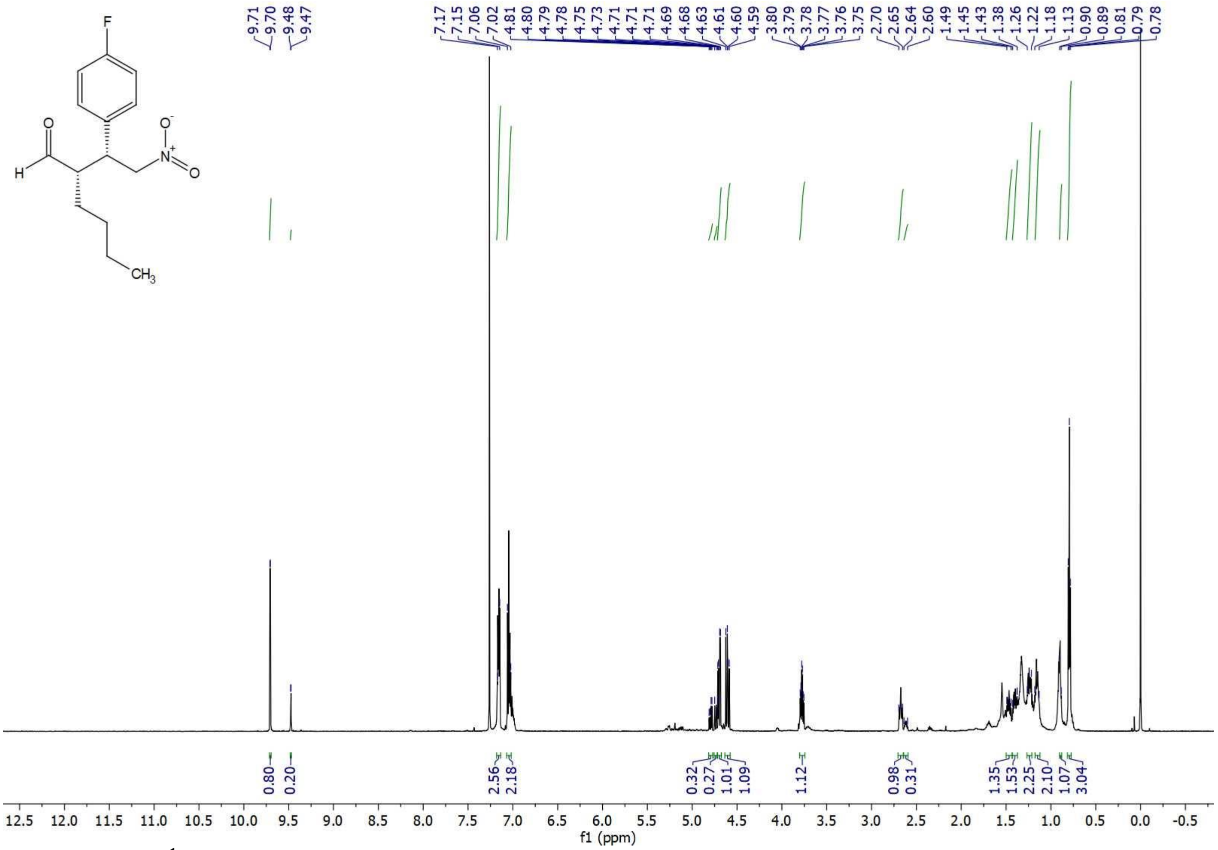

Figure S31. ^{1}H NMR of **8d**

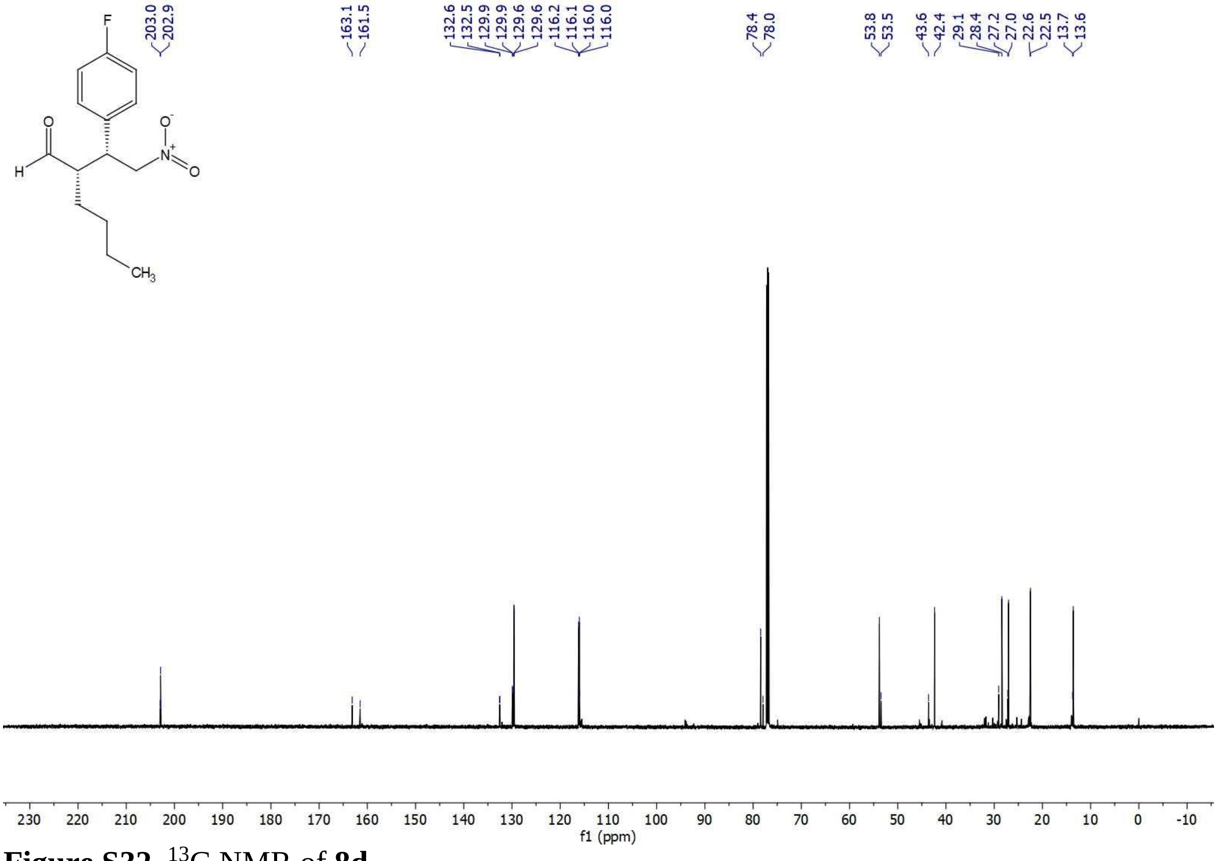

Figure S32. ^{13}C NMR of **8d**

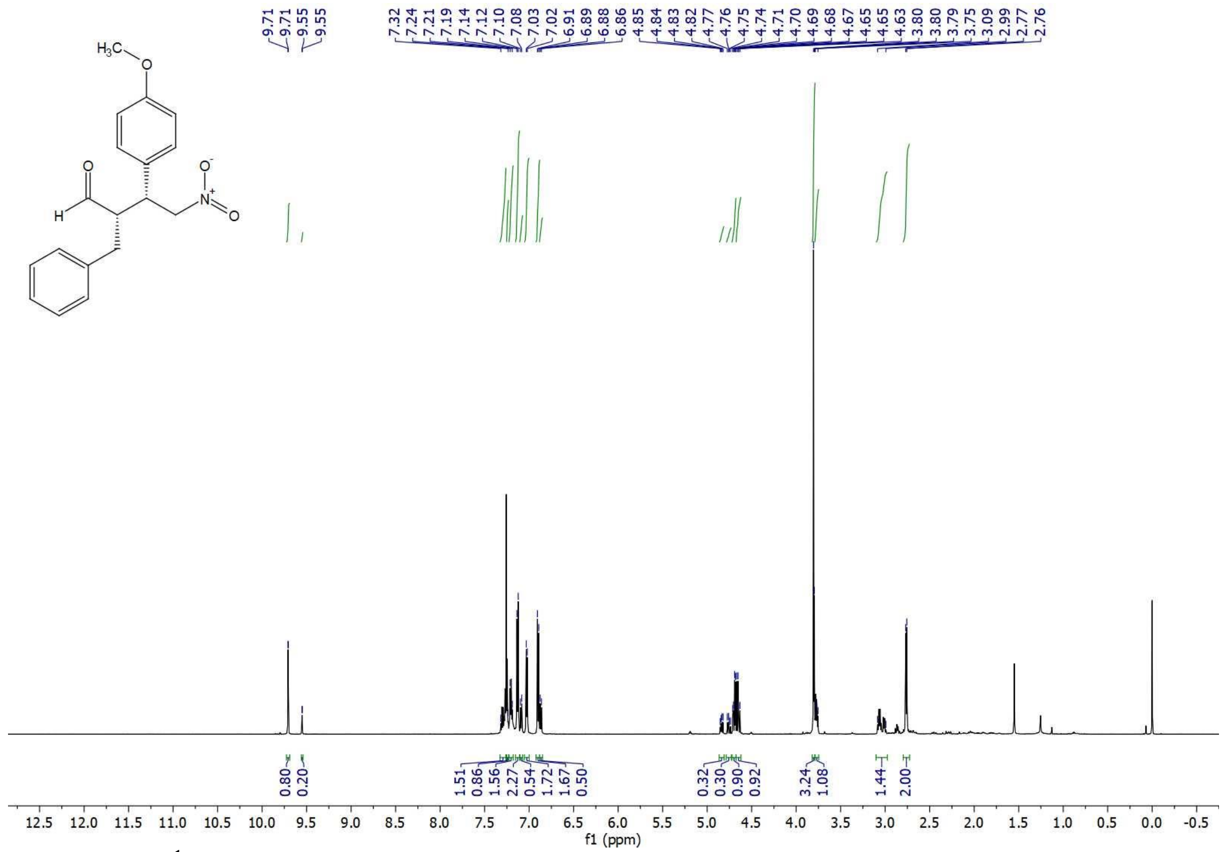

Figure S33. ^{1}H NMR of **8e**

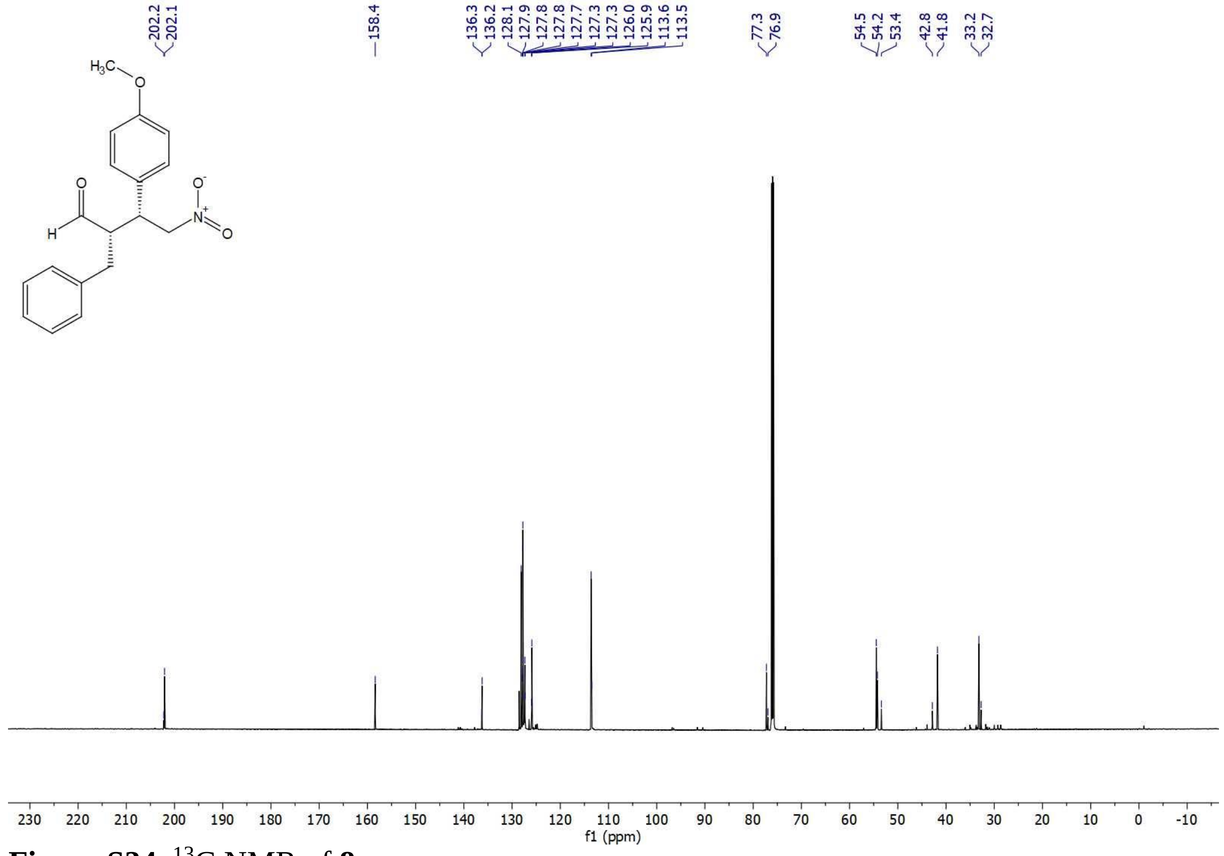

Figure S34. ^{13}C NMR of **8e**

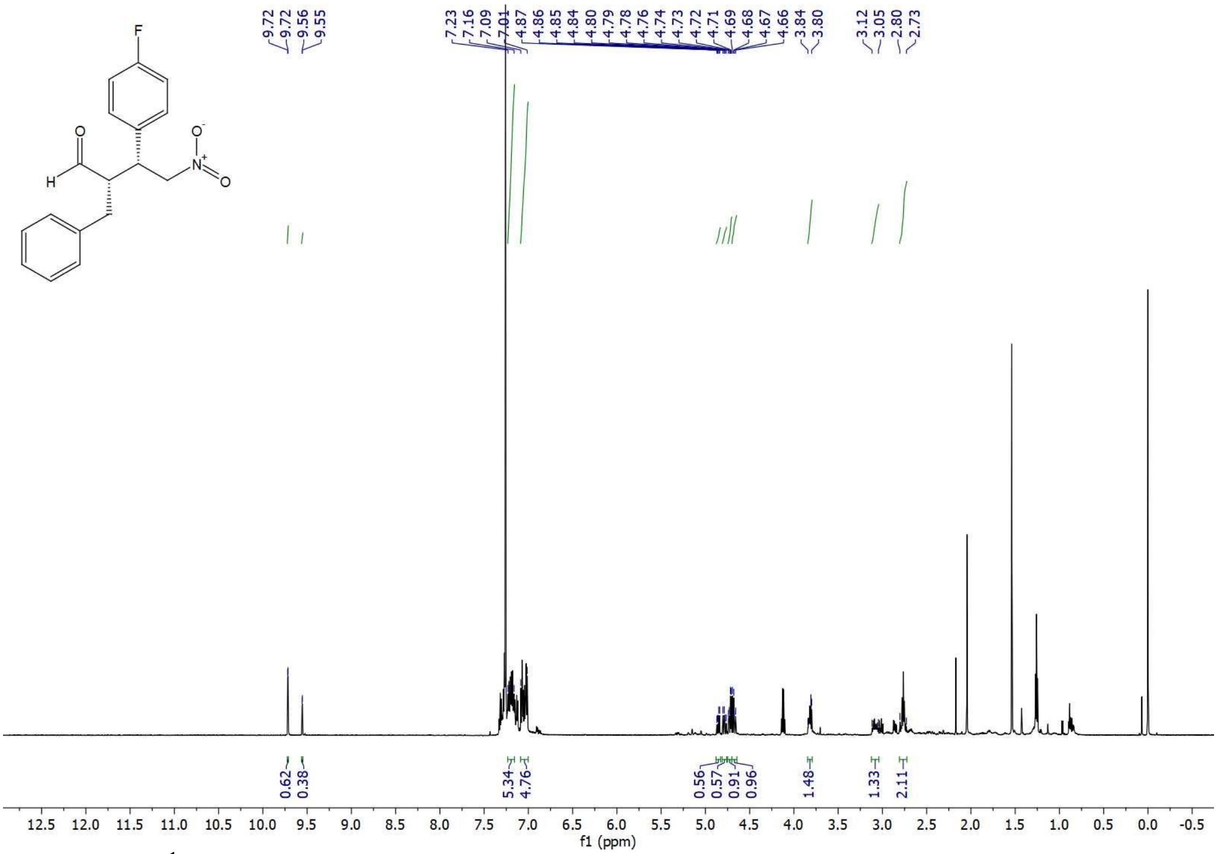

Figure S35. ^{1}H NMR of **8f**

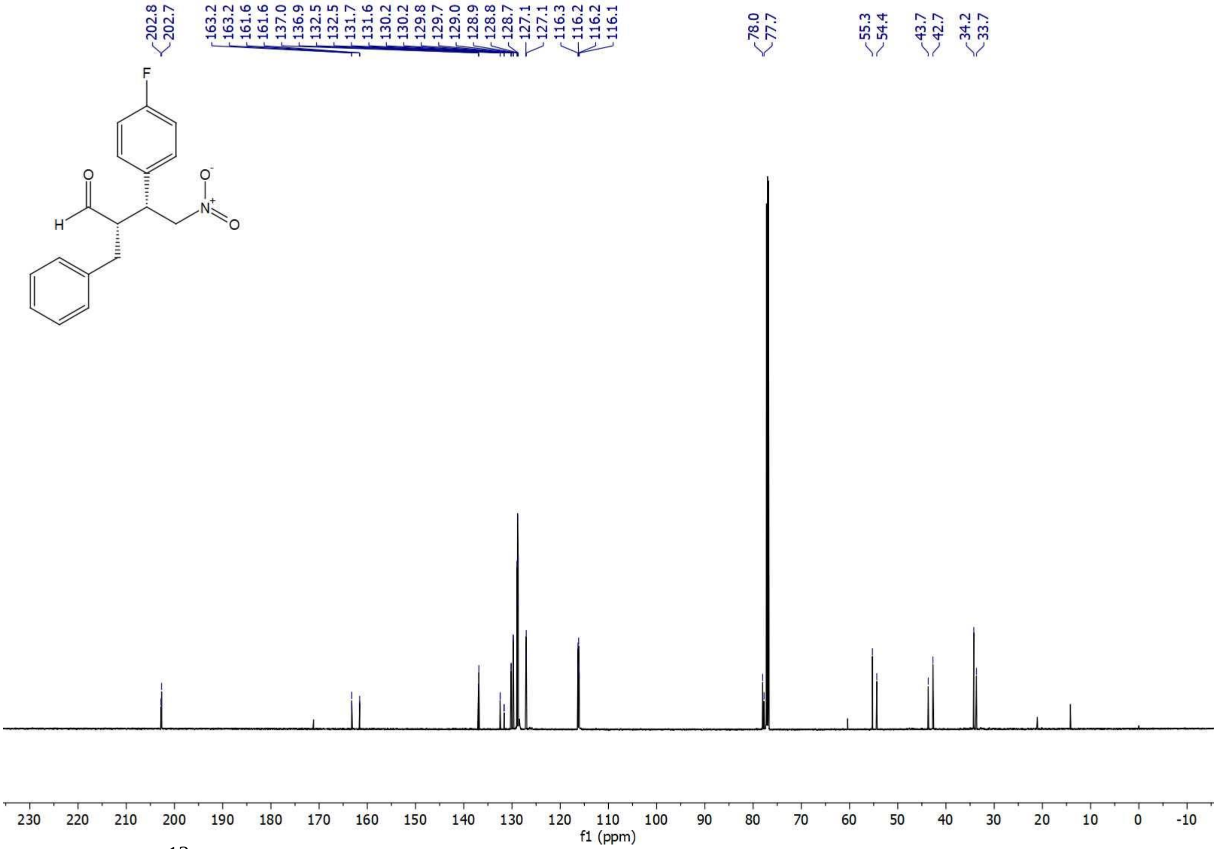

Figure S36. ^{13}C NMR of **8f**

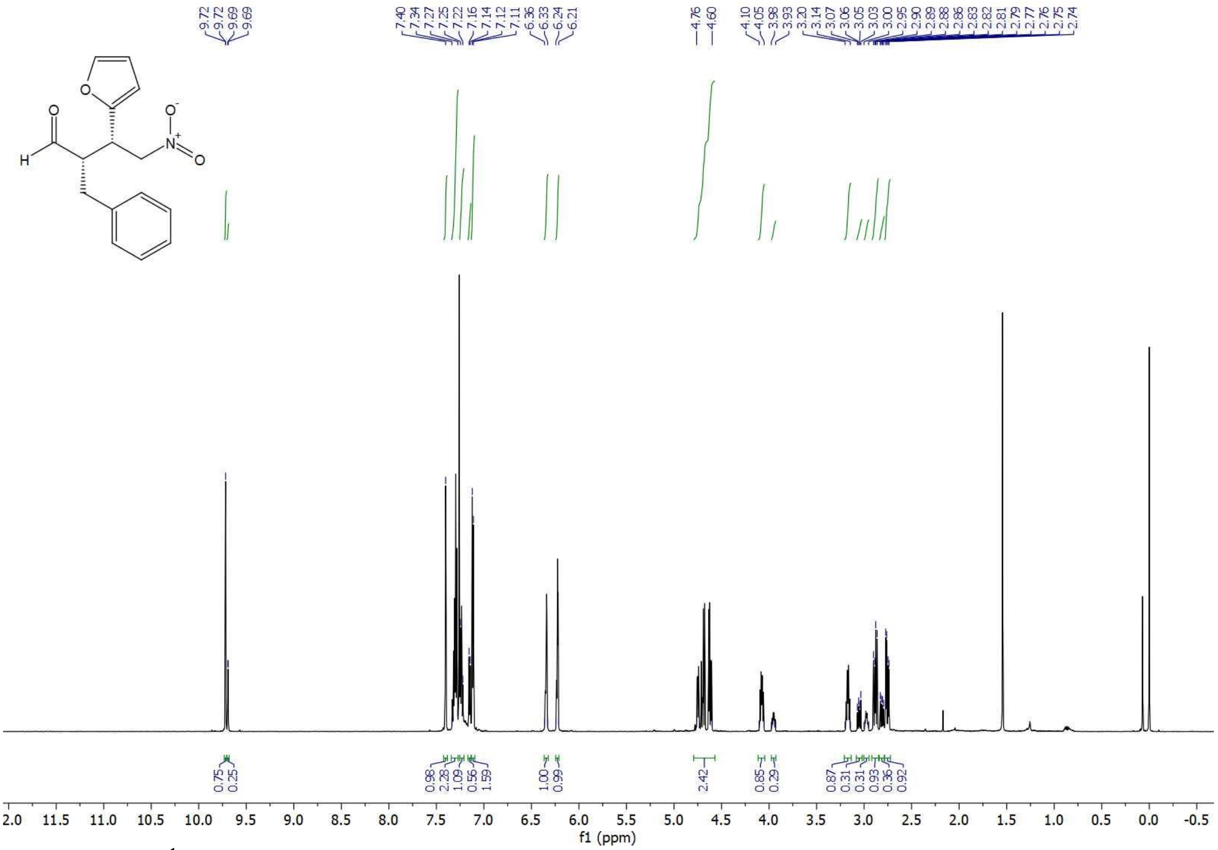

Figure S37. ^{1}H NMR of **10a**

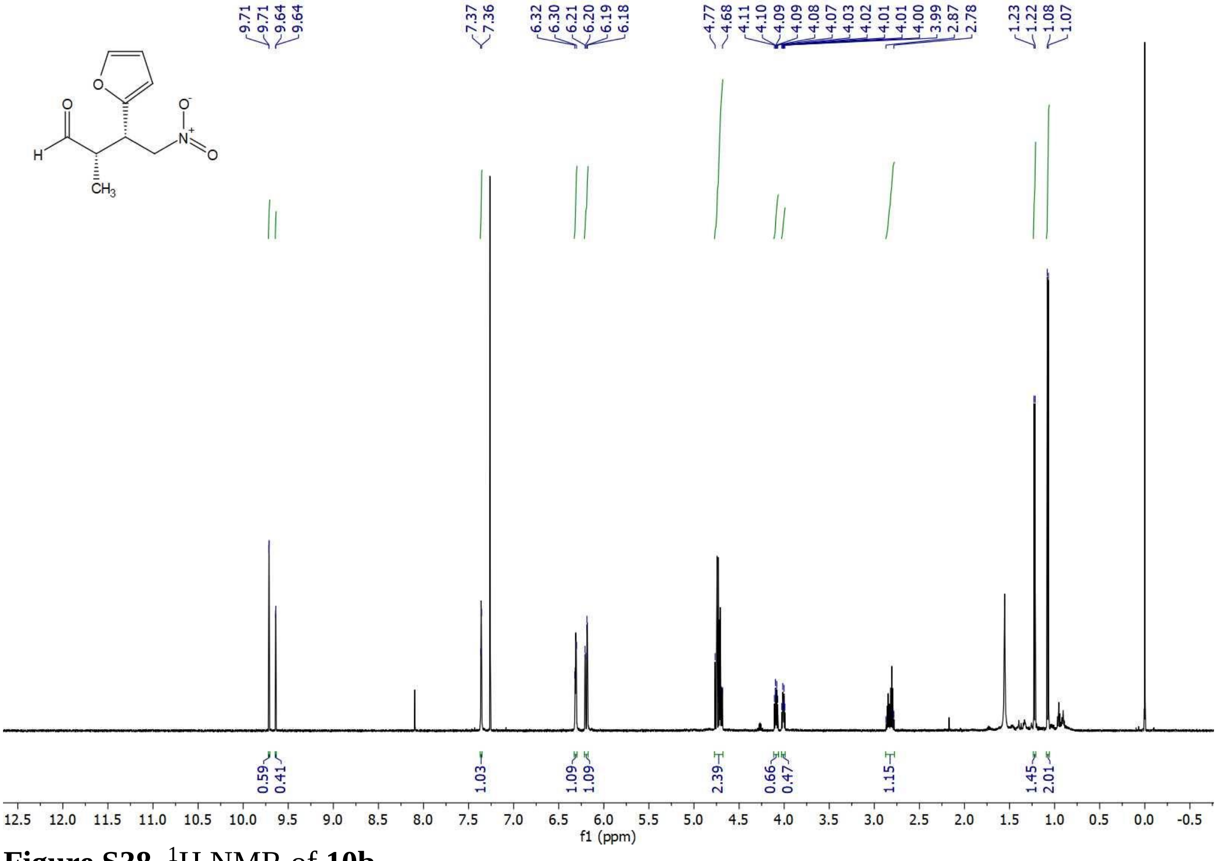

Figure S38. ^{1}H NMR of **10b**

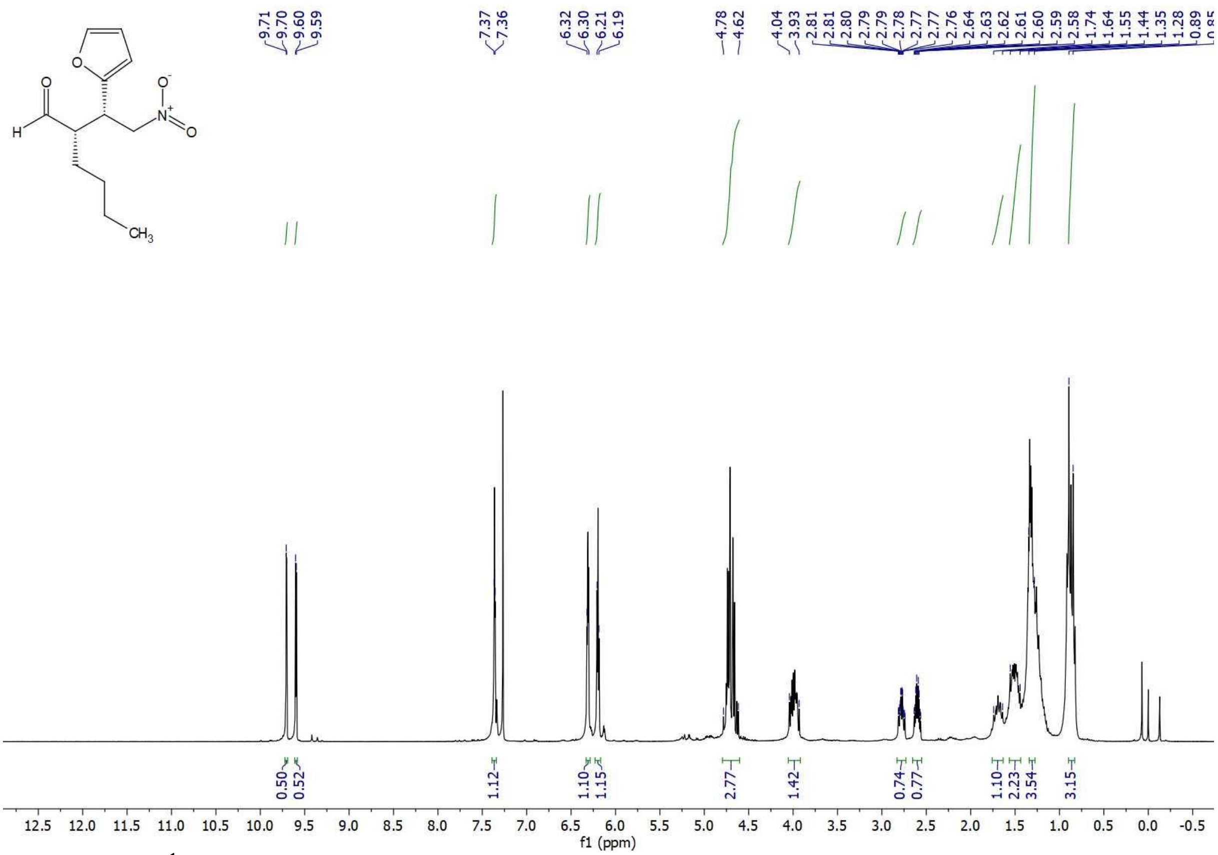

Figure S39. ^{1}H NMR of **10c**

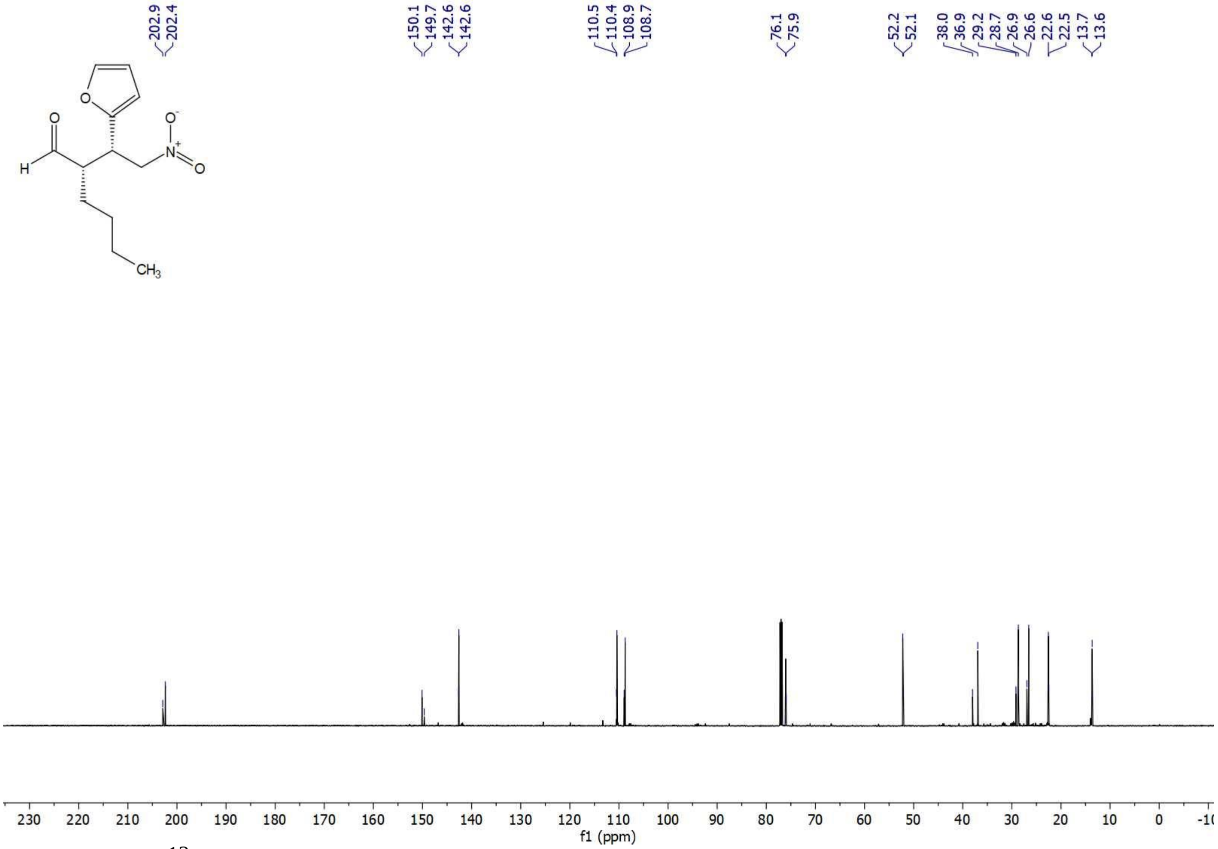

Figure S40. ^{13}C NMR of **10c**

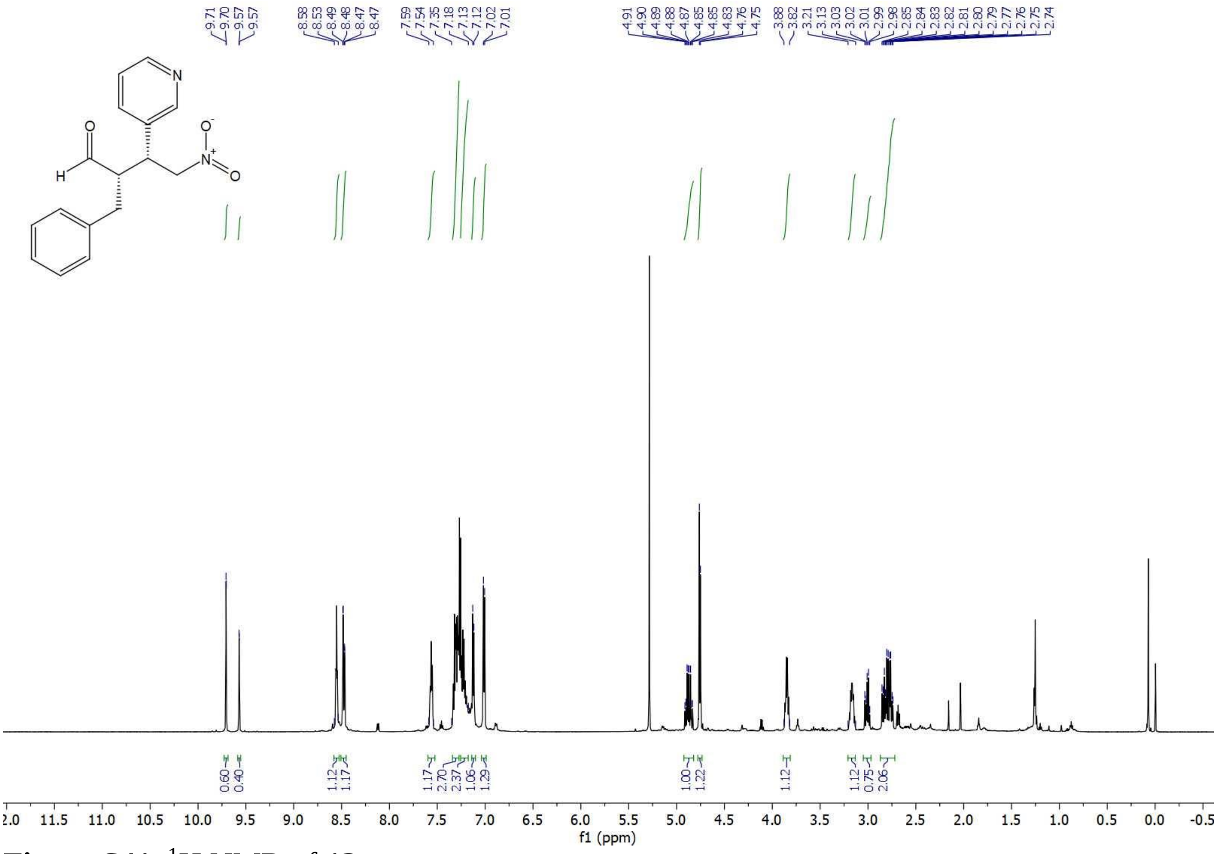

Figure S41. ^{1}H NMR of **12**

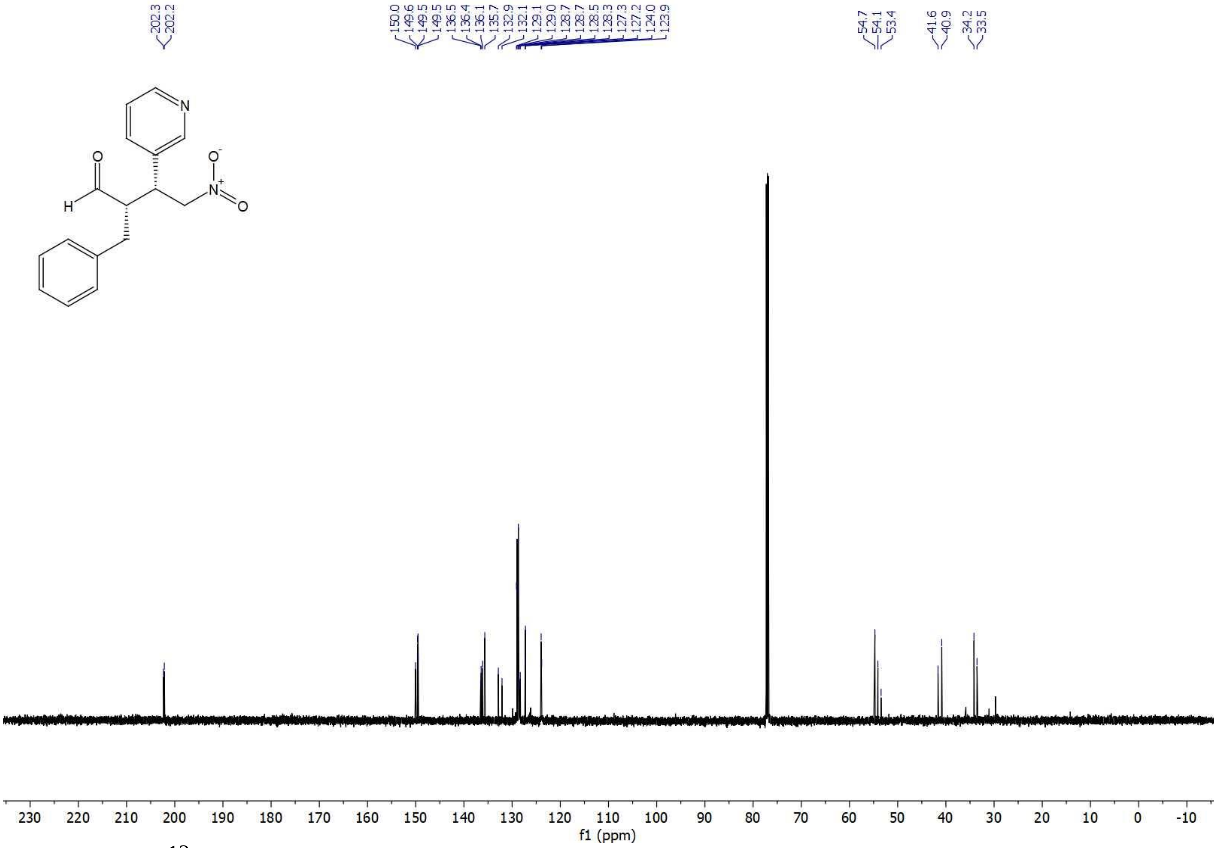

Figure S42. ^{13}C NMR of **12**

4. Copies of HPLC records

a) chiral

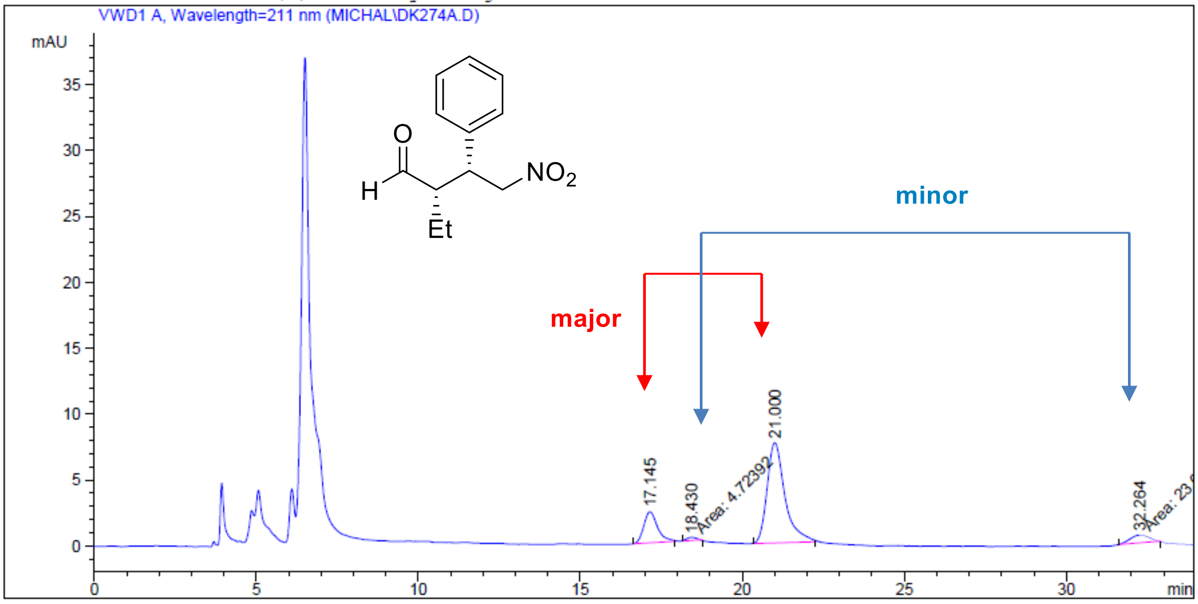

Peak #	RetTime [min]	Type	Width [min]	Area mAU *s	Height [mAU]	Area %
1	17.145	BB	0.4454	69.50437	2.36074	17.8788
2	18.430	MM	0.3304	4.72392	2.38321e-1	1.2151
3	21.000	BB	0.5710	290.85626	7.61127	74.8177
4	32.264	MM	0.6692	23.66880	5.89492e-1	6.0884

b) racemic

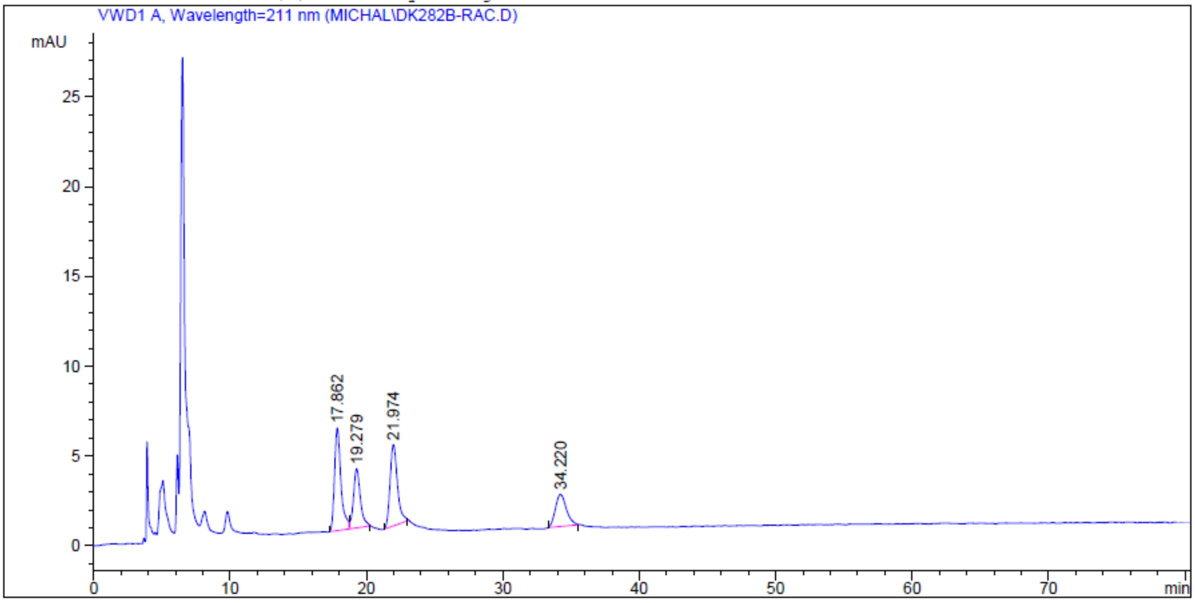

Peak #	RetTime [min]	Type	Width [min]	Area mAU *s	Height [mAU]	Area %
1	17.862	BB	0.4909	186.76668	5.70906	32.6174
2	19.279	BB	0.5238	115.60297	3.28738	20.1892
3	21.974	BB	0.5755	170.00082	4.52189	29.6894
4	34.220	BB	0.7422	100.22718	1.78969	17.5039

Figure S43. HPLC trace of **8a**

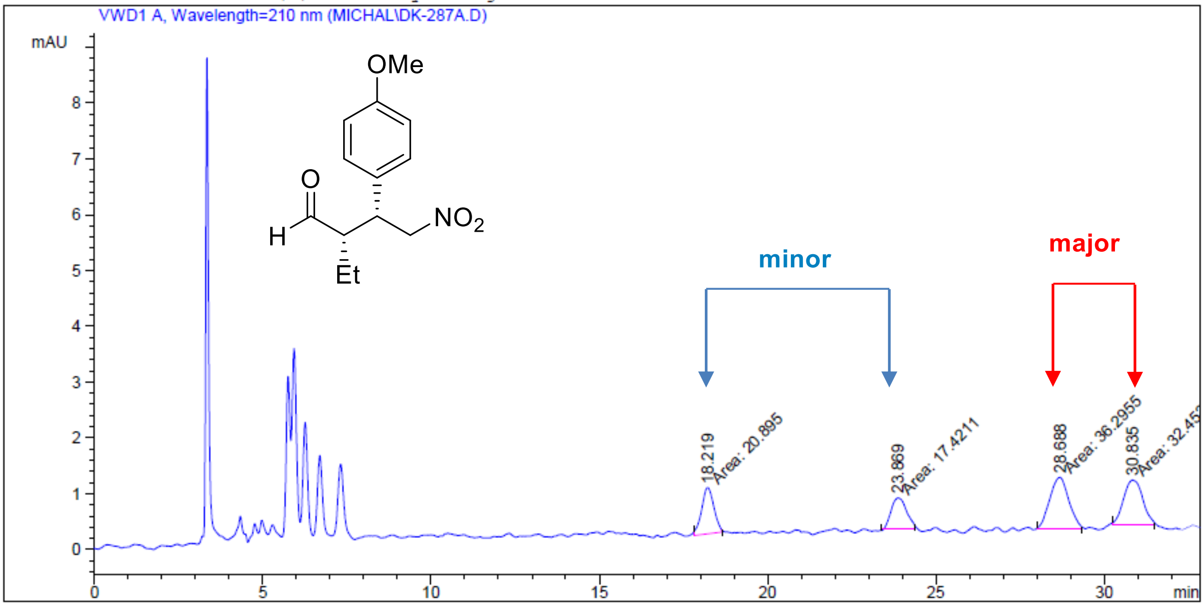

Peak #	RetTime [min]	Type	Width [min]	Area mAU *s	Height [mAU]	Area %
1	18.219	MM	0.4205	20.89495	8.28197e-1	19.5162
2	23.869	MM	0.5247	17.42112	5.53404e-1	16.2716
3	28.688	MM	0.6567	36.29554	9.21120e-1	33.9006
4	30.835	MM	0.6661	32.45287	8.11955e-1	30.3115

Figure S44. HPLC trace of **8b** (racemic)

a) chiral

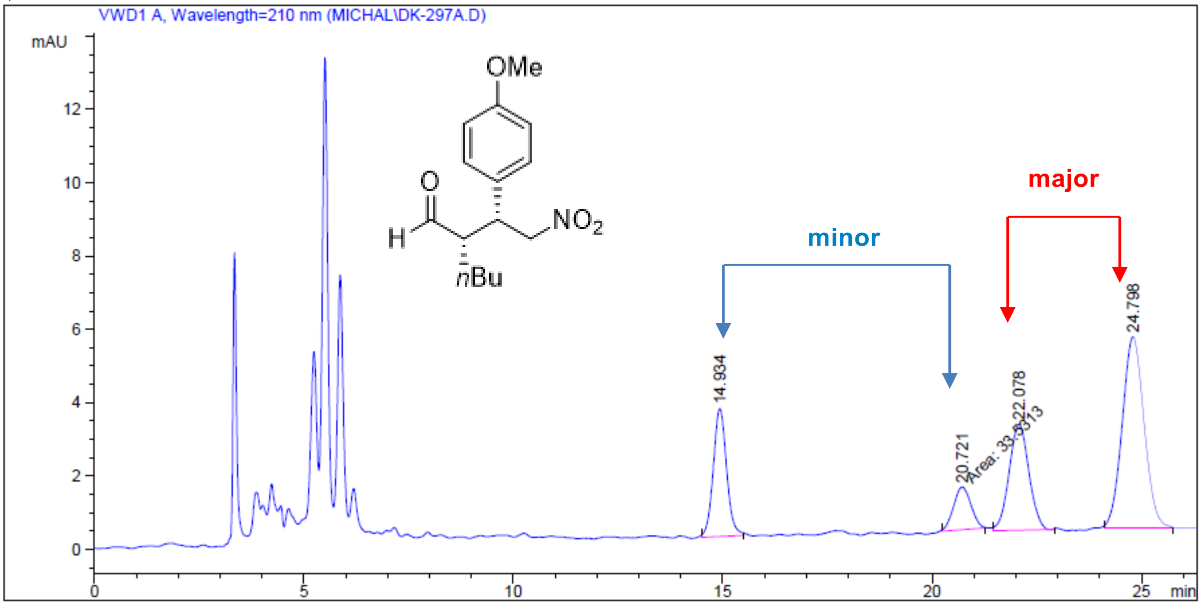

Peak #	RetTime [min]	Type	Width [min]	Area mAU *s	Height [mAU]	Area %
1	14.934	BB	0.3376	77.00037	3.48780	19.5584
2	20.721	MM	0.4785	33.53126	1.16787	8.5171
3	22.078	BB	0.5113	94.90208	2.88984	24.1055
4	24.798	BB	0.5562	188.26102	5.20222	47.8190

b) racemic

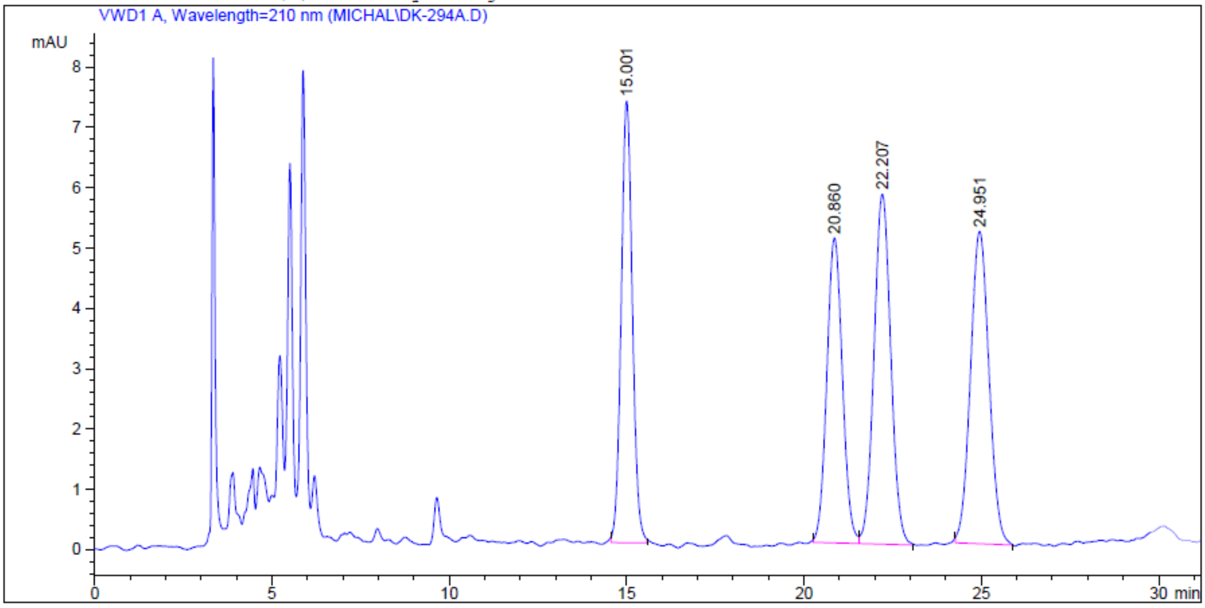

Peak #	RetTime [min]	Type	Width [min]	Area mAU *s	Height [mAU]	Area %
1	15.001	BB	0.3380	160.01204	7.32058	23.0781
2	20.860	BV	0.4789	154.47105	5.05434	22.2789
3	22.207	VB	0.5132	190.72850	5.80097	27.5082
4	24.951	BB	0.5563	188.13889	5.17905	27.1347

Figure S45. HPLC trace of **8c**

a) chiral

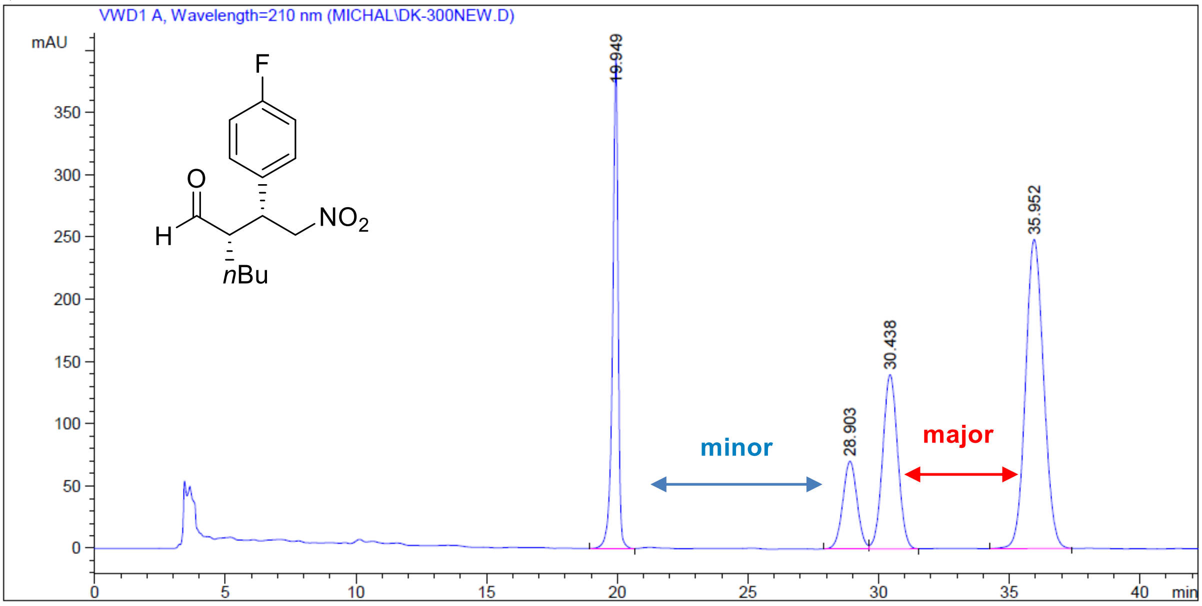

Peak #	RetTime [min]	Type	Width [min]	Area mAU *s	Height [mAU]	Area %
1	19.949	BB	0.2138	5705.87549	394.69928	21.9846
2	28.903	BV	0.5981	2681.69531	70.50391	10.3325
3	30.438	VB	0.6243	5602.06152	139.92654	21.5846
4	35.952	BB	0.7472	1.19643e4	248.24681	46.0983

b) racemic

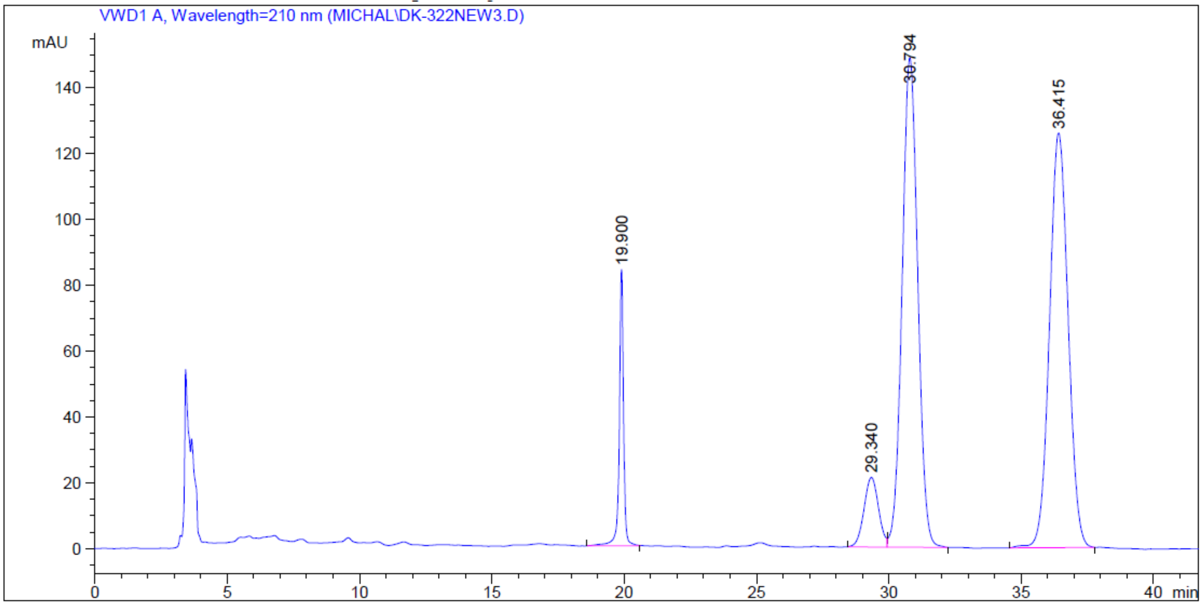

Peak #	RetTime [min]	Type	Width [min]	Area mAU *s	Height [mAU]	Area %
1	19.900	BB	0.1624	939.63080	84.08176	6.7002
2	29.340	BV	0.5993	810.74005	21.18549	5.7811
3	30.794	VB	0.6385	6102.23535	148.84152	43.5129
4	36.415	BB	0.7605	6171.35889	126.03487	44.0058

Figure S46. HPLC trace of **8d**

a) chiral

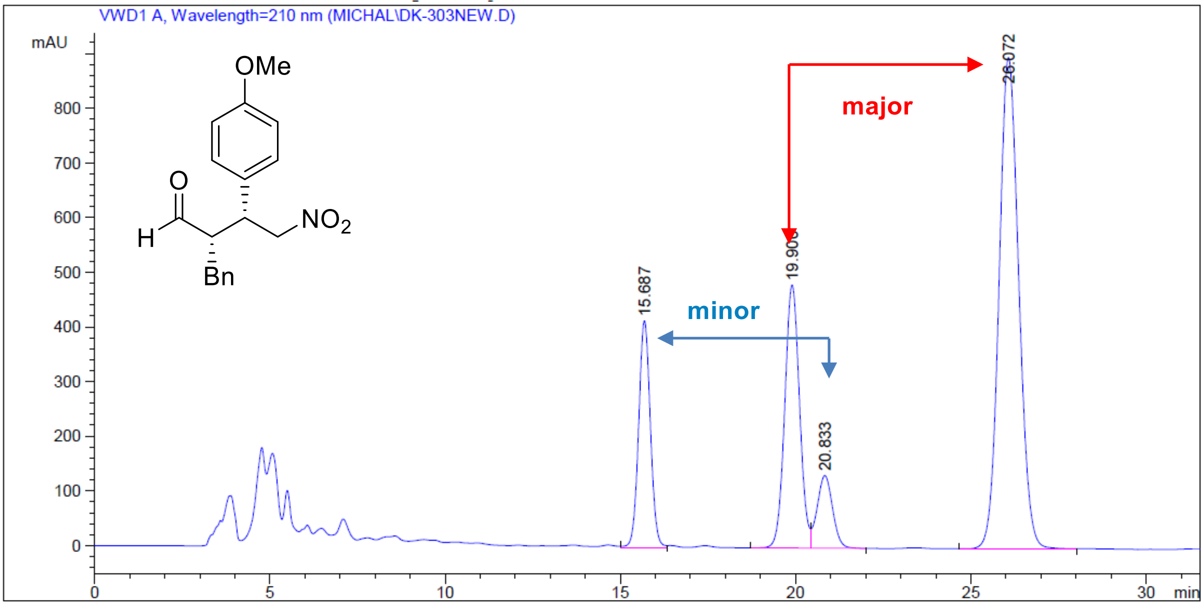

Peak #	RetTime [min]	Type	Width [min]	Area mAU *s	Height [mAU]	Area %
1	15.687	VV	0.3600	9647.27539	414.96320	15.3298
2	19.900	VV	0.4557	1.41142e4	481.31055	22.4278
3	20.833	VB	0.4741	4068.56299	132.73634	6.4651
4	26.072	BB	0.6058	3.51016e4	898.48590	55.7774

b) racemic

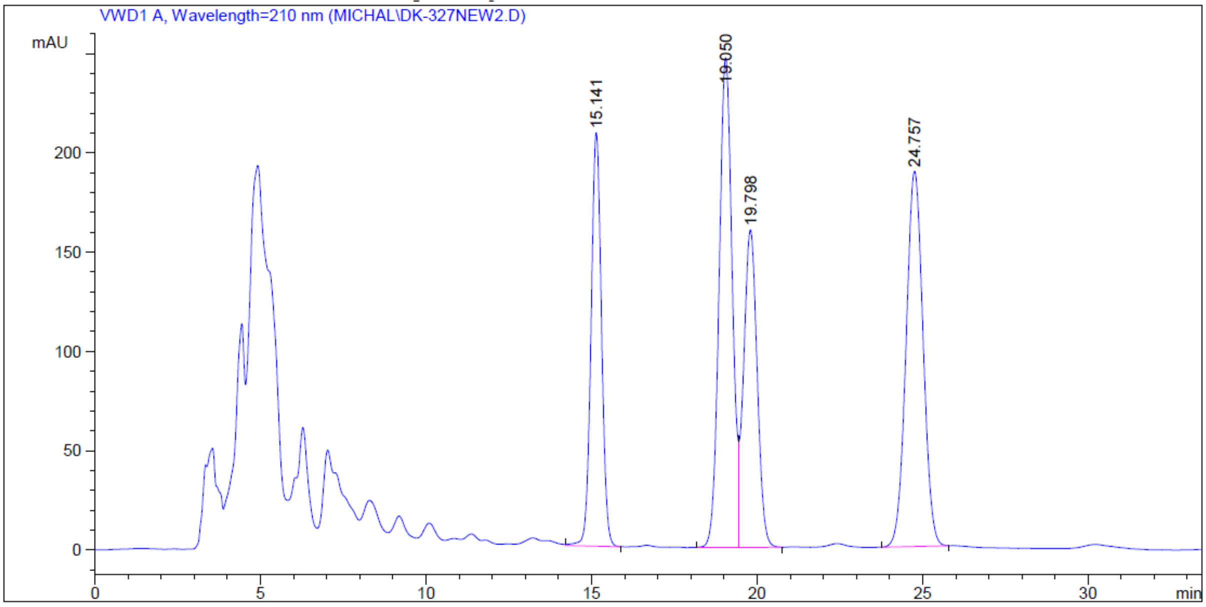

Peak #	RetTime [min]	Type	Width [min]	Area mAU *s	Height [mAU]	Area %
1	15.141	VB	0.3383	4558.43652	208.27930	20.1474
2	19.050	BV	0.4237	6773.48535	246.68860	29.9375
3	19.798	VB	0.4376	4542.65479	159.97713	20.0776
4	24.757	BB	0.5564	6750.88086	189.05197	29.8375

Figure S47. HPLC trace of **8e**

a) chiral

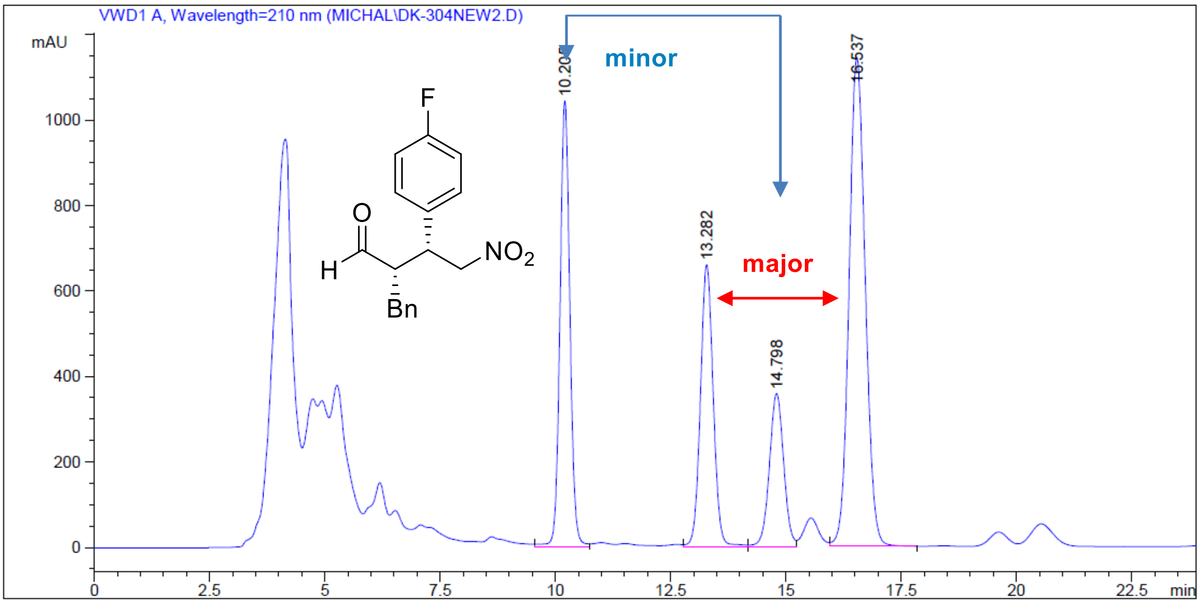

Peak #	RetTime [min]	Type	Width [min]	Area mAU *s	Height [mAU]	Area %
1	10.205	BV	0.2317	1.56443e4	1043.22705	24.4991
2	13.282	VV	0.2988	1.26890e4	659.22009	19.8710
3	14.798	VV	0.3326	7696.46533	357.67654	12.0527
4	16.537	VB	0.3770	2.78270e4	1143.98840	43.5772

b) racemic

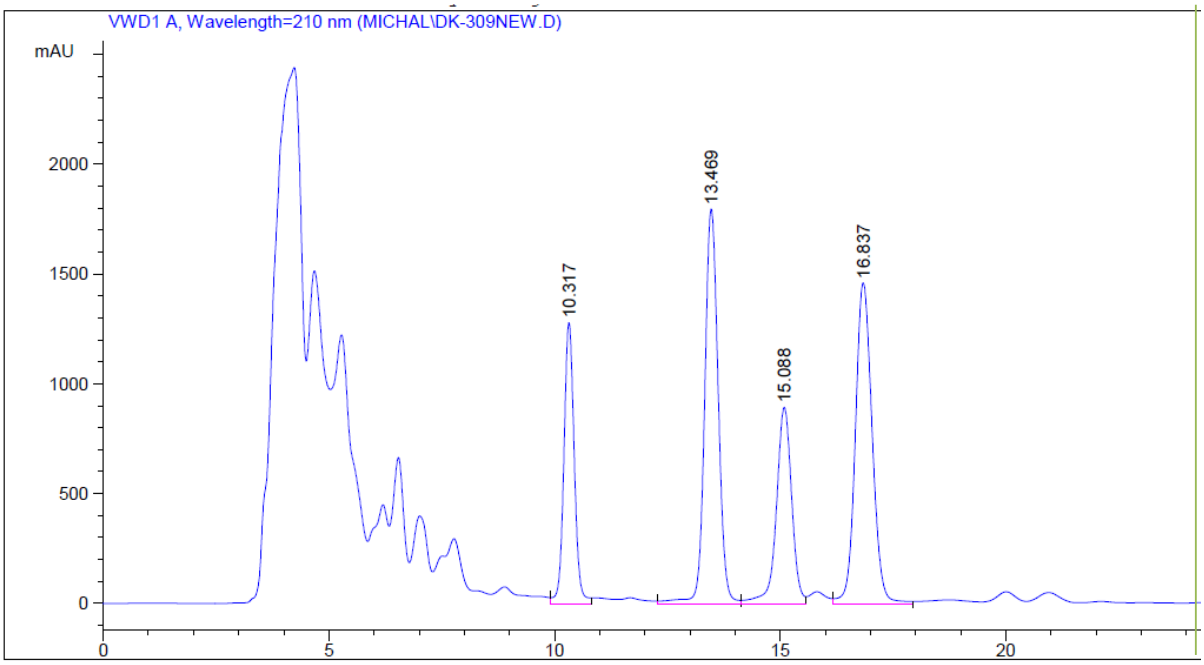

Peak #	RetTime [min]	Type	Width [min]	Area mAU *s	Height [mAU]	Area %
1	10.317	VV	0.2451	2.03233e4	1278.85144	17.4373
2	13.469	VV	0.3228	3.73880e4	1796.75903	32.0787
3	15.088	VV	0.3612	2.10825e4	893.53259	18.0886
4	16.837	VB	0.4007	3.77572e4	1460.09802	32.3954

Figure S48. HPLC trace of **8f**

a) chiral

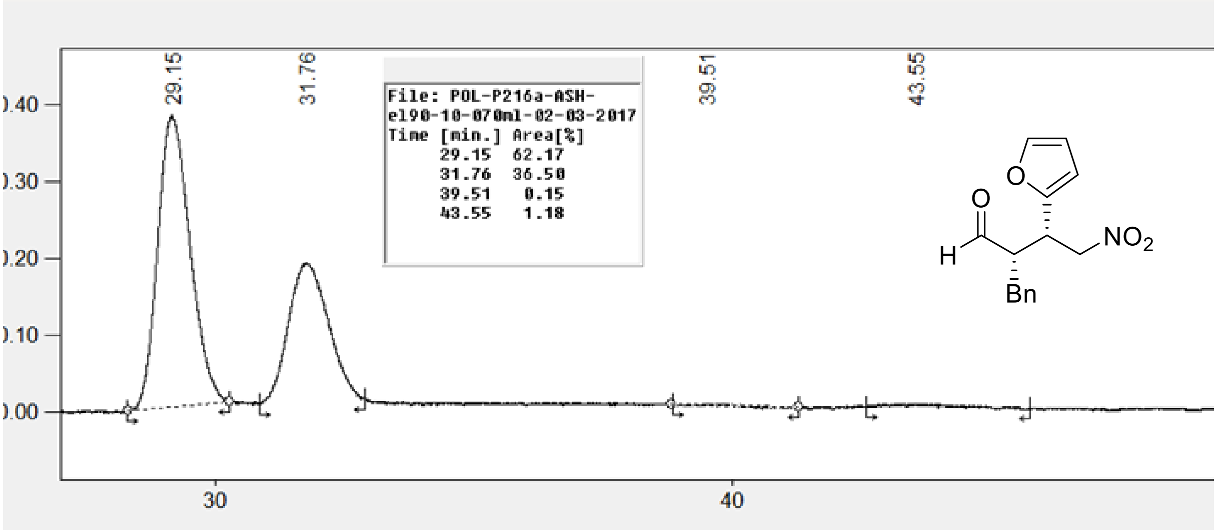

b) racemic

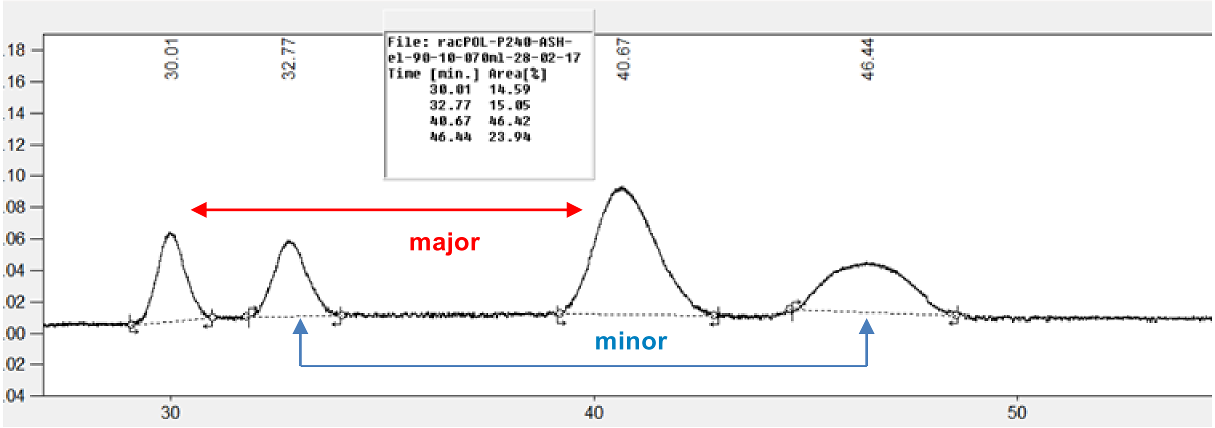

Figure S49. HPLC trace of **10a**

a) chiral

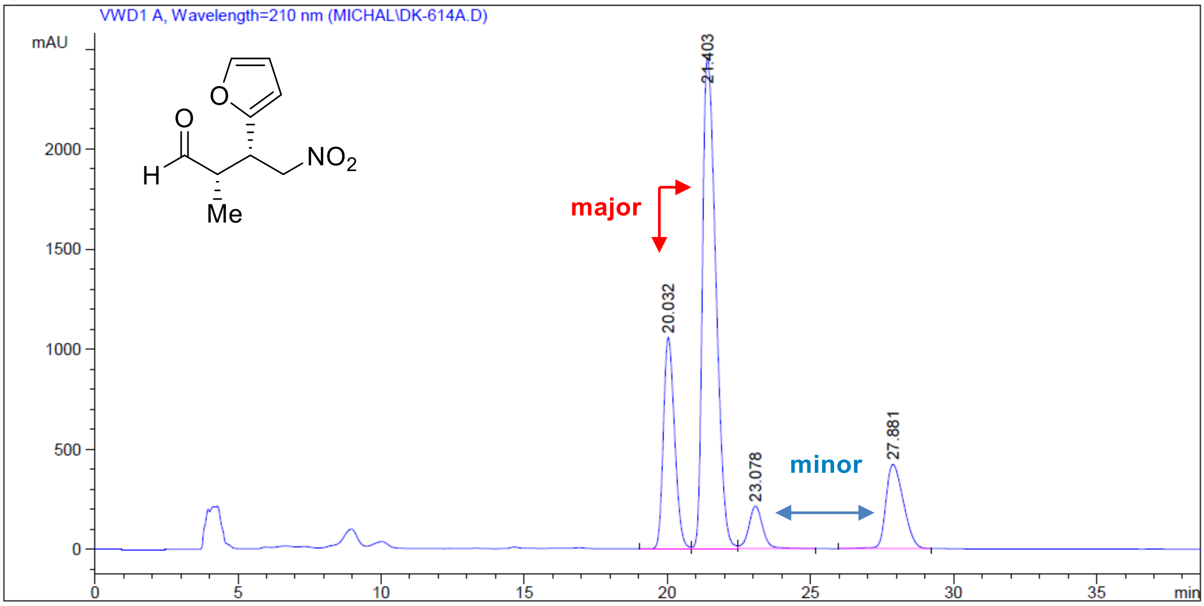

Peak #	RetTime [min]	Type	Width [min]	Area mAU *s	Height [mAU]	Area %
1	20.032	VV	0.4230	2.86672e4	1056.00574	21.5231
2	21.403	VV	0.5112	7.96291e4	2453.29492	59.7849
3	23.078	VB	0.5133	7095.70605	211.73839	5.3274
4	27.881	BB	0.6683	1.78006e4	419.53726	13.3646

b) racemic

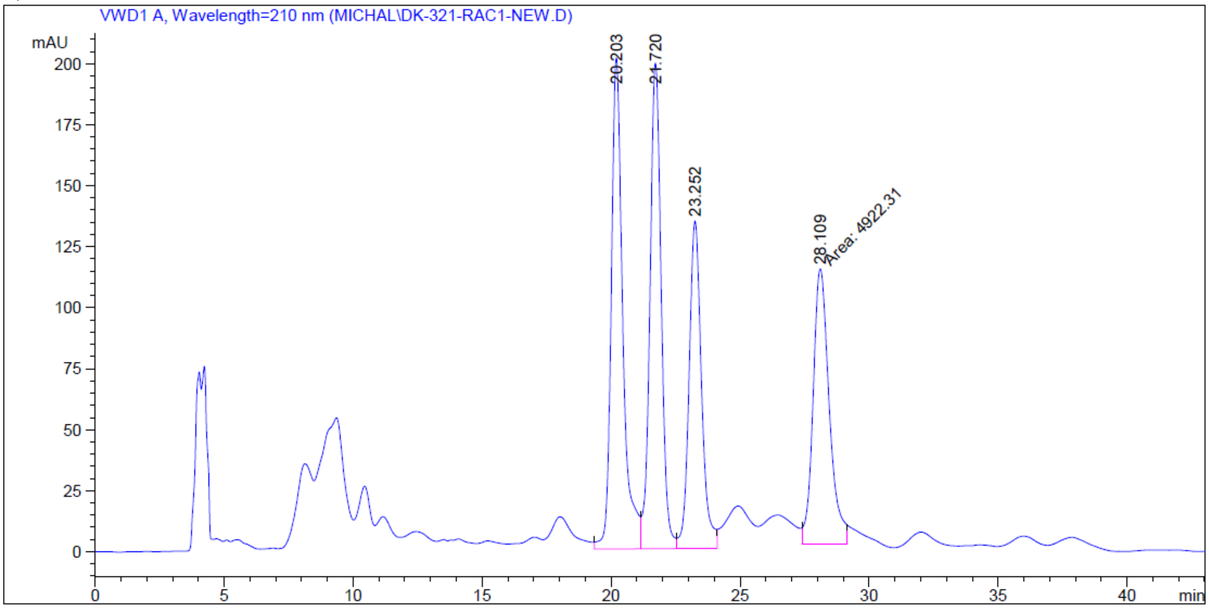

Peak #	RetTime [min]	Type	Width [min]	Area mAU *s	Height [mAU]	Area %
1	20.203	BV	0.4680	6264.87842	201.23669	28.8620
2	21.720	VV	0.4655	6027.56592	198.98976	27.7688
3	23.252	VB	0.5099	4491.53027	134.17992	20.6923
4	28.109	MM	0.7259	4922.31250	113.01044	22.6769

Figure S50. HPLC trace of **10b**

a) chiral

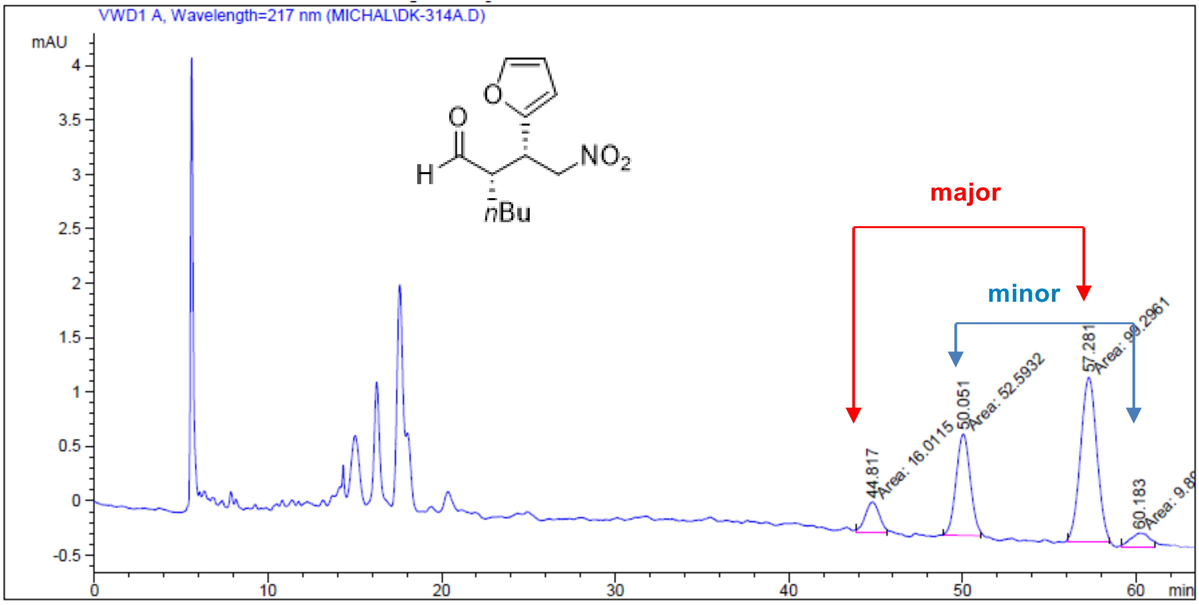

Peak #	RetTime [min]	Type	Width [min]	Area mAU *s	Height [mAU]	Area %
1	44.817	MM	0.9445	16.01153	2.82544e-1	9.0053
2	50.051	MM	0.9422	52.59317	9.30323e-1	29.5799
3	57.281	MM	1.0923	99.29606	1.51508	55.8469
4	60.183	MM	1.2547	9.89963	1.31496e-1	5.5678

b) racemic major

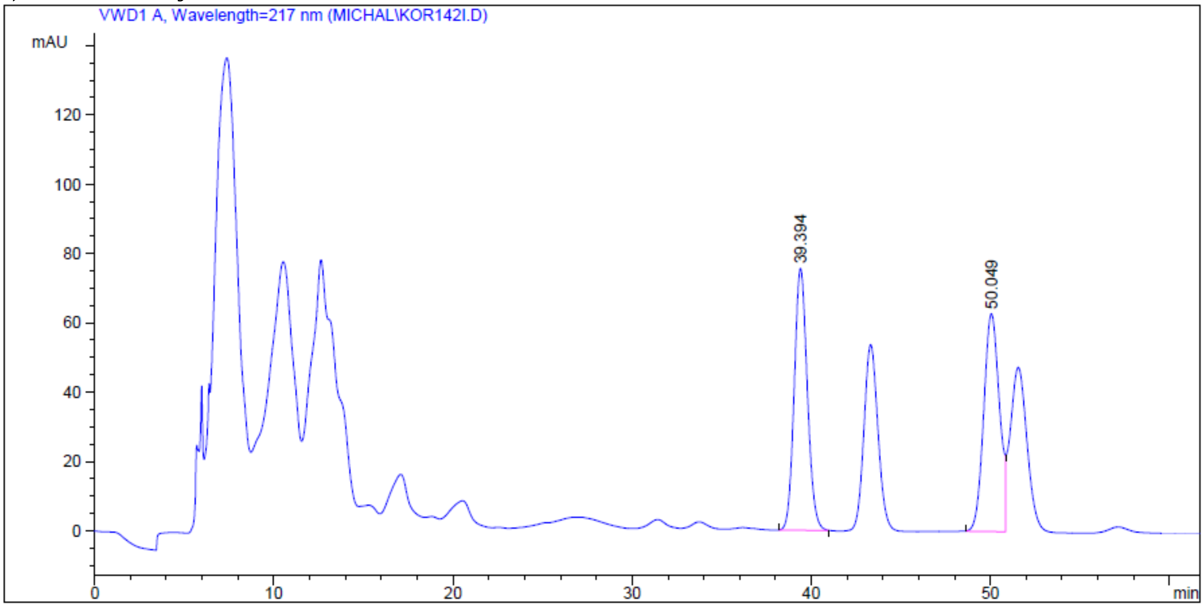

Peak #	RetTime [min]	Type	Width [min]	Area mAU *s	Height [mAU]	Area %
1	39.394	BB	0.7836	3840.16113	75.56295	50.0046
2	50.049	BV	0.9312	3839.45044	62.84376	49.9954

c) racemic minor

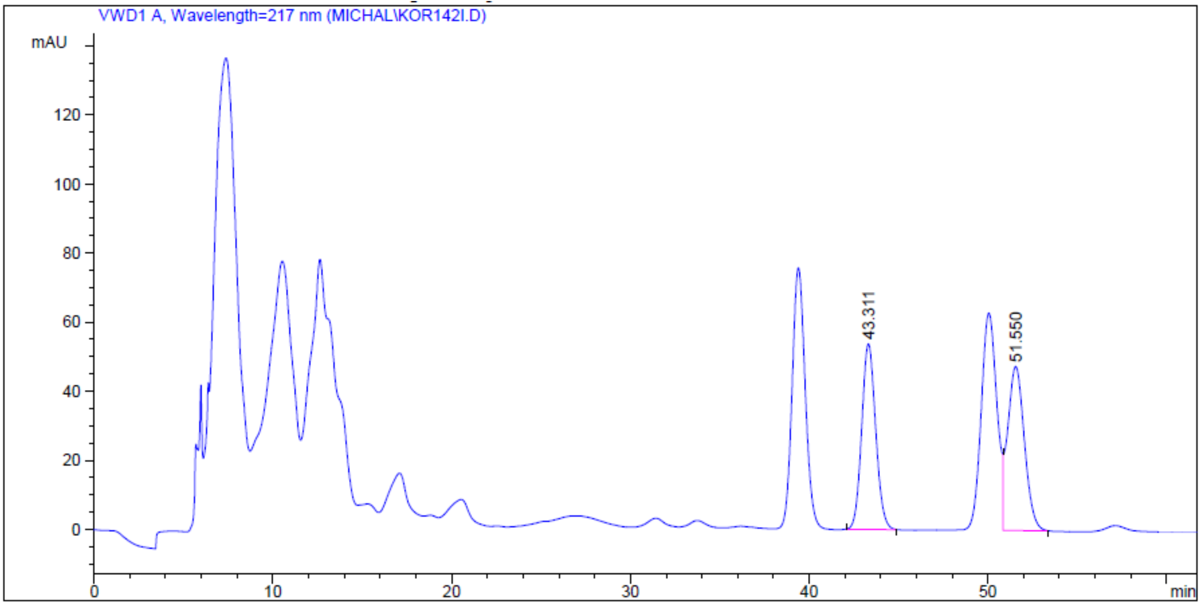

Peak #	RetTime [min]	Type	Width [min]	Area mAU *s	Height [mAU]	Area %
1	43.311	BB	0.8358	2924.79077	53.74328	48.5121
2	51.550	VB	0.9870	3104.19531	47.47612	51.4879

Figure S51. HPLC trace of **10c**

a) chiral

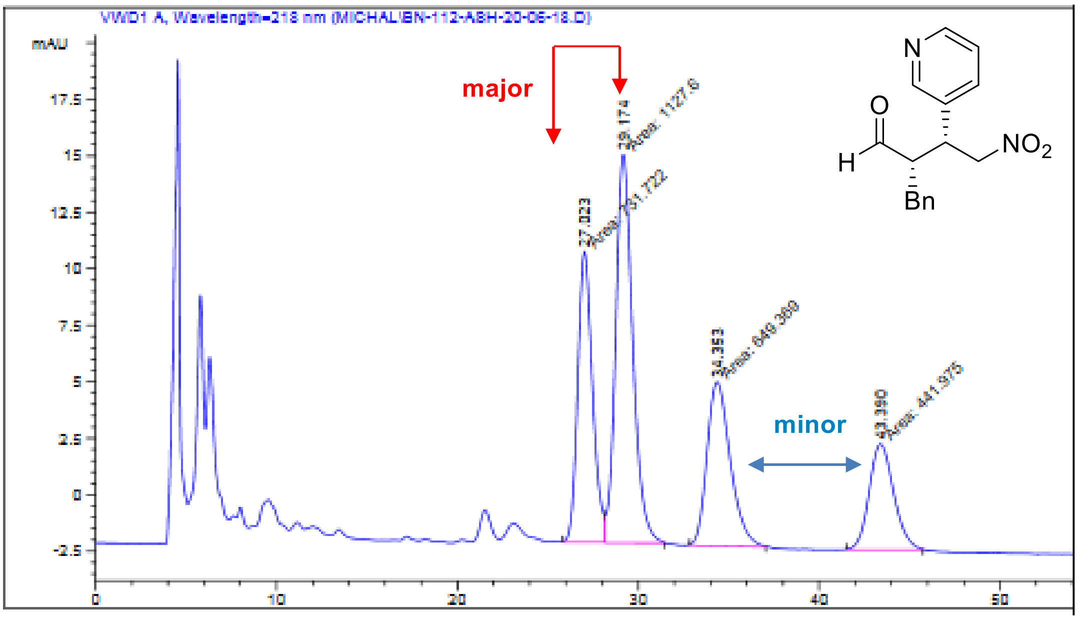

Peak #	RetTime [min]	Type	Width [min]	Area mAU *s	Height [mAU]	Area %
1	27.023	MM	0.9480	731.72192	12.86442	24.7985
2	29.174	MM	1.0935	1127.60132	17.18574	38.2151
3	34.353	MM	1.4947	649.36920	7.24085	22.0075
4	43.390	MM	1.5756	441.97504	4.67526	14.9788

b) racemic

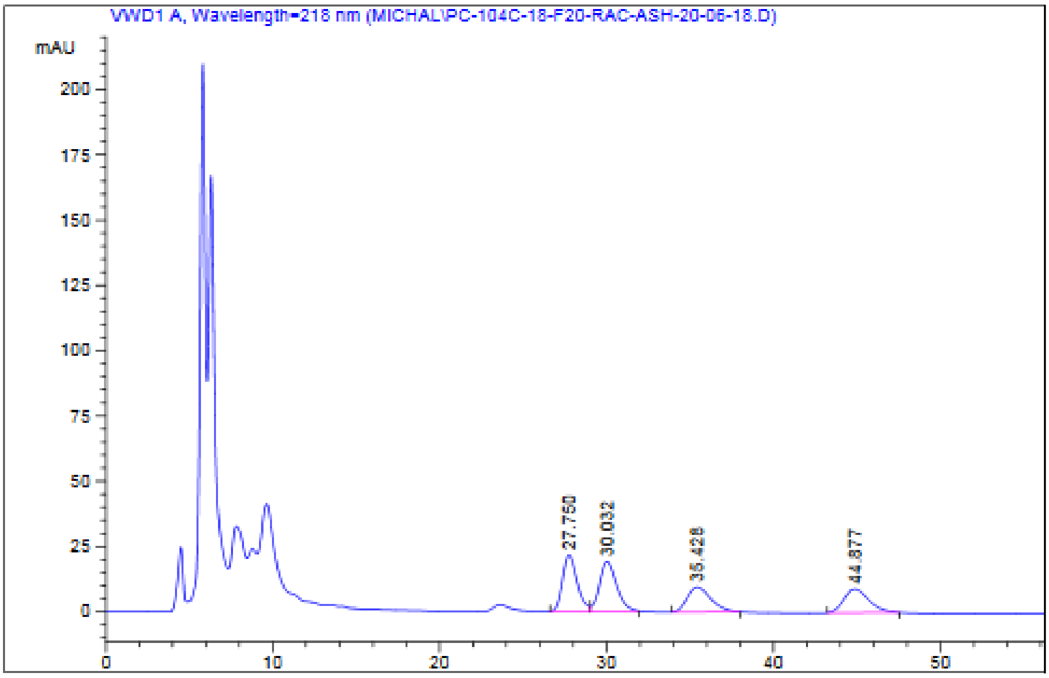

Peak #	RetTime [min]	Type	Width [min]	Area mAU *s	Height [mAU]	Area %
1	27.750	BV	0.9236	1311.46362	21.60843	28.8472
2	30.032	VB	1.0615	1350.06152	19.04457	29.6962
3	35.428	BB	1.3847	942.12134	9.54311	20.7231
4	44.877	BB	1.4174	942.60071	9.05108	20.7336

Figure S52. HPLC trace of **12**

5. Computational details

All calculations were realized using Turbomole program package [7,8]. Geometric optimizations were performed using PBEh-3c functional [9]. Geometrical optimizations were performed with def2-SV(P) basis set [10]. Energies were refined at M06-2X/def2-TZVP level [11,12].

3-phenylpropanal

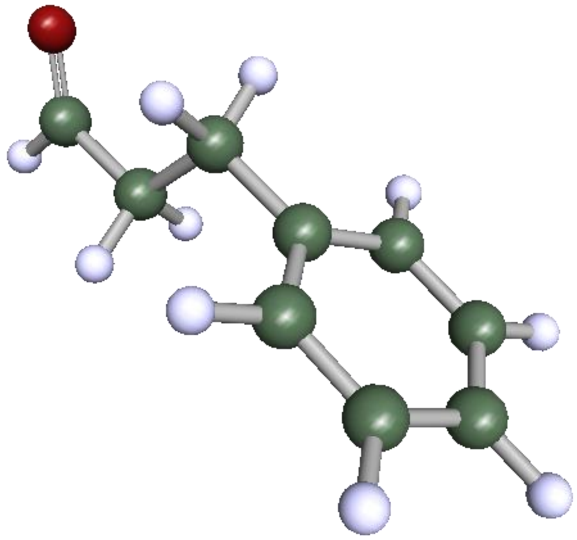

Geometry optimization, frequency calculation: PBEh-3c/def2-SV(P)

SFC energy	-422.9562016 Hartree
Chemical potential	0.134904 Hartree
Entropy	0.000152 Hartree/K
Inner energy	0.179399 Hartree
In(Qtrans)	17.9533
In(Qrot)	13.4997
In(Qvib)	6.1921
ZPE	0.170448 Hartree
Enthalpy	0.180343 Hartree

Single point energy: M06-2X/def2-TZVP

SCF energy	-424.16538665 Hartree

Cartesian coordinates

C	0.696695	-2.582738	-2.245463
H	1.641752	-2.766935	-2.818430
O	-0.237057	-3.317467	-2.361399
C	0.739445	-1.368415	-1.361337
C	-0.540423	-1.102125	-0.576966
C	-0.423104	0.124684	0.286059
C	-0.120195	2.422613	1.864623
C	0.069559	0.038291	1.587888
C	-0.763408	1.382895	-0.209735

C	-0.614458	2.522815	0.570018
C	0.220718	1.174893	2.371955
H	1.004899	-0.504923	-1.997132
H	1.605573	-1.481905	-0.685066
H	-0.777793	-1.979030	0.042751
H	0.333289	-0.938408	1.999165
H	0.603090	1.084380	3.389299
H	-0.005839	3.315665	2.479603
H	-0.891476	3.496574	0.164514
H	-1.159758	1.471953	-1.223441
H	-1.381509	-0.992817	-1.277006

Fur-nitroalkene

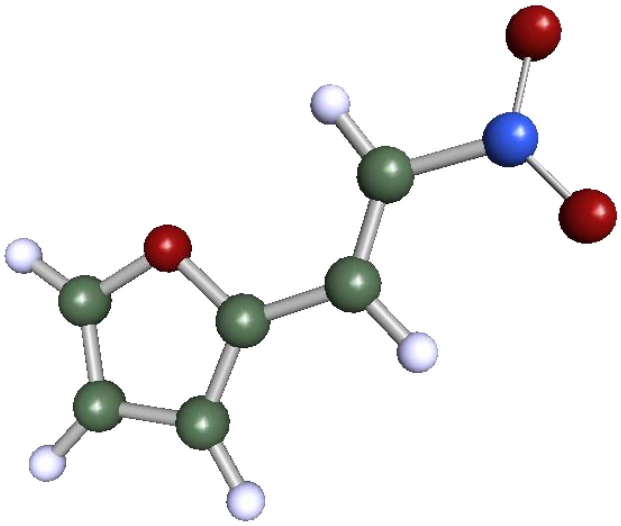

Geometry optimization, frequency calculation: PBEh-3c/def2-SV(P)

SFC energy	-510.4688093 Hartree
Chemical potential	0.076329 Hartree
Entropy	0.000145 Hartree/K
Inner energy	0.000145 Hartree
In(Qtrans)	18.0075
In(Qrot)	13.4168
In(Qvib)	4.8266
ZPE	0.110557 Hartree
Enthalpy	0.119502 Hartree

Single point energy: M06-2X/def2-TZVP

SCF energy	-511.93038572 Hartree

Cartesian coordinates

C	0.024362	-0.000521	2.529793
C	1.379421	0.000113	2.402070
C	1.629610	0.000559	1.005012
C	0.403233	0.000177	0.396201
O	-0.562521	-0.000397	1.333051
C	0.050339	0.000040	-0.991377
C	-1.200050	0.000236	-1.466395

N	-1.450095	-0.000149	-2.889652
O	-2.605807	0.001863	-3.220584
O	-0.516213	-0.002513	-3.646148
H	0.885046	-0.000423	-1.694537
H	-2.106685	0.000843	-0.865422
H	2.592721	0.001087	0.502814
H	2.107136	0.000253	3.207557
H	-0.630497	-0.001166	3.397518

SR-cat (*S*,*R*)-C2

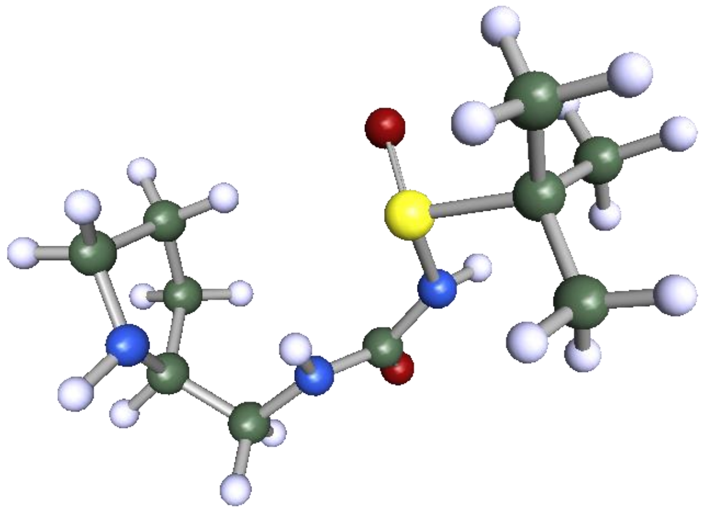

Geometry optimization, frequency calculation: PBEh-3c/def2-SV(P)

SFC energy	-1104.1083831 Hartree
Chemical potential	0.282091 Hartree
Entropy	0.000218 Hartree/K
Inner energy	0.346062 Hartree
In(Qtrans)	18.8708
In(Qrot)	15.1125
In(Qvib)	15.2198
ZPE	0.328548 Hartree
Enthalpy	0.347007 Hartree

Single point energy: M06-2X/def2-TZVP

SCF energy	-1106.5700552 Hartree

Cartesian coordinates

N	0.397207	-1.097265	-3.018100
C	-0.664397	-2.073872	-2.890237
C	-1.801576	-1.246193	-2.309859
C	-1.659285	0.085458	-3.050828
C	-0.158704	0.195739	-3.403136
C	0.569894	1.316904	-2.661873
N	0.551866	1.079299	-1.243943
C	-0.080197	1.885573	-0.362784
O	-0.531517	2.973878	-0.615312
N	-0.167448	1.384791	0.948673

S	-0.042596	-0.252676	1.339535
O	-1.390247	-0.835674	1.542468
C	0.713068	-0.075989	3.024073
C	-0.213641	0.714098	3.935688
C	0.856879	-1.514708	3.518277
C	2.074556	0.583033	2.855930
H	-1.666187	-1.117033	-1.226420
H	-2.784995	-1.713654	-2.453999
H	-0.363488	-2.904857	-2.234339
H	-0.970463	-2.519620	-3.859950
H	-2.255480	0.073039	-3.974450
H	-2.018011	0.940956	-2.461446
H	1.170487	-1.397962	-3.596733
H	0.107462	2.289362	-2.873226
H	1.612828	1.377408	-3.018126
H	-0.751650	1.964868	1.545220
H	-0.045773	0.393015	-4.486691
H	0.834441	0.143889	-0.977403
H	1.339497	-1.516204	4.506684
H	2.708317	0.034800	2.142572
H	2.601954	0.591137	3.821551
H	1.484818	-2.118404	2.845448
H	1.992790	1.621398	2.509101
H	-1.232936	0.303696	3.927847
H	-0.258063	1.779910	3.669058
H	0.157214	0.664674	4.970423
H	-0.116624	-2.012817	3.616406

SS-cat (*S*,*S*)-C2

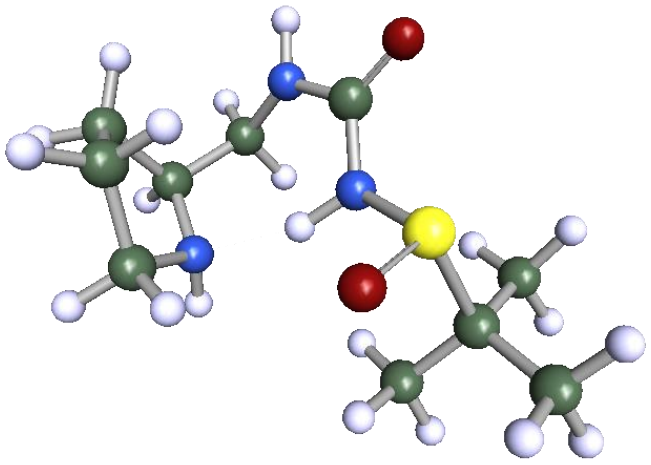

Geometry optimization, frequency calculation: PBEh-3c/Def2-SV(P)

SFC energy	-1104.1113906 Hartree
Chemical potential	0.283467 Hartree
Entropy	0.000213 Hartree/K
Inner energy	0.346045 Hartree
In(Qtrans)	18.8708
In(Qrot)	14.9653
In(Qvib)	14.2446

ZPE	0.328864 Hartree
Enthalpy	0.346989 Hartree

Single point energy: M06-2X/Def2-TZVP

SCF energy	-1106.57049853 Hartree

Cartesian coordinates

N	-0.929029	-1.612729	-0.631846
C	-2.275210	-1.113070	-0.886722
C	-2.151326	-0.523752	-2.282934
C	-1.231136	-1.518255	-2.989492
C	-0.286365	-2.024812	-1.884607
C	1.149108	-1.488920	-1.987478
N	1.290783	-0.084827	-2.290894
C	1.185304	0.988399	-1.442085
O	1.565297	2.089288	-1.750518
N	0.612989	0.687259	-0.217974
S	0.079664	1.968359	0.752927
O	-1.404385	1.886619	0.823968
C	0.700296	1.335397	2.384737
C	2.219752	1.291091	2.309201
C	0.233007	2.382783	3.393905
C	0.093556	-0.022379	2.697320
H	-1.694357	0.474730	-2.227267
H	-3.122898	-0.407990	-2.781043
H	-2.561365	-0.362495	-0.135991
H	-3.032086	-1.920720	-0.871074
H	-1.819028	-2.357107	-3.390480
H	-0.692083	-1.071770	-3.836054
H	-0.888681	-2.317826	0.092809
H	1.657079	-2.036153	-2.794147
H	1.693550	-1.750213	-1.062203
H	0.039410	-0.187425	-0.163925
H	-0.202906	-3.125292	-1.933247
H	1.739897	0.155474	-3.161524
H	0.588017	2.114468	4.400269
H	-1.003224	0.006350	2.634989
H	0.362889	-0.324797	3.721050
H	-0.862337	2.453548	3.429094
H	0.463383	-0.804580	2.019454
H	2.641206	2.257118	1.992628
H	2.573077	0.523638	1.607863
H	2.636384	1.058766	3.301246
H	0.631866	3.381725	3.159950

SR-cat starting materials complex

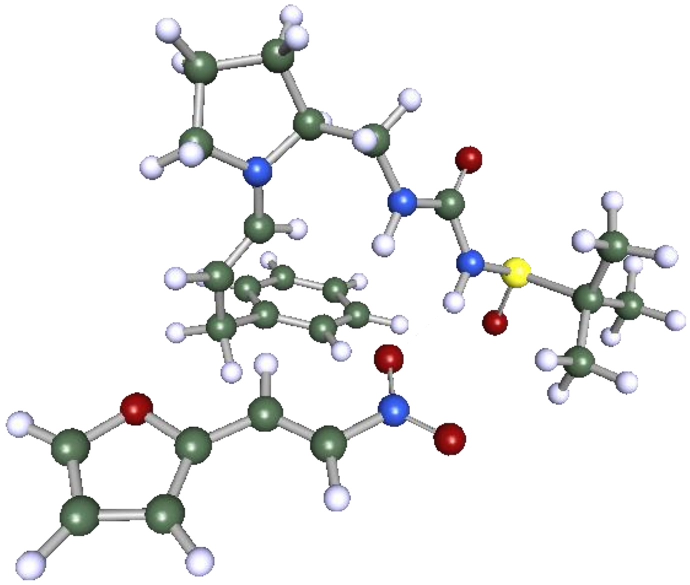

Geometry optimization, frequency calculation: PBEh-3c/def2-SV(P)

SFC energy	-1961.3374171 Hartree
Chemical potential	0.508668 Hartree
Entropy	0.000381 Hartree/K
Inner energy	0.621195 Hartree
In(Qtrans)	19.9343
In(Qrot)	17.4328
In(Qvib)	43.5825
ZPE	0.585100 Hartree
Enthalpy	0.622139 Hartree

Single point energy: M06-2X/def2-TZVP

SCF energy	-1966.24678927 Hartree

Cartesian coordinates

N	-2.251282	-0.138096	-1.989899
C	-3.508409	0.440351	-2.386599
C	-3.617368	0.052240	-3.858006
C	-2.167941	0.082962	-4.334947
C	-1.332732	-0.301025	-3.101868
C	-0.088315	0.586793	-2.994418
N	0.725108	0.354998	-1.834499
C	1.873764	-0.375020	-1.873546
O	2.285254	-0.936779	-2.858377
N	2.562396	-0.384174	-0.670493
S	3.941911	-1.342092	-0.459882
O	3.844625	-1.956362	0.890197
C	5.216832	-0.000187	-0.291850
C	4.926497	0.848112	0.935260
C	6.534391	-0.757250	-0.135256
C	5.204080	0.811201	-1.579862
C	-2.037359	-0.722819	-0.775470
C	-2.761934	-0.564310	0.348002

C	-2.540020	-1.345142	1.619783
C	-4.954182	2.619513	3.171729
C	-4.539144	3.651447	3.961058
C	-3.140537	3.741262	3.764821
C	-2.811383	2.755342	2.868914
O	-3.928253	2.085784	2.520239
C	-1.579454	2.326242	2.277386
C	-0.392552	2.892628	2.540717
N	0.810249	2.419039	1.922089
O	1.820662	3.013888	2.173433
O	0.750331	1.457826	1.189000
H	-0.223975	3.731325	3.212563
H	-1.636917	1.488869	1.571174
H	-2.457562	4.446808	4.229088
H	-5.932589	2.180241	2.993999
H	-5.159660	4.268270	4.603664
H	4.863593	0.233573	1.843472
H	5.737643	1.578277	1.080056
H	6.537015	-1.387933	0.763988
H	7.365658	-0.041763	-0.046765
H	6.068680	1.492319	-1.596378
H	6.743400	-1.398552	-1.005275
H	5.267983	0.171505	-2.472908
H	-3.508798	-1.739887	1.967400
H	-3.594867	0.144412	0.365720
H	-4.327921	0.026353	-1.778606
H	-4.028443	-0.965302	-3.938895
H	-4.274826	0.717078	-4.434801
H	-1.979301	-0.585371	-5.184977
H	-0.997454	-1.352294	-3.158634
H	-0.409136	1.641971	-2.990931
H	-1.901299	1.099111	-4.668640
H	-3.536911	1.539619	-2.252393
H	-2.205111	-0.672513	2.430491
H	-1.186528	-1.410392	-0.766616
H	2.215681	0.124507	0.139395
H	4.298624	1.425995	-1.670081
H	0.530024	0.440637	-3.890743
H	3.994219	1.423328	0.843929
H	0.417268	0.749746	-0.958221
C	-1.563926	-2.481114	1.482753
C	0.257002	-4.571664	1.083431
C	-0.191288	-2.266098	1.599531
C	-2.009360	-3.764008	1.167166
C	-1.108736	-4.803658	0.974311
C	0.717451	-3.298158	1.395766
H	0.178986	-1.267804	1.841698
H	-3.080656	-3.950212	1.066885
H	-1.477012	-5.801584	0.732369
H	0.967096	-5.383659	0.923568

H	1.786818	-3.098350	1.464888

SS-cat starting materials complex

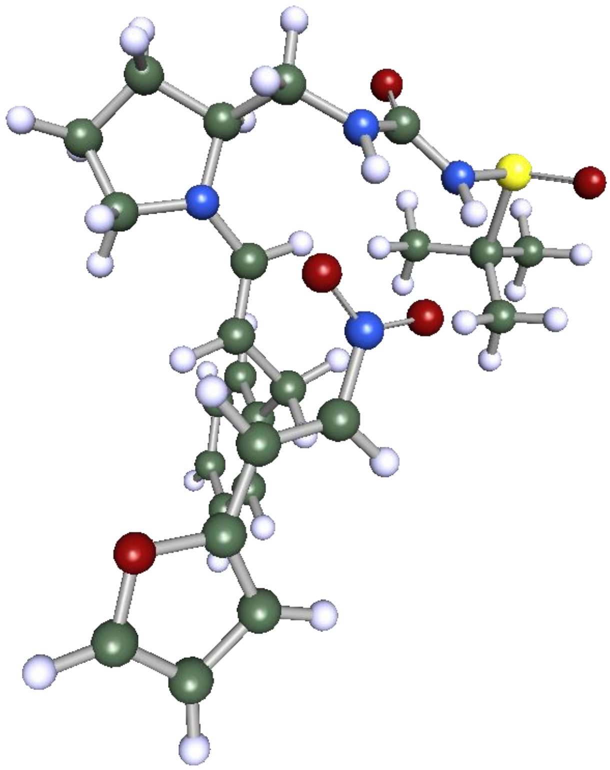

Geometry optimization, frequency calculation: PBEh-3c/Def2-SV(P)

SFC energy	-1961.3371845 Hartree
Chemical potential	0.509657 Hartree
Entropy	0.000374 Hartree/K
Inner energy	0.620248 Hartree
In(Qtrans)	19.9343
In(Qrot)	17.4656
In(Qvib)	42.4366
ZPE	0.585038 Hartree
Enthalpy	0.621192 Hartree

Single point energy: M06-2X/Def2-TZVP

SCF energy	-1966.24915538 Hartree

Cartesian coordinates

N	-1.281975	0.925343	-2.140187
C	-2.695916	1.190042	-2.237611
C	-2.984592	1.032066	-3.726369
C	-1.693672	1.508721	-4.381173
C	-0.584088	1.093273	-3.406192
C	0.530030	2.146321	-3.379450
N	1.592849	1.887065	-2.447181
C	2.500983	0.897999	-2.651072
O	2.599907	0.274662	-3.680523

N	3.290262	0.628464	-1.543881
S	4.641211	-0.397072	-1.659741
O	5.748295	0.273920	-0.938430
C	4.060051	-1.764299	-0.539656
C	2.824626	-2.386121	-1.175066
C	5.223300	-2.753824	-0.525773
C	3.788890	-1.223279	0.853917
C	-0.726400	0.303789	-1.064571
C	-1.279075	0.073537	0.142241
C	-0.586877	-0.727094	1.204101
C	-4.167139	2.896394	4.518361
C	-3.304793	2.301267	5.391032
C	-2.072536	2.202362	4.701087
C	-2.281507	2.747514	3.459659
O	-3.560249	3.162763	3.367080
C	-1.451455	2.934285	2.307869
C	-0.162616	2.577208	2.244349
N	0.579452	2.721991	1.028532
O	1.662194	2.186873	0.998631
O	0.109723	3.346892	0.115395
H	0.405342	2.104399	3.042221
H	-1.921438	3.379498	1.428162
H	-1.143922	1.779213	5.073295
H	-5.214734	3.175475	4.599050
H	-3.526904	1.973559	6.401854
H	3.005015	-2.688195	-2.217273
H	2.527893	-3.284340	-0.612363
H	5.460330	-3.120746	-1.536311
H	4.959332	-3.628695	0.087145
H	3.566428	-2.055471	1.539459
H	6.132770	-2.306818	-0.102524
H	4.659341	-0.683917	1.252499
H	-0.553197	-0.167394	2.153833
H	-2.290142	0.420617	0.371951
H	-3.265966	0.474138	-1.624674
H	-3.164429	-0.028564	-3.957937
H	-3.869583	1.592805	-4.056655
H	-1.536078	1.094234	-5.385542
H	-0.137919	0.132594	-3.719661
H	0.096515	3.120562	-3.111801
H	-1.708324	2.605748	-4.488915
H	-2.958456	2.202098	-1.874021
H	0.465711	-0.886930	0.915163
H	0.292460	-0.055960	-1.236745
H	3.213571	1.186955	-0.698012
H	2.921062	-0.548713	0.876213
H	0.939860	2.240952	-4.396711
H	1.967134	-1.699694	-1.170340
H	1.534586	2.340524	-1.547924
C	-1.233711	-2.066955	1.472940

C	-2.425616	-4.560065	1.952540
C	-1.333836	-3.018727	0.457300
C	-1.746035	-2.384683	2.727651
C	-2.339440	-3.619552	2.969067
C	-1.917986	-4.254428	0.693452
H	-0.950759	-2.781035	-0.537197
H	-1.681557	-1.650682	3.534093
H	-2.736811	-3.846213	3.959284
H	-2.888663	-5.529807	2.137789
H	-1.981126	-4.986848	-0.112436

TS-major-si-SR-cat

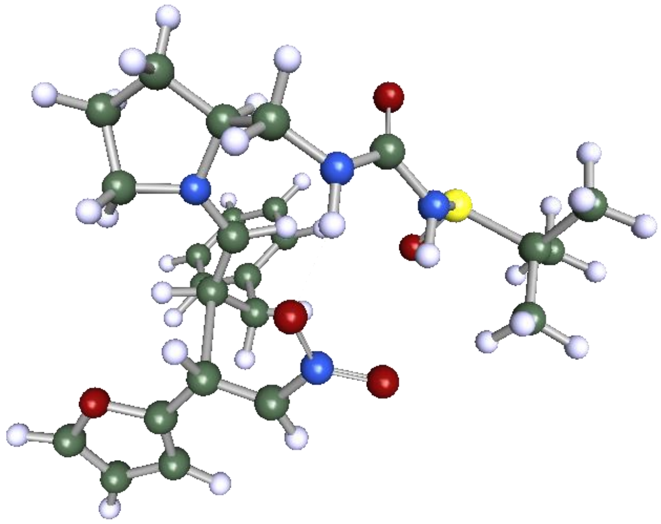

Geometry optimization, frequency calculation: PBEh-3c/def2-SV(P)

SFC energy	-1961.3235111 Hartree
Chemical potential	0.519363 Hartree
Entropy	0.000345 Hartree
Inner energy	0.621267 Hartree
In(Qtrans)	19.9343
In(Qrot)	17.2619
In(Qvib)	35.1157
ZPE	0.587639 Hartree
Enthalpy	0.622211

Single point energy: M06-2X/def2-TZVP

SCF energy	-1966.23666674 Hartree

Cartesian coordinates

N	-1.730925	0.372242	-1.807458
C	-3.172149	0.462988	-1.969327
C	-3.410901	-0.154080	-3.338959
C	-2.182951	0.281988	-4.127499
C	-1.039242	0.295853	-3.107363
C	-0.060560	1.441623	-3.396835
N	1.044079	1.551134	-2.491153
C	2.006608	0.601407	-2.437081

O	2.124226	-0.299206	-3.232270
N	2.861524	0.736484	-1.336834
S	3.506767	-0.699019	-0.711554
O	2.644301	-1.130998	0.424659
C	5.047242	0.003871	0.042019
C	4.717124	1.075905	1.068939
C	5.704571	-1.202288	0.712189
C	5.908286	0.529444	-1.098539
C	-1.128985	0.153482	-0.649545
C	-1.710979	0.075594	0.621630
C	-0.959488	-0.735596	1.653651
C	-4.804379	1.560181	3.155680
C	-4.040142	1.450306	4.273501
C	-2.695628	1.617529	3.837952
C	-2.750888	1.819363	2.487509
O	-4.035911	1.779971	2.084391
C	-1.736892	2.019942	1.466500
C	-0.437157	2.346012	1.869008
N	0.484224	2.666581	0.916476
O	1.664729	2.790318	1.205005
O	0.089510	2.795449	-0.252524
H	-0.039248	2.184180	2.866656
H	-2.103205	2.475783	0.546547
H	-1.800413	1.603193	4.451752
H	-5.877399	1.512459	2.989504
H	-4.391155	1.277183	5.286174
H	3.967575	0.729871	1.793491
H	5.627858	1.337976	1.628995
H	5.081344	-1.610894	1.518775
H	6.666700	-0.897884	1.149901
H	6.894186	0.825409	-0.709415
H	5.910912	-2.010503	-0.006088
H	6.075115	-0.234437	-1.872955
H	-1.260148	-0.416657	2.664311
H	-2.798389	-0.038984	0.645729
H	-3.689418	-0.072407	-1.162462
H	-3.449216	-1.250160	-3.251584
H	-4.356038	0.172767	-3.791561
H	-1.958762	-0.372963	-4.978885
H	-0.480470	-0.651894	-3.142808
H	-0.602779	2.397924	-3.373463
H	-2.335867	1.294660	-4.534980
H	-3.503456	1.514629	-1.928659
H	0.120261	-0.543684	1.567027
H	-0.054440	-0.031879	-0.717595
H	2.651516	1.460233	-0.647852
H	5.459917	1.407735	-1.580825
H	0.299324	1.301780	-4.428117
H	4.347140	2.003429	0.611956
H	0.901839	2.168161	-1.688118

C	-1.232044	-2.211893	1.500424
C	-1.808324	-4.928276	1.133981
C	-0.317731	-3.050739	0.864110
C	-2.433795	-2.754737	1.956992
C	-2.722437	-4.100893	1.776264
C	-0.605993	-4.398668	0.682474
H	0.637508	-2.646464	0.522621
H	-3.154181	-2.112543	2.469977
H	-3.664666	-4.508707	2.144911
H	-2.030663	-5.986578	0.992934
H	0.122733	-5.042203	0.187999

TS-major-re-SR-cat

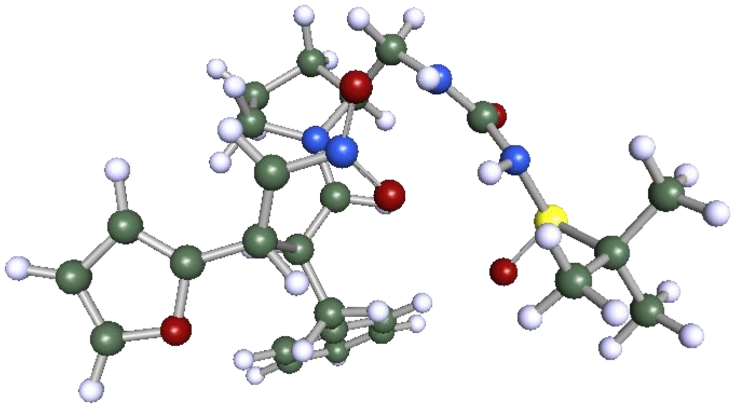

Geometry optimization, frequency calculation: PBEh-3c/def2-SV(P)

SFC energy	-1961.3240405 Hartree
Chemical potential	0.520006 Hartree
Entropy	0.000343 Hartree/K
Inner energy	0.621207 Hartree
In(Qtrans)	19.9343
In(Qrot)	17.2407
In(Qvib)	34.5162
ZPE	0.587696 Hartree
Enthalpy	0.622151 Hartree

Single point energy: M06-2X/def2-TZVP

SCF energy	-1966.24273958 Hartree

Cartesian coordinates

N	-1.168501	0.274731	-1.815880
C	-2.557560	0.542212	-2.162412
C	-2.756166	-0.288261	-3.421153
C	-1.430316	-0.097391	-4.154169
C	-0.375831	0.100752	-3.049147
C	0.551895	1.275762	-3.374320
N	1.529517	1.551753	-2.364847
C	2.444116	0.623495	-1.994583
O	2.697176	-0.367617	-2.637649

N	3.046879	0.895026	-0.766560
S	3.798506	-0.425253	-0.015040
O	2.784053	-1.149225	0.800638
C	4.826890	0.544145	1.187525
C	3.950967	1.383478	2.105854
C	5.559539	-0.534045	1.986025
C	5.810001	1.384960	0.384074
C	-0.762446	-0.187897	-0.642847
C	-1.539295	-0.323602	0.508931
C	-0.932716	-1.087179	1.665313
C	-4.935464	0.874468	2.848518
C	-5.517418	1.559823	1.830304
C	-4.446567	2.006628	1.005415
C	-3.296358	1.559589	1.593124
O	-3.606072	0.871934	2.706759
C	-1.890785	1.647073	1.225614
C	-1.521660	2.623461	0.295415
N	-0.201594	2.800272	0.009052
O	0.144088	3.636489	-0.824683
O	0.622290	2.075966	0.575503
H	-2.213376	3.225554	-0.286329
H	-4.521963	2.595223	0.096007
H	-5.337586	0.361905	3.718329
H	-6.579853	1.732254	1.687617
H	3.162173	0.779279	2.574453
H	4.568267	1.810381	2.911000
H	4.862773	-1.169842	2.548556
H	6.239978	-0.058069	2.707695
H	6.535208	1.856030	1.064821
H	6.168508	-1.182555	1.337703
H	6.380375	0.774035	-0.331782
H	-1.423715	-0.785305	2.602660
H	-2.606160	-0.500655	0.349346
H	-3.237650	0.286635	-1.341564
H	-2.909975	-1.343562	-3.149757
H	-3.625616	0.030865	-4.010828
H	-1.177019	-0.940398	-4.808997
H	0.236711	-0.802939	-2.932215
H	-0.042658	2.190741	-3.508135
H	-1.478651	0.797248	-4.794864
H	-2.689303	1.615466	-2.371436
H	0.129398	-0.819031	1.762376
H	0.294995	-0.462682	-0.589743
H	2.514432	1.511949	-0.147721
H	5.308028	2.183317	-0.177487
H	1.025866	1.056088	-4.345207
H	3.475260	2.228835	1.589619
H	1.383281	2.388348	-1.807948
C	-1.077910	-2.577755	1.484093
C	-1.400069	-5.325246	1.047942

C	-0.023459	-3.345155	0.989716
C	-2.291549	-3.207186	1.762562
C	-2.454607	-4.569009	1.546429
C	-0.185650	-4.708834	0.773697
H	0.938900	-2.871752	0.783898
H	-3.122469	-2.620762	2.162290
H	-3.409352	-5.044891	1.774554
H	-1.523752	-6.395744	0.880252
H	0.650844	-5.295458	0.391982
H	-1.181669	1.431993	2.027114

TS-minor-SR-cat

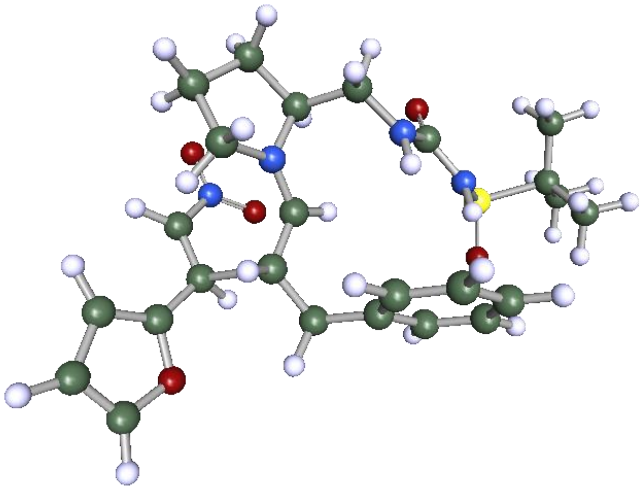

Geometry optimization, frequency calculation: PBEh-3c/Def2-SV(P)

SFC energy	-1961.3152305 Hartree
Chemical potential	0.517947 Hartree
Entropy	0.000348 Hartree/K
Inner energy	0.620700 Hartree
In(Qtrans)	19.9343
In(Qrot)	17.2754
In(Qvib)	35.8387
ZPE	0.586918 Hartree
Enthalpy	0.621644 Hartree

Single point energy: M06-2X/Def2-TZVP

SCF energy	-1966.23002198 Hartree

Cartesian coordinates

N	-1.787421	1.016079	-1.084338
C	-2.960392	1.857950	-0.914104
C	-3.267344	2.298688	-2.338472
C	-1.891694	2.393516	-3.011039
C	-0.947081	1.518515	-2.169580
C	0.231061	2.337732	-1.615697
N	1.119482	1.617073	-0.745779
C	2.112831	0.829200	-1.256596

O	2.353378	0.750722	-2.434625
N	2.843994	0.142018	-0.306444
S	3.813055	-1.185683	-0.779840
O	3.597040	-2.236461	0.245568
C	5.493132	-0.477592	-0.432746
C	5.625685	-0.135523	1.041058
C	6.452474	-1.599664	-0.826724
C	5.675695	0.737169	-1.331689
C	-1.422421	0.010976	-0.325402
C	-2.185838	-0.534083	0.721788
C	-1.416623	-1.445787	1.655639
C	-5.711073	-2.214039	2.519000
C	-6.531438	-1.320418	1.910599
C	-5.860201	-0.932448	0.714476
C	-4.685326	-1.626480	0.690556
O	-4.602656	-2.396025	1.790280
C	-3.551764	-1.648186	-0.239237
C	-3.815984	-1.191476	-1.546266
N	-2.854015	-1.308120	-2.514221
O	-3.062354	-0.879575	-3.641215
O	-1.777206	-1.819866	-2.196609
H	-4.728886	-0.685234	-1.846426
H	-2.926987	-2.542895	-0.161810
H	-6.216170	-0.235805	-0.038560
H	-5.792774	-2.785590	3.439515
H	-7.501361	-0.983868	2.264167
H	5.442359	-1.011682	1.677578
H	6.646890	0.219309	1.248727
H	6.299887	-2.501201	-0.217981
H	7.491167	-1.267504	-0.681676
H	6.721390	1.076863	-1.282066
H	6.342491	-1.877855	-1.885956
H	5.448620	0.511240	-2.383856
H	-1.094350	-2.346005	1.109042
H	-2.876539	0.156191	1.214502
H	-3.780963	1.312408	-0.438284
H	-3.875716	1.534455	-2.838626
H	-3.823820	3.244362	-2.367907
H	-1.928338	2.047936	-4.050100
H	-0.566430	0.657884	-2.739070
H	-0.157690	3.207914	-1.063480
H	-1.530939	3.433528	-3.029406
H	-2.708570	2.719347	-0.265920
H	-2.084096	-1.805197	2.452997
H	-0.480360	-0.464305	-0.596950
H	2.599816	0.170571	0.681446
H	5.039782	1.577940	-1.024591
H	0.799603	2.732987	-2.468953
H	4.936991	0.667644	1.341397
H	0.915058	1.591961	0.243665

C	-0.198875	-0.791734	2.270008
C	2.075692	0.419821	3.392902
C	-0.250243	0.515374	2.767096
C	1.014009	-1.473279	2.341188
C	2.139525	-0.879636	2.906496
C	0.872767	1.117853	3.318851
H	-1.186541	1.074398	2.725499
H	1.098741	-2.480146	1.931080
H	3.073432	-1.439451	2.939147
H	2.957537	0.887497	3.832367
H	0.806791	2.135789	3.705407

TS-minor-re-SR-cat

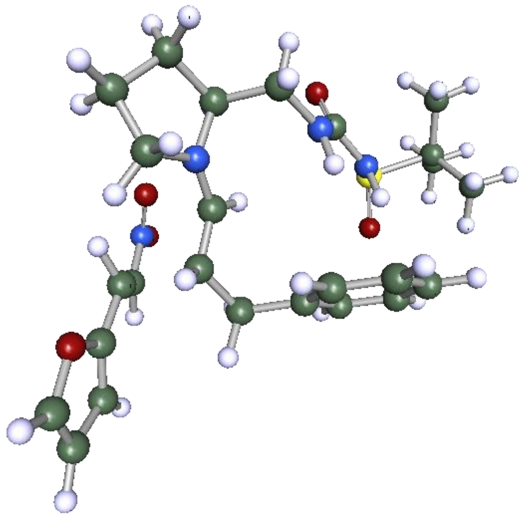

Geometry optimization, frequency calculation: PBEh-3c/def2-SV(P)

SFC energy	-1961.310462744 Hartree
Chemical potential	0.517148 Hartree
Entropy	0.000349 Hartree/K
Inner energy	0.620277 Hartree
In(Qtrans)	19.9343
In(Qrot)	17.2885
In(Qvib)	36.0136
ZPE	0.586297 Hartree
Enthalpy	0.621221 Hartree

Single point energy: M06-2X/def2-TZVP

SCF energy	-1966.223109725 Hartree

Cartesian coordinates

N	-1.948672	1.211631	-1.117280
C	-3.253957	1.840942	-1.051595
C	-3.455479	2.308215	-2.486823
C	-2.055244	2.757037	-2.905045
C	-1.093637	1.860861	-2.108080

C	0.018194	2.674710	-1.431473
N	0.918008	1.903672	-0.619026
C	1.977708	1.245769	-1.176758
O	2.245622	1.301075	-2.351019
N	2.740700	0.529244	-0.276609
S	3.830376	-0.664569	-0.833508
O	3.688813	-1.810770	0.098276
C	5.435037	0.155105	-0.386045
C	5.504892	0.387814	1.113220
C	6.497746	-0.844401	-0.839743
C	5.529243	1.450028	-1.181061
C	-1.590516	0.105269	-0.503729
C	-2.306648	-0.581336	0.496079
C	-1.465036	-1.478898	1.373237
C	-6.129512	-1.750862	1.973588
C	-5.712759	-3.004312	2.284467
C	-4.636146	-3.286322	1.393709
C	-4.488516	-2.181195	0.608170
O	-5.395602	-1.253055	0.969628
C	-3.561447	-1.822082	-0.465818
C	-2.866599	-2.867086	-1.095984
N	-2.102801	-2.588604	-2.202715
O	-1.513415	-3.480384	-2.779243
O	-2.024995	-1.405153	-2.562058
H	-2.802878	-3.885612	-0.723782
H	-3.948595	-1.044140	-1.128480
H	-4.052986	-4.200075	1.335971
H	-6.917903	-1.115608	2.368396
H	-6.124565	-3.653849	3.051010
H	5.371873	-0.546992	1.674322
H	6.490867	0.798783	1.379303
H	6.413795	-1.801076	-0.306756
H	7.499561	-0.435132	-0.642088
H	6.539238	1.874442	-1.073878
H	6.433700	-1.046469	-1.919840
H	5.347131	1.290995	-2.253915
H	-1.082984	-2.319061	0.769364
H	-3.055138	0.018789	1.022341
H	-4.022068	1.138655	-0.708116
H	-3.786769	1.458318	-3.100644
H	-4.209279	3.101560	-2.574867
H	-1.887841	2.672005	-3.985614
H	-0.643107	1.084018	-2.744178
H	-0.436742	3.451454	-0.796854
H	-1.897823	3.815357	-2.641094
H	-3.244183	2.691464	-0.345273
H	-2.109924	-1.937671	2.140350
H	-0.611997	-0.278399	-0.796851
H	2.481806	0.457082	0.705179
H	4.814465	2.205053	-0.827915

H	0.588989	3.195328	-2.213265
H	4.750317	1.112364	1.452453
H	0.689308	1.758676	0.355185
C	-0.300854	-0.789447	2.047508
C	1.871047	0.470256	3.314037
C	-0.428278	0.494606	2.588799
C	0.937267	-1.421449	2.145583
C	2.011035	-0.805573	2.782012
C	0.644184	1.121171	3.211456
H	-1.385725	1.014782	2.528248
H	1.081140	-2.407266	1.701674
H	2.966991	-1.325463	2.832217
H	2.712570	0.956042	3.809266
H	0.518701	2.120041	3.631776

TS-major-si-SS-cat

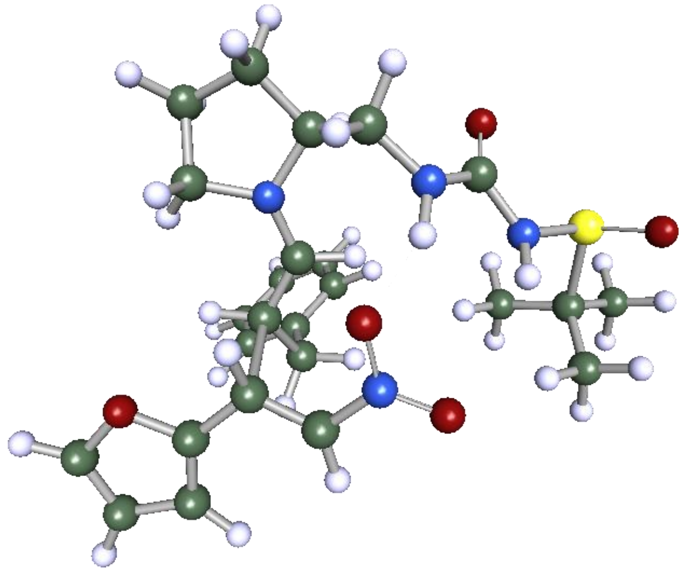

Geometry optimization, frequency calculation: PBEh-3c/def2-SV(P)

SFC energy	-1961.3180557 Hartree
Chemical potential	0.515745 Hartree
Entropy	0.000355 Hartree/K
Inner energy	0.620712 Hartree
In(Qtrans)	19.9343
In(Qrot)	17.3117
In(Qvib)	38.0181
ZPE	0.586809 Hartree
Enthalpy	0.621656 Hartree

Single point energy: M06-2X/def2-TZVP

SCF energy	-1966.22974711 Hartree

Cartesian coordinates

N	-1.238731	1.112187	-1.877240
C	-2.609441	1.494461	-2.179333

C	-2.732922	1.216065	-3.669917
C	-1.343480	1.550260	-4.195660
C	-0.385520	1.121573	-3.081281
C	0.831475	2.050443	-3.009143
N	1.814162	1.717537	-2.023425
C	2.640903	0.652384	-2.166505
O	2.676157	-0.050308	-3.148230
N	3.425178	0.415240	-1.042446
S	4.661867	-0.748489	-1.111721
O	5.970795	-0.067084	-1.005436
C	4.361517	-1.536366	0.547672
C	2.947803	-2.098582	0.549919
C	5.394247	-2.661246	0.603782
C	4.592694	-0.534187	1.667059
C	-0.843970	0.631650	-0.714819
C	-1.602721	0.485931	0.458827
C	-1.152870	-0.599912	1.410729
C	-4.723351	2.181298	2.799065
C	-4.167915	1.784984	3.973397
C	-2.764980	1.730680	3.736269
C	-2.580136	2.102681	2.434820
O	-3.775693	2.370709	1.874931
C	-1.402592	2.218845	1.586045
C	-0.143345	2.215881	2.205661
N	0.964796	2.456086	1.448395
O	2.084173	2.311562	1.911955
O	0.792572	2.796004	0.266939
H	0.048399	1.862936	3.214973
H	-1.530398	2.881141	0.728594
H	-1.989423	1.466991	4.448859
H	-5.749801	2.371224	2.496579
H	-4.691664	1.566143	4.898899
H	2.753261	-2.726298	-0.333545
H	2.793943	-2.727507	1.439708
H	5.262707	-3.380285	-0.219347
H	5.287293	-3.216219	1.547576
H	4.560364	-1.051823	2.638218
H	6.420299	-2.272351	0.556645
H	5.576931	-0.054665	1.577359
H	-1.523652	-0.379340	2.423733
H	-2.686998	0.555943	0.333274
H	-3.317774	0.913245	-1.573921
H	-2.966540	0.153472	-3.833742
H	-3.529875	1.803585	-4.143696
H	-1.106424	1.054925	-5.145702
H	-0.026451	0.094925	-3.256683
H	0.490769	3.076127	-2.805280
H	-1.255303	2.635113	-4.369680
H	-2.774711	2.559180	-1.943977
H	-0.053966	-0.599696	1.478499

H	0.190772	0.282608	-0.699877
H	3.474139	1.117540	-0.306574
H	3.822750	0.250266	1.704600
H	1.284975	2.055722	-4.012508
H	2.193998	-1.299580	0.576211
H	1.728234	2.195242	-1.124303
C	-1.646632	-1.961322	0.989632
C	-2.627364	-4.445003	0.137898
C	-0.902749	-2.763969	0.125111
C	-2.888688	-2.424430	1.421674
C	-3.377296	-3.654942	1.000110
C	-1.386340	-3.995438	-0.297431
H	0.076425	-2.425855	-0.220568
H	-3.481864	-1.812079	2.104709
H	-4.349168	-4.001814	1.353004
H	-3.006728	-5.412884	-0.190701
H	-0.785920	-4.610713	-0.968437

TS-major-re-SS-cat

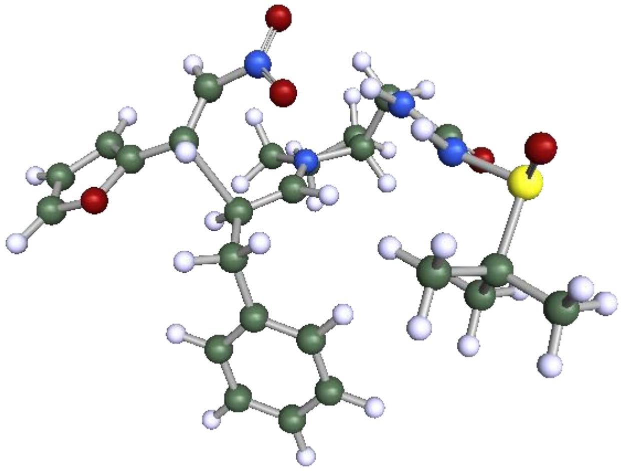

Geometry optimization, frequency calculation: PBEh-3c/def2-SV(P)

SFC energy	-1961.3162281 Hartree
Chemical potential	0.51645 Hartree
Entropy	0.000352 Hartree/K
Inner energy	0.620591 Hartree
In(Qtrans)	19.9343
In(Qrot)	17.3228
In(Qvib)	37.1549
ZPE	0.586710 Hartree
Enthalpy	0.621535 Hartree

Single point energy: M06-2X/def2-TZVP

SCF energy	-1966.23161921 Hartree

Cartesian coordinates

N	-0.889895	0.977455	-1.722546
C	-2.198286	1.180730	-2.339368

C	-1.988610	0.701798	-3.767195
C	-0.572921	1.177728	-4.063787
C	0.183356	1.005889	-2.743352
C	1.231471	2.103329	-2.550174
N	2.043702	1.961036	-1.377562
C	3.021396	1.026546	-1.303509
O	3.381791	0.343612	-2.235004
N	3.558128	0.872962	-0.034999
S	5.050566	0.069297	0.190997
O	5.760957	0.841131	1.237680
C	4.436981	-1.471953	1.027892
C	3.632920	-1.110552	2.264493
C	3.623473	-2.251466	0.004627
C	5.710419	-2.227876	1.400604
C	-0.699670	0.490731	-0.511185
C	-1.683311	0.269569	0.462502
C	-1.276924	-0.557615	1.663734
C	-5.361838	1.157932	2.521401
C	-5.872281	1.810932	1.445711
C	-4.748628	2.324063	0.737026
C	-3.642906	1.945173	1.443658
O	-4.026216	1.234804	2.519722
C	-2.209976	2.120335	1.222210
C	-1.825778	3.182756	0.392324
N	-0.502375	3.470221	0.233849
O	-0.152002	4.392239	-0.486775
O	0.321765	2.744427	0.813893
H	-2.509748	3.786511	-0.197048
H	-4.762842	2.907927	-0.178413
H	-5.821881	0.620941	3.346590
H	-6.922104	1.921196	1.191393
H	2.691276	-0.603261	2.009116
H	3.370386	-2.023052	2.821783
H	2.704139	-1.726285	-0.286051
H	3.330554	-3.224190	0.429159
H	5.447546	-3.193846	1.857349
H	4.194947	-2.446088	-0.914389
H	6.333008	-2.442481	0.518225
H	-1.966891	-0.360623	2.496643
H	-2.686458	0.058077	0.082249
H	-2.979796	0.631161	-1.799227
H	-2.052310	-0.396162	-3.812732
H	-2.737798	1.105074	-4.460848
H	-0.087497	0.628368	-4.880213
H	0.693012	0.029906	-2.724197
H	0.739891	3.084068	-2.501269
H	-0.585380	2.240812	-4.353658
H	-2.467518	2.246537	-2.314646
H	-0.283419	-0.229206	2.006112
H	0.342356	0.273477	-0.265742

H	3.291515	1.495861	0.722788
H	6.320995	-1.667896	2.121217
H	1.865763	2.101894	-3.449360
H	4.205104	-0.460382	2.940984
H	1.741617	2.461953	-0.545759
C	-1.263367	-2.035811	1.369358
C	-1.261595	-4.764843	0.726183
C	-0.072488	-2.703606	1.091693
C	-2.454956	-2.760398	1.327137
C	-2.456259	-4.111680	1.008213
C	-0.068469	-4.055746	0.771105
H	0.872882	-2.159824	1.135020
H	-3.398455	-2.258508	1.553366
H	-3.397620	-4.661975	0.984895
H	-1.261312	-5.826399	0.477844
H	0.876268	-4.557996	0.559125
H	-1.581824	1.930265	2.096011

TS-minor-si-SS-cat

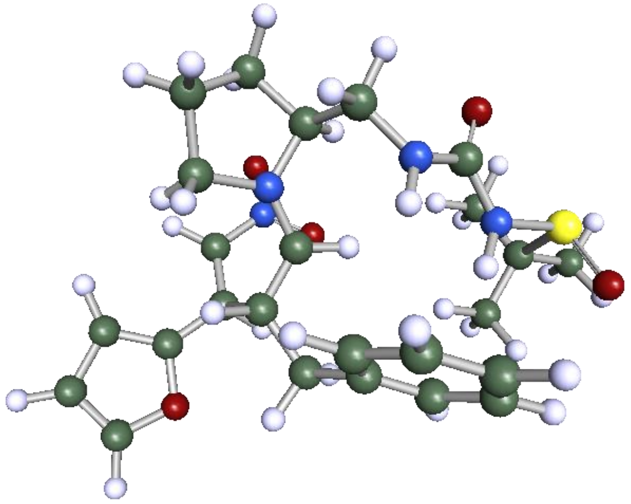

Geometry optimization, frequency calculation: PBEh-3c/Def2-SV(P)

SFC energy	-1961.3162133 Hartree
Chemical potential	0.519481 Hartree
Entropy	0.000343 Hartree/K
Inner energy	0.620676 Hartree
In(Qtrans)	19.9343
In(Qrot)	17.1698
In(Qvib)	34.3804
ZPE	0.586976 Hartree
Enthalpy	0.621620 Hartree

Single point energy: M06-2X/Def2-TZVP

SCF energy	-1966.23102434 Hartree

Cartesian coordinates

N	-1.295277	1.548393	-0.673200
C	-2.520487	2.303809	-0.404787
C	-2.477012	3.466275	-1.398005
C	-1.511283	2.994673	-2.484103
C	-0.482811	2.169407	-1.720369
C	0.653664	3.013595	-1.115610
N	1.586340	2.237313	-0.345023
C	2.535525	1.478656	-0.979996
O	2.879038	1.673110	-2.119056
N	3.038960	0.445204	-0.223368
S	4.459954	-0.387774	-0.691019
O	5.109403	-0.768656	0.589096
C	3.726678	-1.950004	-1.380585
C	4.953559	-2.790190	-1.734246
C	2.871112	-2.639605	-0.333897
C	2.930461	-1.595965	-2.628979
C	-0.891683	0.519619	0.031259
C	-1.677246	-0.142344	0.993734
C	-0.904358	-1.018151	1.962301
C	-5.136245	-2.251854	2.406520
C	-6.002155	-1.433325	1.756949
C	-5.282460	-0.918689	0.639434
C	-4.036264	-1.470906	0.697924
O	-3.956016	-2.272955	1.774725
C	-2.832661	-1.328814	-0.126974
C	-3.028559	-0.845624	-1.438917
N	-1.981920	-0.831780	-2.317516
O	-2.127875	-0.380277	-3.444094
O	-0.889226	-1.256374	-1.919284
H	-3.954274	-0.410571	-1.804599
H	-2.131425	-2.163473	-0.031866
H	-5.652514	-0.235004	-0.118525
H	-5.228034	-2.868379	3.296591
H	-7.031769	-1.226847	2.033122
H	5.550938	-3.042730	-0.847835
H	4.628909	-3.730615	-2.203500
H	3.416943	-2.779683	0.609656
H	2.571378	-3.633318	-0.699705
H	2.646176	-2.522940	-3.149445
H	1.945076	-2.081093	-0.137210
H	1.995846	-1.067285	-2.401397
H	-0.566193	-1.932985	1.449543
H	-2.469841	0.459556	1.447699
H	-3.398127	1.664291	-0.551761
H	-3.473487	3.703944	-1.790947
H	-2.105328	4.378415	-0.908945
H	-2.022602	2.345550	-3.208585
H	-0.049247	1.380014	-2.352603
H	0.246376	3.807930	-0.471738
H	-1.053647	3.820816	-3.044600

H	-2.534800	2.643376	0.644045
H	-1.577475	-1.361826	2.761530
H	0.093717	0.127645	-0.213387
H	2.723016	0.257904	0.726011
H	3.510682	-0.982313	-3.332523
H	1.187762	3.510818	-1.936188
H	5.607735	-2.275923	-2.455193
H	1.387471	2.071461	0.632250
C	0.303835	-0.333650	2.563728
C	2.595058	0.952036	3.567830
C	0.255195	0.999024	2.983705
C	1.517163	-1.010739	2.680495
C	2.655754	-0.378127	3.172363
C	1.385925	1.635044	3.482795
H	-0.682189	1.554395	2.917950
H	1.582411	-2.051906	2.361458
H	3.601341	-0.918344	3.211345
H	3.488075	1.454220	3.939508
H	1.323111	2.674845	3.806416

TS-minor-si-SS-cat

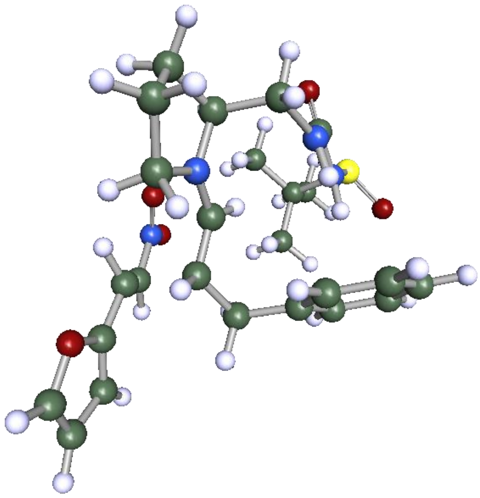

Geometry optimization, frequency calculation: PBEh-3c/Def2-SV(P)

SFC energy	-1961.3167839 Hartree
Chemical potential	0.51997 Hartree
Entropy	0.00034 Hartree/K
Inner energy	0.620485 Hartree
In(Qtrans)	19.9343
In(Qrot)	17.1456
In(Qvib)	33.7004
ZPE	0.5868 Hartree
Enthalpy	0.621429 Hartree

Single point energy: M06-2X/Def2-TZVP

SCF energy	-1966.229824173 Hartree

Cartesian coordinates

N	-1.334573	1.788056	-0.793096
C	-2.622127	2.448197	-0.626418
C	-2.491644	3.736198	-1.435874
C	-1.421342	3.404234	-2.474530
C	-0.453440	2.498640	-1.719113
C	0.637237	3.279391	-0.962535
N	1.504313	2.431241	-0.193908
C	2.480187	1.699664	-0.822656
O	2.887134	1.962898	-1.927048
N	2.922099	0.613718	-0.106265
S	4.329360	-0.255849	-0.553206
O	4.902971	-0.719893	0.735694
C	3.558457	-1.749151	-1.345994
C	4.759770	-2.624481	-1.701566
C	2.640269	-2.452299	-0.364109
C	2.826001	-1.296355	-2.600674
C	-0.991825	0.672243	-0.188325
C	-1.783831	-0.056432	0.719469
C	-1.022819	-0.907248	1.710896
C	-5.733439	-1.411474	1.541475
C	-5.305884	-2.633184	1.949357
C	-4.082461	-2.865854	1.256197
C	-3.867368	-1.766112	0.478059
O	-4.871357	-0.887305	0.660682
C	-2.794266	-1.372612	-0.434587
C	-1.942038	-2.387626	-0.901856
N	-1.017959	-2.101728	-1.874121
O	-0.273821	-2.977290	-2.280879
O	-0.957068	-0.939632	-2.294934
H	-1.884750	-3.387808	-0.482313
H	-3.098153	-0.625407	-1.171200
H	-3.449601	-3.745558	1.318806
H	-6.610818	-0.817661	1.783493
H	-5.803329	-3.293559	2.653217
H	5.323595	-2.934400	-0.811234
H	4.407813	-3.532169	-2.213174
H	3.162781	-2.708012	0.568478
H	2.258022	-3.379277	-0.814722
H	2.500959	-2.179503	-3.168777
H	1.759460	-1.839696	-0.125782
H	1.911968	-0.732451	-2.377551
H	-0.590348	-1.776530	1.186171
H	-2.638625	0.488137	1.130850
H	-3.429687	1.803104	-1.005904
H	-3.446391	4.039264	-1.883767
H	-2.167153	4.566194	-0.790989
H	-1.859879	2.854841	-3.320191
H	0.030781	1.771171	-2.387213

H	0.181511	4.016972	-0.284679
H	-0.927784	4.294134	-2.887918
H	-2.835584	2.632782	0.438627
H	-1.733630	-1.327518	2.440515
H	0.010980	0.310149	-0.414196
H	2.550997	0.382683	0.812953
H	3.463304	-0.681607	-3.252365
H	1.232313	3.841715	-1.695051
H	5.451720	-2.110870	-2.386899
H	1.239610	2.202269	0.754849
C	0.086375	-0.179728	2.437181
C	2.202327	1.179278	3.698577
C	-0.064050	1.139962	2.872895
C	1.310926	-0.806983	2.668276
C	2.363288	-0.137456	3.286084
C	0.979653	1.811863	3.499695
H	-1.013790	1.655922	2.719805
H	1.456776	-1.836453	2.338408
H	3.322316	-0.639880	3.410268
H	3.027906	1.710912	4.171471
H	0.837656	2.840920	3.832669

H-bonded product-major-SR-cat

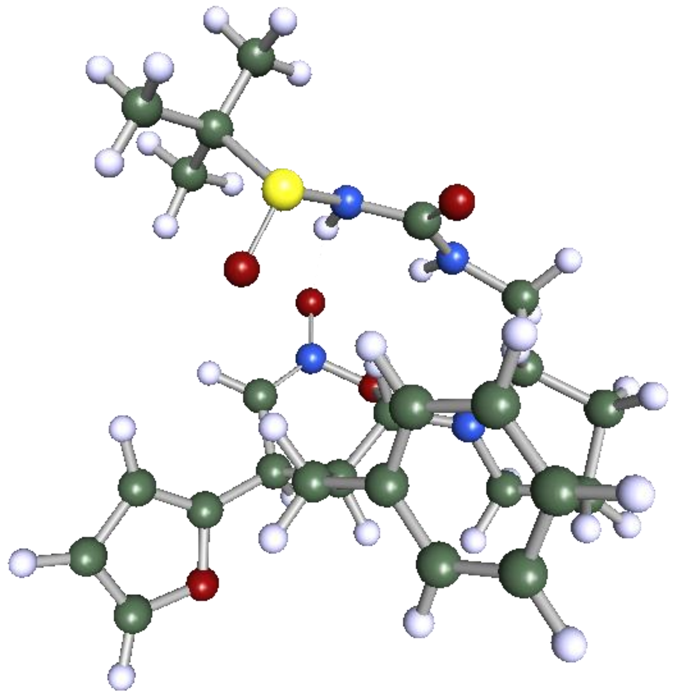

Geometry optimization, frequency calculation: PBEh-3c/Def2-SV(P)

SFC energy	-1961.3706396 Hartree
Chemical potential	0.525118 Hartree
Entropy	0.000337 Hartree/K
Inner energy	0.624621 Hartree
In(Qtrans)	19.9343
In(Qrot)	17.0800

In(Qvib)	33.8792
ZPE	0.592055 Hartree
Enthalpy	0.625565 Hartree

Single point energy: M06-2X/Def2-TZVP

SCF energy	-1966.28312483 Hartree

Cartesian coordinates

N	1.774257	-0.135299	-1.773382
C	3.149547	-0.359044	-2.149974
C	3.452717	0.846539	-3.028643
C	2.136551	1.072576	-3.765176
C	1.041509	0.619404	-2.786067
C	-0.060032	-0.137758	-3.545197
N	-1.140752	-0.643827	-2.735490
C	-1.955641	0.178874	-2.030409
O	-2.028993	1.371779	-2.193521
N	-2.704106	-0.487391	-1.050301
S	-3.154048	0.436050	0.291390
O	-2.115458	0.251732	1.349433
C	-4.616183	-0.568452	0.828589
C	-4.199124	-1.980772	1.207342
C	-5.141292	0.183444	2.051874
C	-5.635093	-0.540999	-0.302413
C	1.153537	-0.664220	-0.658427
C	1.905807	-0.618716	0.683752
C	1.537205	0.661063	1.449244
C	2.996678	-1.628887	4.752723
C	1.722543	-1.418938	5.164608
C	0.914729	-1.506659	3.989258
C	1.769256	-1.766143	2.964587
O	3.027685	-1.838927	3.427668
C	1.584065	-1.916884	1.494865
C	0.217266	-2.434924	1.184077
N	-0.126069	-2.545344	-0.053532
O	-1.167251	-3.001958	-0.537102
O	0.784785	-2.081587	-0.946933
H	-0.505850	-2.765604	1.925062
H	2.305967	-2.681371	1.151869
H	-0.159579	-1.364044	3.914391
H	3.947708	-1.664243	5.277084
H	1.392104	-1.224623	6.180462
H	-3.387601	-1.974249	1.947723
H	-5.055614	-2.507440	1.655196
H	-4.400512	0.213668	2.862362
H	-6.040666	-0.320198	2.435672
H	-6.581295	-0.984149	0.043110
H	-5.422626	1.218714	1.806524
H	-5.854115	0.486268	-0.630964
H	2.140153	0.709370	2.367174

H	2.990588	-0.621642	0.509272
H	3.811414	-0.407492	-1.273656
H	3.688510	1.712339	-2.393630
H	4.302003	0.682156	-3.705732
H	1.996020	2.115048	-4.078956
H	0.578843	1.495460	-2.305584
H	0.376307	-0.999735	-4.070088
H	2.105598	0.457367	-4.679132
H	3.291099	-1.306095	-2.706678
H	0.485863	0.602379	1.768715
H	0.193481	-0.143168	-0.509945
H	-2.430805	-1.454050	-0.841662
H	-5.295100	-1.109339	-1.177563
H	-0.455911	0.539052	-4.318873
H	-3.871448	-2.577151	0.344967
H	-1.093729	-1.617953	-2.471095
C	1.750772	1.923960	0.658333
C	2.150825	4.254785	-0.845708
C	0.668684	2.625706	0.130391
C	3.036973	2.418476	0.436172
C	3.238030	3.573066	-0.307514
C	0.865948	3.779227	-0.620798
H	-0.344584	2.263138	0.309555
H	3.897771	1.895256	0.859559
H	4.250356	3.947465	-0.465682
H	2.307082	5.161563	-1.430897
H	0.003241	4.303252	-1.032881

H-bonded product-minor-SR-cat

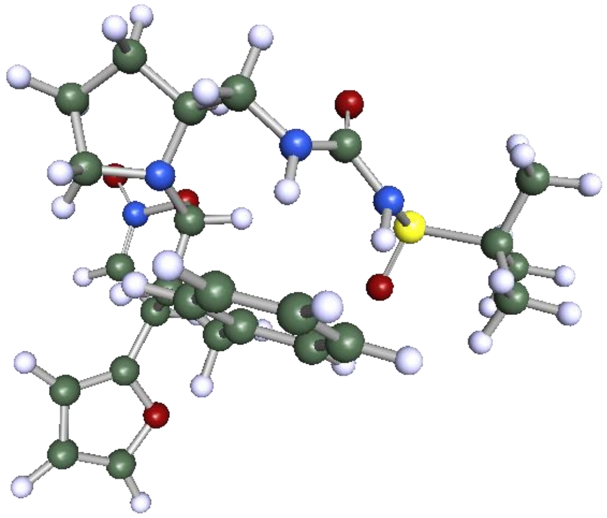

Geometry optimization, frequency calculation: PBEh-3c/Def2-SV(P)

SFC energy	-1961.3593467 Hartree
Chemical potential	0.523767 Hartree
Entropy	0.00034 Hartree
Inner energy	0.624072 Hartree
In(Qtrans)	19.9343

In(Qrot)	17.2079
In(Qvib)	34.2804
ZPE	0.591203 Hartree
Enthalpy	0.625017 Hartree

Single point energy: M06-2X/Def2-TZVP

SCF energy	-1966.27014037 Hartree

Cartesian coordinates

N	-1.616065	1.457813	-1.067225
C	-2.947891	2.049544	-0.990728
C	-3.162404	2.711351	-2.346808
C	-1.768672	3.227443	-2.678564
C	-0.813476	2.198592	-2.049833
C	0.392350	2.894999	-1.416514
N	1.288159	2.016402	-0.710578
C	2.100672	1.152231	-1.368911
O	2.241416	1.119880	-2.563832
N	2.789531	0.279321	-0.520028
S	3.096256	-1.295577	-1.068644
O	2.259456	-2.214960	-0.249042
C	4.830300	-1.477819	-0.439588
C	4.877594	-1.286016	1.067503
C	5.217437	-2.906253	-0.818309
C	5.684686	-0.461553	-1.184469
C	-1.433657	0.061073	-1.000985
C	-2.037956	-0.604612	0.255964
C	-0.972868	-0.958174	1.294163
C	-4.092551	-3.825407	2.565547
C	-5.075146	-2.892503	2.620429
C	-4.782678	-1.962692	1.576727
C	-3.646490	-2.410731	0.976743
O	-3.233195	-3.536833	1.578344
C	-2.828602	-1.874042	-0.150590
C	-3.700771	-1.550594	-1.320118
N	-3.187214	-0.918146	-2.329683
O	-3.705003	-0.584941	-3.373715
O	-1.846672	-0.645370	-2.212836
H	-4.755649	-1.805622	-1.390516
H	-2.098881	-2.654470	-0.436802
H	-5.349864	-1.076218	1.305887
H	-3.889477	-4.721266	3.145823
H	-5.910172	-2.868306	3.314111
H	4.176929	-1.958038	1.582711
H	5.888391	-1.514952	1.438359
H	4.581452	-3.648342	-0.317425
H	6.259197	-3.098082	-0.521609
H	6.746818	-0.625198	-0.946899
H	5.149737	-3.073727	-1.903943
H	5.571941	-0.553160	-2.275121

H	-0.261370	-1.671662	0.848455
H	-2.769610	0.078050	0.712587
H	-3.725792	1.310938	-0.757529
H	-3.484438	1.960730	-3.080760
H	-3.923167	3.503931	-2.320432
H	-1.596117	3.341115	-3.756709
H	-0.449989	1.510905	-2.828785
H	0.041883	3.646583	-0.693508
H	-1.611847	4.220450	-2.224801
H	-2.986247	2.805961	-0.183322
H	-1.464912	-1.503717	2.116457
H	-0.357327	-0.153091	-1.030359
H	2.618953	0.351262	0.480903
H	5.431974	0.570402	-0.907417
H	0.945501	3.435618	-2.199104
H	4.658187	-0.249151	1.361262
H	1.085196	1.858948	0.266345
C	-0.193452	0.173587	1.912943
C	1.320582	2.189300	3.163698
C	-0.721221	1.450377	2.107926
C	1.102449	-0.077470	2.374569
C	1.852624	0.915586	2.994798
C	0.028688	2.448177	2.722958
H	-1.728990	1.686476	1.769334
H	1.528959	-1.072343	2.232771
H	2.859951	0.691001	3.349025
H	1.906929	2.973350	3.642965
H	-0.404264	3.440142	2.856533

H-bonded-product-major-SS-cat

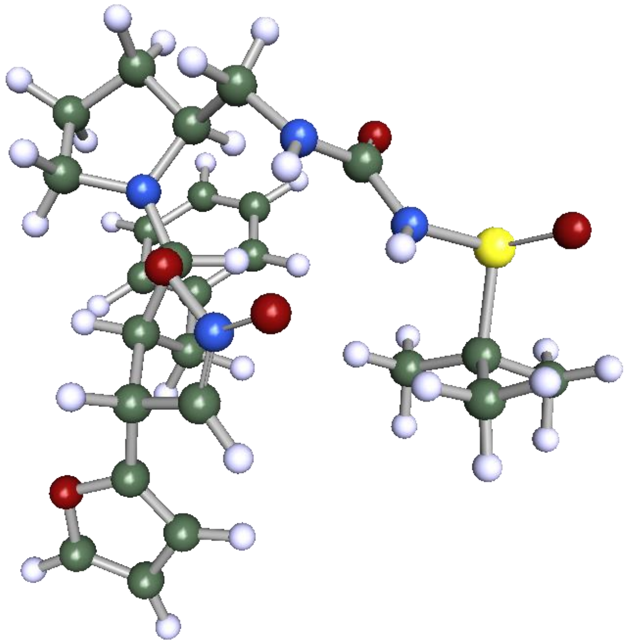

Geometry optimization, frequency calculation: PBEh-3c/Def2-SV(P)
SFC energy -1961.3635364 Hartree

Chemical potential	0.526896 Hartree
Entropy	0.000326 Hartree/K
Inner energy	0.623136 Hartree
In(Qtrans)	19.9343
In(Qrot)	17.1413
In(Qvib)	31.0628
ZPE	0.591231 Hartree
Enthalpy	0.624080 Hartree

Single point energy: M06-2X/Def2-TZVP

SCF energy -1966.27427535 Hartree

Cartesian coordinates

N	-1.295175	1.029443	-1.846941
C	-2.566786	1.573847	-2.263723
C	-2.906233	0.724989	-3.480299
C	-1.544986	0.507768	-4.130046
C	-0.550430	0.448706	-2.961923
C	0.760447	1.148797	-3.342713
N	1.791224	1.134502	-2.336819
C	2.418810	-0.007129	-1.956587
O	2.288071	-1.070910	-2.511994
N	3.204920	0.149971	-0.819200
S	4.283238	-1.073079	-0.353763
O	5.668064	-0.570951	-0.495999
C	3.921339	-1.041069	1.473128
C	2.455635	-1.399785	1.667576
C	4.827899	-2.130874	2.043227
C	4.273776	0.315269	2.063127
C	-0.750089	1.138371	-0.584851
C	-1.680296	0.911987	0.621262
C	-1.608668	-0.556902	1.062325
C	-3.326664	1.130972	4.579155
C	-2.174215	0.710429	5.154291
C	-1.149972	0.942996	4.184952
C	-1.768945	1.488071	3.104199
O	-3.085098	1.600308	3.345074
C	-1.302272	1.914300	1.756068
C	0.163786	2.201741	1.756890
N	0.721426	2.520469	0.632983
O	1.879656	2.854971	0.407559
O	-0.106247	2.477490	-0.444777
H	0.797042	2.227143	2.640489
H	-1.823787	2.860101	1.515080
H	-0.092239	0.719094	4.286092
H	-4.356897	1.162898	4.922894
H	-2.060086	0.288423	6.148127
H	2.187752	-2.332477	1.147422
H	2.244477	-1.551037	2.737416
H	4.613895	-3.115340	1.599472

H	4.669068	-2.216991	3.128586
H	4.216685	0.268047	3.161773
H	5.889354	-1.904757	1.874952
H	5.297313	0.612495	1.796658
H	-2.308390	-0.717134	1.894946
H	-2.719763	1.133603	0.343433
H	-3.329941	1.486974	-1.477099
H	-3.331411	-0.234196	-3.150788
H	-3.632857	1.205313	-4.149571
H	-1.501405	-0.397793	-4.748703
H	-0.313704	-0.600130	-2.721985
H	0.560276	2.202348	-3.585866
H	-1.303001	1.357209	-4.789868
H	-2.504327	2.646946	-2.529357
H	-0.605337	-0.771120	1.463980
H	0.086517	0.422166	-0.508063
H	3.335679	1.078465	-0.424321
H	3.585490	1.110577	1.740875
H	1.133856	0.674014	-4.263585
H	1.789730	-0.604861	1.304808
H	1.922834	1.975277	-1.792690
C	-1.933072	-1.526136	-0.043747
C	-2.559438	-3.277239	-2.141578
C	-0.932935	-2.238291	-0.702107
C	-3.256473	-1.716845	-0.444662
C	-3.569349	-2.583114	-1.482643
C	-1.240327	-3.104087	-1.745870
H	0.111222	-2.113302	-0.409186
H	-4.058830	-1.183229	0.070176
H	-4.610115	-2.721919	-1.777820
H	-2.803471	-3.957966	-2.957604
H	-0.436150	-3.638227	-2.252344

H-bonded-product-major2-SS-cat

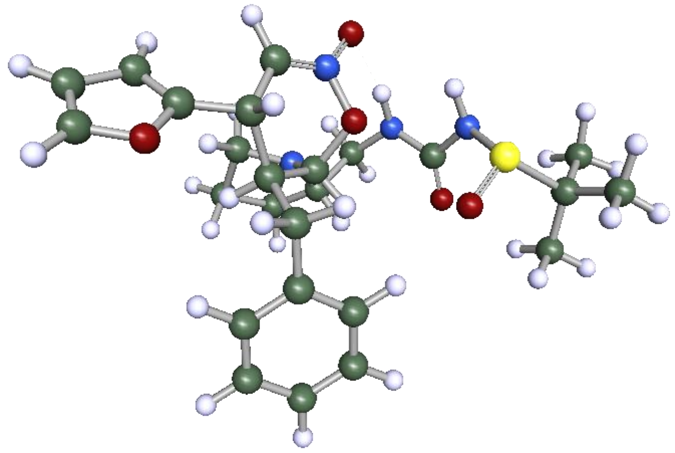

Geometry optimization, frequency calculation: PBEh-3c/def2-SV(P)

SFC energy	-1961.3558144 Hartree
Chemical potential	0.524979 Hartree
Entropy	0.000338 Hartree/K
Inner energy	0.624800 Hartree
In(Qtrans)	19.9343
In(Qrot)	17.1777
In(Qvib)	34.0836
ZPE	0.592201 Hartree
Enthalpy	0.625744 Hartree

Single point energy: M06-2X/def2-TZVP

SCF energy	-1966.26861183 Hartree

Cartesian coordinates

N	-1.325373	0.678216	-1.142194
C	-2.706558	1.042156	-1.369006
C	-3.198974	-0.045460	-2.318269
C	-1.984543	-0.291519	-3.215604
C	-0.761699	0.122511	-2.366185
C	0.128899	1.115661	-3.127857
N	1.168595	1.694651	-2.320180
C	2.205441	0.936784	-1.880865
O	2.477222	-0.150121	-2.327175
N	2.952718	1.543796	-0.866643
S	3.643997	0.700408	0.442181
O	2.779492	-0.427676	0.872882
C	5.197647	-0.049224	-0.271885
C	5.782564	0.886203	-1.318045
C	6.106257	-0.113182	0.960667
C	4.946027	-1.447127	-0.810906
C	-0.722310	0.432640	0.094951
C	-1.649678	0.185415	1.288578
C	-1.020154	-0.830618	2.255474
C	-5.207011	1.019547	3.438744
C	-5.615421	1.992420	2.589616
C	-4.445194	2.401412	1.876682
C	-3.423804	1.642505	2.357924
O	-3.888405	0.807927	3.301461
C	-1.975142	1.530841	2.018732
C	-1.525193	2.741084	1.262971
N	-0.433629	2.708191	0.571774
O	0.122619	3.617616	-0.035429
O	0.193808	1.508782	0.515233
H	-2.029814	3.703030	1.313698
H	-4.382344	3.161690	1.104080
H	-5.726074	0.414433	4.176986
H	-6.624140	2.379205	2.480992
H	5.857864	1.922782	-0.956602
H	6.801301	0.553329	-1.569977
H	6.334343	0.885614	1.361170

H	7.059847	-0.590650	0.690868
H	5.910764	-1.888578	-1.107136
H	5.657011	-0.711167	1.767482
H	4.498505	-2.099095	-0.049836
H	-1.606696	-0.835025	3.186186
H	-2.587705	-0.253563	0.921206
H	-3.287099	1.094490	-0.439887
H	-3.442684	-0.949974	-1.739727
H	-4.099637	0.239889	-2.879289
H	-1.914220	-1.333709	-3.552437
H	-0.147581	-0.754758	-2.107913
H	-0.485659	1.944621	-3.510271
H	-2.047034	0.327649	-4.124229
H	-2.789643	2.040248	-1.838780
H	-0.003335	-0.501041	2.518935
H	-0.003773	-0.392034	-0.009721
H	2.589262	2.429557	-0.529175
H	4.283982	-1.437637	-1.682132
H	0.563259	0.611899	-4.003902
H	5.189935	0.886911	-2.240861
H	0.895517	2.500402	-1.770070
C	-0.986809	-2.220996	1.678801
C	-0.990566	-4.776416	0.530097
C	0.183417	-2.761944	1.147071
C	-2.156783	-2.982363	1.630822
C	-2.161648	-4.248820	1.063459
C	0.178982	-4.029936	0.574675
H	1.110862	-2.186632	1.175359
H	-3.079721	-2.577698	2.053204
H	-3.084051	-4.830466	1.041319
H	-0.990940	-5.771470	0.083900
H	1.103594	-4.433725	0.161016
H	-1.412787	1.518004	2.972792

H-bonded-product-minor-SS-cat

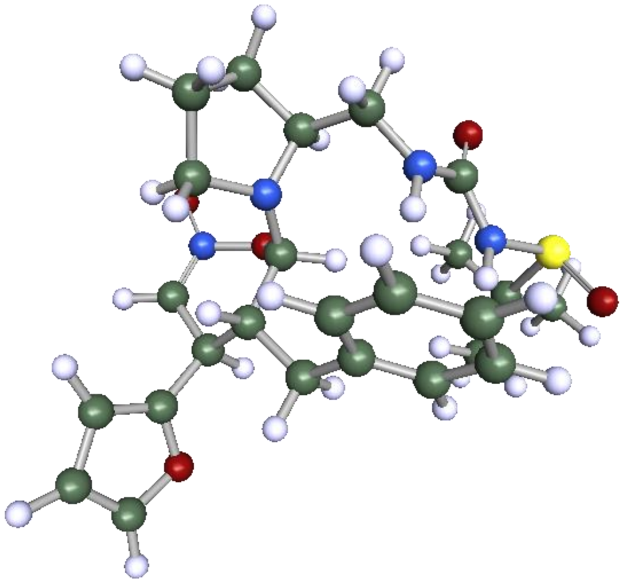

Geometry optimization, frequency calculation: PBEh-3c/def2-SV(P)

SFC energy	-1961.3594854 Hartree
Chemical potential	0.522772 Hartree
Entropy	0.000342 Hartree/K
Inner energy	0.623820 Hartree
In(Qtrans)	19.9343
In(Qrot)	17.1991
In(Qvib)	34.9254
ZPE	0.590809 Hartree
Enthalpy	0.624764 Hartree

Single point energy: M06-2X/def2-TZVP

SCF energy	-1966.26982049 Hartree

Cartesian coordinates

N	-1.113229	1.713613	-0.718225
C	-2.354919	2.448083	-0.487571
C	-2.111188	3.805131	-1.136518
C	-1.206529	3.453548	-2.310349
C	-0.280011	2.393064	-1.719530
C	0.975406	3.005315	-1.090943
N	1.786343	2.036940	-0.401791
C	2.672458	1.248161	-1.068418
O	3.052814	1.452164	-2.195043
N	3.086276	0.143102	-0.343645
S	4.481382	-0.733764	-0.771215
O	5.118326	-1.093888	0.522080
C	3.687986	-2.292292	-1.398653
C	4.871822	-3.186504	-1.762540
C	2.839776	-2.921258	-0.307727
C	2.871109	-1.935692	-2.632720
C	-1.039486	0.308942	-0.645229
C	-1.819190	-0.310499	0.536498

C	-0.898914	-0.929828	1.588841
C	-4.693381	-3.081791	2.523420
C	-5.458140	-1.962405	2.548415
C	-4.890803	-1.085289	1.574414
C	-3.830074	-1.748280	1.039439
O	-3.713857	-2.954498	1.616711
C	-2.824445	-1.366966	0.004801
C	-3.497044	-0.835351	-1.220673
N	-2.763230	-0.306612	-2.150709
O	-3.081955	0.195976	-3.205150
O	-1.412478	-0.358361	-1.886852
H	-4.572641	-0.833751	-1.381562
H	-2.254922	-2.278425	-0.256806
H	-5.232468	-0.089615	1.305161
H	-4.732430	-4.017203	3.074841
H	-6.323500	-1.780994	3.178668
H	5.489928	-3.424792	-0.886339
H	4.504837	-4.133466	-2.185374
H	3.413193	-3.065911	0.618528
H	2.479706	-3.907813	-0.637285
H	2.523720	-2.858370	-3.122154
H	1.952315	-2.311398	-0.083658
H	1.981110	-1.340723	-2.387419
H	-0.385232	-1.798672	1.146924
H	-2.424338	0.469218	1.018626
H	-3.234401	1.973360	-0.953363
H	-3.047853	4.293178	-1.436346
H	-1.602901	4.486361	-0.436584
H	-1.799594	3.010734	-3.123869
H	0.038383	1.686458	-2.500808
H	0.698051	3.788317	-0.369256
H	-0.664002	4.315193	-2.724542
H	-2.579665	2.551322	0.587441
H	-1.525274	-1.340017	2.398520
H	0.017157	0.008324	-0.591449
H	2.781774	0.005587	0.617140
H	3.458135	-1.368254	-3.369050
H	1.579144	3.485727	-1.872857
H	5.517863	-2.718796	-2.521298
H	1.497073	1.783467	0.532362
C	0.131712	-0.016419	2.201473
C	2.088687	1.619786	3.386802
C	-0.142821	1.315481	2.517213
C	1.400581	-0.513303	2.509227
C	2.373844	0.291988	3.092512
C	0.824559	2.124280	3.103863
H	-1.123079	1.740693	2.301736
H	1.637949	-1.554922	2.283869
H	3.363360	-0.119354	3.294235
H	2.849610	2.259269	3.833821

H	0.587907	3.163096	3.335939

Isolated RR-product

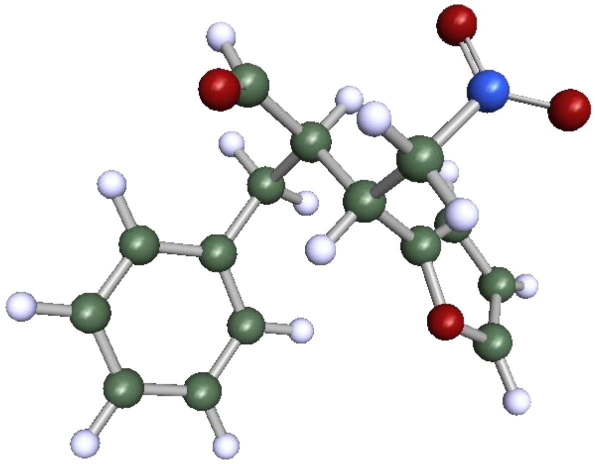

Geometry optimization, frequency calculation: PBEh-3c/def2-SV(P)

SFC energy	-933.4607713 Hartree
Chemical potential	0.237089 Hartree
Entropy	0.000226 Hartree/K
Inner energy	0.303608 Hartree
In(Qtrans)	19.0204
In(Qrot)	15.4418
In(Qvib)	17.7625
ZPE	0.286399 Hartree
Enthalpy	0.304552 Hartree

Single point energy: M06-2X/def2-TZVP

SCF energy	-936.12893716 Hartree

Cartesian coordinates

C	2.344567	0.868390	-0.980698
C	0.909937	0.435612	-1.154957
C	0.009146	1.606079	-0.674345
C	-2.659302	-2.389883	-0.243311
C	-2.882729	-1.836520	-1.460375
C	-1.685564	-1.129666	-1.788678
C	-0.839356	-1.307264	-0.738596
O	-1.431206	-2.074838	0.191674
C	0.577036	-0.903517	-0.484469
C	1.513526	-2.079569	-0.818845
N	1.443003	-2.456420	-2.258362
O	0.929736	-3.496941	-2.544207
O	1.899902	-1.670347	-3.045962
H	1.217422	-2.962361	-0.242198
H	0.701851	-0.769683	0.602784
H	-1.486436	-0.568057	-2.696955
H	-3.266757	-3.010407	0.409835

H	-3.784761	-1.921796	-2.058531
H	-0.996430	1.463228	-1.089495
H	0.388664	2.535837	-1.128736
H	2.624006	1.735898	-1.631038
C	-0.081132	1.762869	0.819401
C	-0.244033	1.999267	3.608114
C	0.924794	2.404811	1.542491
C	-1.176789	1.254262	1.517519
C	-1.258314	1.368567	2.899614
C	0.846824	2.520989	2.923933
H	1.783651	2.833071	1.023027
H	-1.985483	0.767383	0.969560
H	-2.124159	0.964447	3.424923
H	-0.306135	2.090623	4.692806
H	1.643962	3.027280	3.468997
O	3.135758	0.406128	-0.215197
H	2.558899	-1.838480	-0.595842
H	0.755902	0.371109	-2.243983

Isolated SR-product

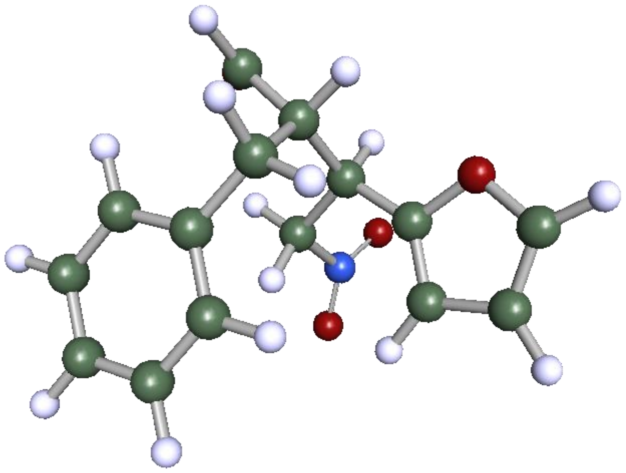

Geometry optimization, frequency calculation: PBEh-3c/def2-SV(P)

SFC energy	-933.4598453 Hartree
Chemical potential	0.239000 Hartree
Entropy	0.000220 Hartree/K
Inner energy	0.303533 Hartree
In(Qtrans)	19.0204
In(Qrot)	15.4157
In(Qvib)	15.7766
ZPE	0.286411 Hartree
Enthalpy	0.304478 Hartree

Single point energy: M06-2X/def2-TZVP

SCF energy	-936.12811706 Hartree

Cartesian coordinates

C	1.666760	-0.329502	-2.389405
C	1.600217	0.019887	-0.922565
C	0.943087	1.393067	-0.689739
C	2.493459	-0.224820	3.155223
C	1.209930	-0.252945	3.586807
C	0.424772	-0.619810	2.449376
C	1.302026	-0.788576	1.423192
O	2.551538	-0.546335	1.853489
C	1.144484	-1.147109	-0.019155
C	-0.267290	-1.651880	-0.292341
N	-0.552078	-2.928157	0.445873
O	-1.648649	-3.048960	0.913500
O	0.311844	-3.754735	0.487317
H	-0.396957	-1.899185	-1.353868
H	1.827073	-1.982842	-0.241264
H	-0.652493	-0.752091	2.419096
H	3.436539	-0.007263	3.649111
H	0.857219	-0.046166	4.592638
H	1.426151	2.115047	-1.369910
H	2.668747	0.183027	-0.680017
H	1.202228	1.730459	0.322812
H	2.005144	0.525213	-3.031539
C	-0.550733	1.453435	-0.866977
C	-3.340035	1.495778	-1.157348
C	-1.380951	1.687900	0.228739
C	-1.142848	1.263845	-2.116392
C	-2.523925	1.280007	-2.261247
C	-2.763204	1.707324	0.088170
H	-0.935497	1.846643	1.212241
H	-0.524314	1.106630	-3.002086
H	-2.965632	1.125043	-3.245964
H	-4.424314	1.505034	-1.269850
H	-3.393359	1.886956	0.959608
O	1.443630	-1.397311	-2.873360
H	-1.052667	-0.947411	-0.004465

6. References

1. Castán, A.; Badorrey, R.; Gálvez, J. A.; López-Ram-de-Víu, P.; Díaz-de-Villegas, M. D. *Org. Biomol. Chem.* **2018**, *16*, 924-935.
2. Feu, K. S.; de la Torre, A. F.; Silva, S.; de Moraes Junior, M. A. F.; Corrêa, A. G.; Paixão, M. W. *Green Chem.* **2014**, *16*, 3169-3174.
3. Ormandyová, K.; Bilka, S.; Mečiarová, M.; Šebesta, R. *ChemistrySelect* **2019**, *4*, 8870-8875.
4. Poláčková, V.; Čmelová, P.; Górová, R.; Šebesta, R. *Monats. Chem.* **2018**, *149*, 729-736.
5. Hu, X.; Wei, Y.-F.; Wu, N.; Jiang, Z.; Liu, C.; Luo, R.-S. *Tetrahedron: Asymmetry* **2016**, *27*, 420-427.

6. Čmelová, P.; Vargová, D.; Šebesta, R. *J. Org. Chem.* **2021**, *86*, 581-592.
7. TURBOMOLE V7.5, TURBOMOLE GmbH, Karlsruhe, 2019.
8. Balasubramani, S. G.; Chen, G. P.; Coriani, S.; Diedenhofen, M.; Frank, M. S.; Franzke, Y. J.; Furche, F.; Grotjahn, R.; Harding, M. E.; Hättig, C.; Hellweg, A.; Helmich-Paris, B.; Holzer, C.; Huniar, U.; Kaupp, M.; Marefat Khah, A.; Karbalaei Khani, S.; Müller, T.; Mack, F.; Nguyen, B. D.; Parker, S. M.; Perlt, E.; Rappoport, D.; Reiter, K.; Roy, S.; Rückert, M.; Schmitz, G.; Sierka, M.; Tapavicza, E.; Tew, D. P.; van Wüllen, C.; Voora, V. K.; Weigend, F.; Wodyński, A.; Yu, J. M. *J. Chem. Phys.* **2020**, *152*, 184107.
9. Grimme, S.; Brandenburg, J. G.; Bannwarth, C.; Hansen, A. *J. Chem. Phys.* **2015**, *143*, 054107.
10. Schäfer, A.; Horn, H.; Ahlrichs, R. *J. Chem. Phys.* **1992**, *97*, 2571-2577.
11. Zhao, Y.; Truhlar, D. G. *Theor. Chem. Acc.* **2008**, *120*, 215-241.
12. Weigend, F.; Ahlrichs, R. *Phys. Chem. Chem. Phys.* **2005**, *7*, 3297-3305.
